# Accumulating computational resource usage of genomic data analysis workflow to optimize cloud computing instance selection

**DOI:** 10.1093/gigascience/giz052

**Published:** 2019-04-24

**Authors:** Tazro Ohta, Tomoya Tanjo, Osamu Ogasawara

**Affiliations:** 1 Database Center for Life Science, Joint Support-Center for Data Science Research, Research Organization of Information and Systems, Mishima, Shizuoka 411–8540, Japan; 2 National Institute of Informatics, Research Organization of Information and Systems, Tokyo 101–8430, Japan; 3 DNA Data Bank of Japan, National Institute of Genetics, Research Organization of Information and Systems, Mishima, Shizuoka 411–8540, Japan

**Keywords:** high-throughput nucleotide sequencing, cloud computing, Common Workflow Language

## Abstract

**Background:**

Container virtualization technologies such as Docker are popular in the bioinformatics domain because they improve the portability and reproducibility of software deployment. Along with software packaged in containers, the standardized workflow descriptors Common Workflow Language (CWL) enable data to be easily analyzed on multiple computing environments. These technologies accelerate the use of on-demand cloud computing platforms, which can be scaled according to the quantity of data. However, to optimize the time and budgetary restraints of cloud usage, users must select a suitable instance type that corresponds to the resource requirements of their workflows.

**Results:**

We developed CWL-metrics, a utility tool for cwltool (the reference implementation of CWL), to collect runtime metrics of Docker containers and workflow metadata to analyze workflow resource requirements. To demonstrate the use of this tool, we analyzed 7 transcriptome quantification workflows on 6 instance types. The results revealed that choice of instance type can deliver lower financial costs and faster execution times using the required amount of computational resources.

**Conclusions:**

CWL-metrics can generate a summary of resource requirements for workflow executions, which can help users to optimize their use of cloud computing by selecting appropriate instances. The runtime metrics data generated by CWL-metrics can also help users to share workflows between different workflow management frameworks.

## Background

Improvements in the accuracy and quantity capacity of DNA sequencing technology mean that various sequencing methods are now available to measure different genomic features. Each method produces a massive amount of nucleotide sequence data, which require different data processing approaches [1]. Bioinformatics researchers develop data analysis tools for each sequencing technique, and they frequently publish implementations as open source software [2]. To begin data analysis, researchers must select the tools appropriate for their experimental design and install them to their computing environment.

Installing open source tools in one's computational environment is, however, not always straightforward. Tools created by different developers and using different programming frameworks require different prerequisites, which forces researchers to follow the instructions provided by the developer of each tool. Installing various items of software in one environment can also cause software dependency conflicts that are hard to resolve. Even if all of the tools required for the analysis can be successfully installed, it is a burden to maintain the environment and keep all of the tools working as expected. Events such as changes or updates to the hardware, operating system, or software libraries can also break the environment. Therefore, management of the data analysis environment becomes increasingly more complex when a project requires many tools to perform genomic data analysis. In addition, the high cost of setting up an environment can prevent the scaling of computational resources. This difficulty means that researchers depend on using their existing computing platform, and data processing jobs are limited to that resource.

Container virtualization technology, represented by Docker, enables users to create a software runtime environment that is isolated from the host machine [3]. This technology, which is becoming increasingly popular in the biomedical research domain, is a promising way to solve the problem of installing software tools [4]. Along with the containers, using workflow description and execution frameworks, such as those from the Galaxy project [5] or the Common Workflow Language (CWL) project [6], lowers the barrier to deploy a data analysis environment to a new computing environment. Moreover, workflows that are described in a standardized format can help researchers to easily share the environment with collaborators. Consequently, the improved portability of the data analysis environment has made on-demand cloud infrastructure an appealing option for researchers.

On-demand cloud infrastructure is beneficial in many aspects of genome science research because users can increase or decrease the number of computing instances required without having to maintain hardware as the amount of data from laboratory experiments changes [7]. For example, some sequencing applications require data analysis software that uses a considerable amount of memory, but individual research projects often cannot afford to buy a large-scale computing platform. Users can save money by using a pay-per-use on-demand cloud platform.

To use an on-demand cloud computing environment efficiently in terms of time and economic cost, it is essential to select a suitable computing unit—a so-called “instance type”—from the many options offered by cloud service providers. For example, Amazon Web Services (AWS), one of the most popular cloud service providers, offers instance types of different scales for 5 categories (general purpose, compute optimized, memory optimized, accelerated computing, and storage optimized) [8]. Each data analysis tool has a different minimum requirement of computational resources, such as memory or storage, and this requirement can change according to input parameters. Executing data analysis workflows on an instance without enough computational resources can result in a runtime failure or unexpected outputs. For example, tools that assemble short reads to construct a genome by way of a de Bruijn graph usually have a long processing time and require a large amount of memory. If the required amount of memory were to be wrongly estimated, the process might fail after a few days of execution, wasting time and money. Thus, to select a suitable instance type, users must know the minimum amount of computational resources required to execute their workflows.

To optimize the selection of instance type in terms of processing time or running cost, users must compare runtime metrics for workflow executions across environments with different computational specifications. Here, we present CWL-metrics, a system that accumulates runtime metrics for workflow executions, with information about the workflow and the machine environment. CWL-metrics works with cwltool, the reference implementation of CWL, using the workflow's input files and parameters to provide a summary of runtime metrics, including usage of central processing units (CPUs), memory, and storage input/output (I/O). This information will help users to select the proper cloud instance for their workflows.

## Results

### Implementation of CWL-metrics

**Figure 1:**
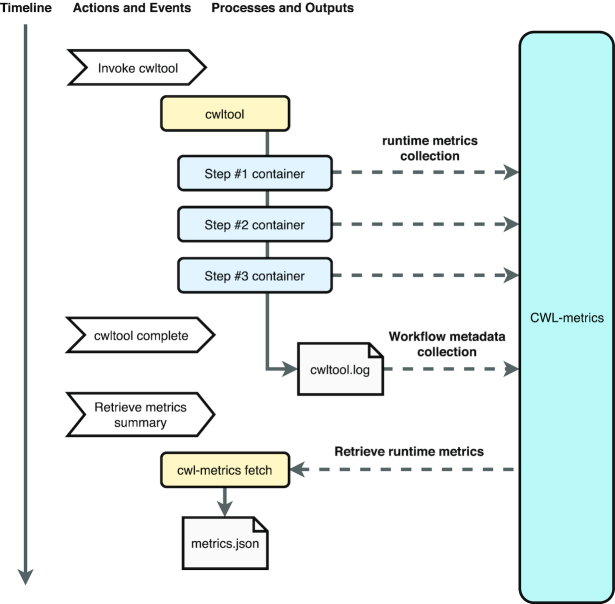
The container runtime metrics collection procedure using CWL-metrics. CWL-metrics was designed to automatically capture runtime metrics of workflow steps. After initializing the system, users need only to run a workflow by cwltool to start capturing metrics data. The system collects the runtime metrics of containers, and then workflow metadata are captured when the workflow process has finished. To retrieve runtime metrics, the cwl-metrics command can produce output summary data in JavaScript Object Notation (JSON) or tab-separated values (TSV) format.

We designed CWL-metrics to capture workflow-related runtime metrics data, described in CWL [6], a standardized language for workflow description developed by an open source community. The system has been designed in such a way that users do not need to perform any configurations to capture runtime metrics. Fig. 1 shows how runtime metrics are collected by CWL-metrics. To start collecting metrics data, users need only to install the system, and then run their workflows with cwltool [9], with options to increase log level and log file redirection to a file. Only a few prerequisites are required to install CWL-metrics, and these are easy to install with package managers including git, curl, perl, docker, and docker-compose. Once installed, the system automatically fetches the modules and starts monitoring the processes running on the host machine. Once the system detects a cwltool process, it automatically starts to collect runtime metrics via Docker application programming interface (API), and environmental information from the host machine. CWL-metrics also captures the log file generated by the cwltool command line to extract workflow metadata, such as input files and input parameters.

**Figure 2:**
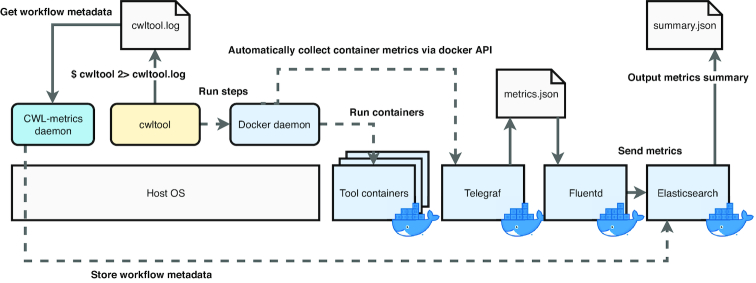
Components of CWL-metrics and working processes. CWL-metrics comprises a daemon process and several Docker containers on the host machine. The process and containers keep running until the system is terminated. Once a cwltool process starts running on the same machine, the CWL-metrics system monitors the process to obtain the list of workflow step containers and log files. Every 60 seconds, the Telegraf container attempts to access the Docker daemon to obtain runtime metrics data from running containers. The Fluentd container sends the runtime metrics data collected by Telegraf to the Elasticsearch container. The CWL-metrics daemon process captures the cwltool log file and sends workflow metadata to Elasticsearch.

To capture and store information from multiple data sources, CWL-metrics launches multiple components as Docker containers (Fig. 2). These components are automatically fetched by the system and keep running on the host machine after initialization to support the data collection. The Telegraf container collects runtime metrics data from the Docker API every 60 seconds and sends the data to the Elasticsearch container. The Elasticsearch container provides data storage and the data access API. CWL-metrics automatically launches and stops these components on the single host machine. To collect metrics data for workflows running on multiple instances, users must install CWL-metrics on each instance and manually assemble the summary data after the metrics data have been captured. Users can specify an Elasticsearch server on a different host to be a central data store by setting the environment variables ES_HOST and ES_PORT before initializing CWL-metrics.

**Figure 3:**
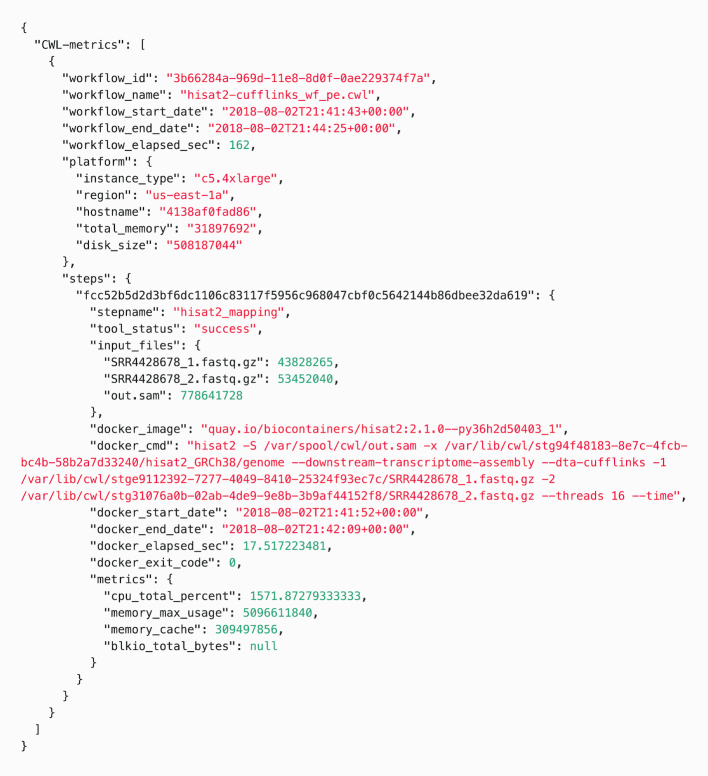
An example of runtime metrics data summarized by CWL-metrics. CWL-metrics can produce a JSON-formatted data output file, which includes workflow metadata, tool container metadata, and tool container runtime metrics. The workflow metadata appears once for 1 workflow run with data of multiple steps in the “steps” key; for brevity, this example shows only 1 step in the workflow. Each step has a name, exit status, input files with file size, and details of the Docker container. Runtime metric values may be null for short steps because CWL-metrics collects these metrics with a 60-second interval.

To access and analyze the data collected by CWL-metrics, the command "cwl-metrics" returns the data in JavaScript Object Notation (JSON; Fig. 3) or tab-separated values (TSV) formats. The JSON format contains workflow metadata such as the name of the workflow and the start and end times of workflow execution. It also contains information about the environment, including the total amount of memory and the size of storage available on the machine. The "steps" field of the JSON format file contains information about the runtime metrics, the executed container, and the input files and parameters. Users can parse the data to analyze the performance of the execution of a tool or of the whole workflow. The TSV format provides basic information for each container execution so that the metrics data of different steps can be easily compared.

### Using CWL-metrics to capture runtime metrics of RNA-sequencing workflows

To demonstrate the capture and analysis of workflow runtime metrics, we used 7 example RNA-sequencing (RNA-Seq) quantification workflows to analyze the optimal instance type for each one. Each of the 7 workflows (see Table 1) was run for 9 public human RNA-Seq datasets with different read lengths and numbers of reads (Table 2), on 6 types of AWS Elastic Compute Cloud (EC2) services (Table 3). CWL-metrics was used to capture runtime metrics data for each combination. Each workflow description has 2 options for read layout: single-end and paired-end. To select workflows, we chose 2 read-mapping tools, STAR [10] and Hisat2 [11], with 2 transcriptome assembly and read count programs, Cufflinks [12] and StringTie [13]. We also used 2 popular tools with different alignment approaches, Kallisto [14] and Salmon [15]. For comparison, we also used TopHat2 [12], a program that was previously one of the most popular transcript expression analysis tools but which has now been superceded by HISAT2 (according to an announcement in February 2016 that TopHat2 “is now largely superseded by HISAT2, which provides the same core functionality (i.e., spliced alignment of RNA-Seq reads), in a more accurate and much more efficient way”) [16]. Metrics data were collected 5 times for each combination of workflow, input data, and instance type. Our analysis does not include runs for the workflows STAR-cufflinks and STAR-stringtie on instance types with <30 GB memory because these runs failed.

**Table 1:** Components of the RNA-Seq quantification workflows

Workflow name	Steps	CWL definition files
TopHat2-Cufflinks	download-sra, pfastq-dump, tophat2-mapping, cufflinks	[17]
HISAT2-Cufflinks	download-sra, pfastq-dump, hisat2-mapping, samtools_sam2bam, samtools_sort, cufflinks	[18]
HISAT2-StringTie	download-sra, pfastq-dump, hisat2-mapping, samtools_sam2bam, samtools_sort, stringtie	[19]
Star-Cufflinks	download-sra, pfastq-dump, star-mapping, samtools_sam2bam, samtools_sort, cufflinks	[20]
Star-StringTie	download-sra, pfastq-dump, star-mapping, samtools_sam2bam, samtools_sort, stringtie	[21]
Kallisto	download-sra, pfastq-dump, kallisto-quant	[22]
Salmon	download-sra, pfastq-dump, salmon-quant	[23]

We described 7 different RNA-Seq quantification workflows in CWL. Each workflow description has 2 different options for read layout: single-end and paired-end. We selected 2 major read-mapping tools, STAR and Hisat2, with 2 transcriptome assembly and read count programs, Cufflinks and StringTie. We also used 2 popular tools with different quantification approaches, Kallisto and Salmon. For comparison, we added TopHat2, one of the most popular programs.

**Table 2:** Read characteristics of processed RNA-Seq data

SRA Run ID	Read length	No. of reads per strand	BioSample ID	Sample description	Sequencing instrument
SRR4250750	50	1,000,425	SAMN05779985	Cultured embryonic stem cells	Illumina HiSeq 2500
SRR5185518	50	5,008,398	SAMN06239034	Cultured embryonic stem cells	Illumina HiSeq 2500
SRR2932901	50	10,017,495	SAMN04211783	Fetal lung fibroblasts	Illumina HiSeq 2500
SRR4428678	75	1,043,870	SAMN05913930	Embryonic stem cell−derived macrophage	Illumina HiSeq 4000
SRR4241930	75	5,004,985	SAMN05770731	Primordial germ cell−like cells	Illumina HiSeq 2000
ERR204893	75	10,234,883	SAMEA1573291	Lymphoblastoid cell line	Illumina HiSeq 2000
SRR5168756	100	1,006,868	SAMN06218220	Subcutaneous metastasis	Illumina HiSeq 2500
SRR5023408	100	5,004,554	SAMN06017954	Primary breast cancer	Illumina HiSeq 2500
SRR2567462	100	10,007,044	SAMN04147557	Prostate cancer cells LNCaP	Illumina HiSeq 2500

We chose 9 different RNA-Seq datasets from the Sequence Read Archive (SRA), a public high-throughput sequencing repository. To compare their performance, each sequence was selected to be different in terms of read length and total number of reads. All data are from human samples sequenced by the Illumina HiSeq platform.

**Table 3:** Machine specifications of the AWS EC2 instance types used for metrics collection

Instance type	Category	Virtual CPU	ECU	Memory (GB)	Linux/UNIX Usage ($/hour)
m5.2xlarge	General purpose	8	31	32	0.384
m5.4xlarge	General purpose	16	60	64	0.768
c5.2xlarge	Compute optimized	8	34	16	0.340
c5.4xlarge	Compute optimized	16	68	32	0.680
r5.2xlarge	Memory optimized	8	31	64	0.504
r5.4xlarge	Memory optimized	16	60	128	1.008

To compare the performance of workflow runs on different computing platforms, we selected 3 AWS EC2 categories: general purpose, compute optimized, and memory optimized. We further selected 2 different instance types from those 3 categories, according to the number of virtual CPUs, 2xlarge and 4xlarge, with 8 and 16 CPU cores, respectively. EC2 Compute Unit (ECU) indicates the number of cores and the number of units per core. Instance usage prices are as of 14 August 2018 for on-demand use in the northern Virginia region of the USA. Prices do not include charges for storage, network usage, or other AWS features.

Table 4 shows the summary of runtime metrics, processing duration, and the calculated cost of instance usage per run for 2 workflows, HISAT2-Cufflinks and TopHat2-Cufflinks. The fastest processing time was achieved by the HISAT2-Cufflinks workflow run on the c5.4xlarge instance, but the cheapest execution was achieved with the HISAT2-Cufflinks workflow on the c5.2xlarge instance. This indicates that there is a trade-off between processing time and financial cost when running workflows on cloud instances. Each research project prioritizes time and cost differently, and this determines how the project will optimize instance selection. Table 4 also shows the potential loss of time or money when an inappropriate instance type is selected. For example, if the r5.4xlarge instance was used to run the HISAT2-cufflinks workflow, it would be 7% slower than if c5.4xlarge was used, and ∼1.6 times more expensive per sample. The longer the execution time, the greater the impact of a failure to optimize instance type.

**Table 4:** Comparison of the runtime metrics generated from TopHat2 and HISAT2

Workflow name	Instance type	Workflow duration (seconds)	Maximum CPU usage (%)	Total amount of memory (bytes)	Total amount of memory cache (bytes)	Total amount of block IO (bytes)	Cost per run ($)
HISAT2-Cufflinks	c5.2xlarge	1,014.5	796.83	10,033,995,776	5,183,479,808	4,748,816,384	0.0958
HISAT2-Cufflinks	c5.4xlarge	778	1,595.03	9,163,902,976	4,314,202,112	1,204,879,360	0.1470
HISAT2-Cufflinks	m5.2xlarge	1,013	799.09	11,254,398,976	6,396,575,744	1,204,858,880	0.1081
HISAT2-Cufflinks	m5.4xlarge	846	1,538.40	11,802,640,384	6,938,824,704	331,776	0.1805
HISAT2-Cufflinks	r5.2xlarge	1,015	798.21	10,912,165,888	6,065,545,216	3,608,539,136	0.1421
HISAT2-Cufflinks	r5.4xlarge	834	1,588.40	9,973,350,400	5,116,166,144	0	0.2335
TopHat2-Cufflinks	c5.2xlarge	5,139	797.85	12,310,124,544	8,869,050,368	12,343,222,272	0.4854
TopHat2-Cufflinks	c5.4xlarge	3,695	1,587.47	15,879,102,464	7,833,452,544	1,204,891,648	0.6979
TopHat2-Cufflinks	m5.2xlarge	5,579	799.55	15,149,662,208	9,395,200,000	51,970,048	0.5951
TopHat2-Cufflinks	m5.4xlarge	3,981	1,595.22	15,875,092,480	7,913,992,192	49,848,320	0.8493
TopHat2-Cufflinks	r5.2xlarge	5,487	798.60	15,152,807,936	9,492,783,104	49,848,320	0.7682
TopHat2-Cufflinks	r5.4xlarge	4,001	1,291.35	15,877,746,688	7,930,822,656	49,848,320	1.1203

We summarized runtime metrics values to compare 2 different workflows, HISAT2-Cufflinks and TopHat2-Cufflinks. All runs used input data SRR2567462. The read length was 100 base pairs, the number of reads was 10,007,044 and the read layout was single-end. Data are workflow duration in seconds, the CPU usage in percentage, the total amount of memory in bytes, the total amount of cache in bytes, the total amount of block input/output (I/O) in bytes, and the cost per run in US dollars. We calculated the median metrics values for 5 iterations of the workflow. Values can be zero for short steps because CWL-metrics collects these metrics at 60-second intervals.

**Figure 4:**
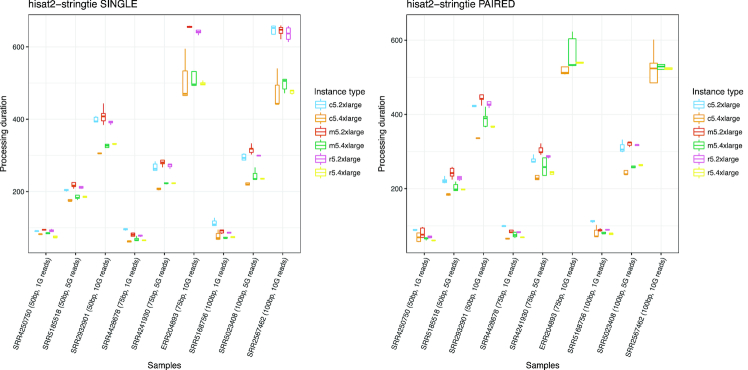
Box plot showing the distribution of per sample processing duration using the HISAT2-StringTie workflow. We plotted the processing durations of workflow runs, excluding data download time. The x-axis shows the Sequence Read Archive (SRA) Run ID of samples used as input data, with read lengths and numbers of reads. The y-axis shows the workflow processing duration in seconds. Values are separated and colored by the type of instance used. Some runs on specific instance types were excluded from the plots because they failed to execute. Each combination of sample and instance type was iterated 5 times to show the distribution of metrics data. The plot shows that read length and number of reads both affect the processing duration, and the differences between instance types are relatively small for smaller numbers of reads (1G reads), while instances with a greater number of CPU cores (*.4xlarge) have a shorter processing duration for 10 gigabase pair reads. The Permalink for the workflow in CWL viewer is: https://w3id.org/cwl/view/git/fb189fef3ddcb0d6619f9a22b4f57b880db654c0/workflows/hisat2-stringtie/single_end/hisat2-stringtie_wf_se.cwl.

Fig. 4 shows the processing duration results of the HISAT2-StringTie workflow. There are clear differences in processing time between the samples: samples with fewer reads have fewer differences between the instance types, while the runs on instance types with greater CPU usage (4xlarge) have a markedly shorter processing time for samples with larger numbers of reads. Each workflow run used as many CPU cores as were available in the environment; thus, the difference in duration can be explained by the difference in the number of threads. Read length and processing duration also have a strongly linear relationship. This correlation should be useful to estimate resource usage from the size of input data. Additional File 1 shows plots of the processing time of the different workflows for which similar results were found.

Conversely, comparison of the total amount of memory per input data in Additional File 2 invites a different interpretation. Unlike HISAT2 and TopHat2, Kallisto and Salmon did not show a strong correlation with memory usage for different sizes of input data. This indicates that users must know how the tool behaves before using it because resource usage depends on specific algorithms and their implementations.

**Figure 5:**
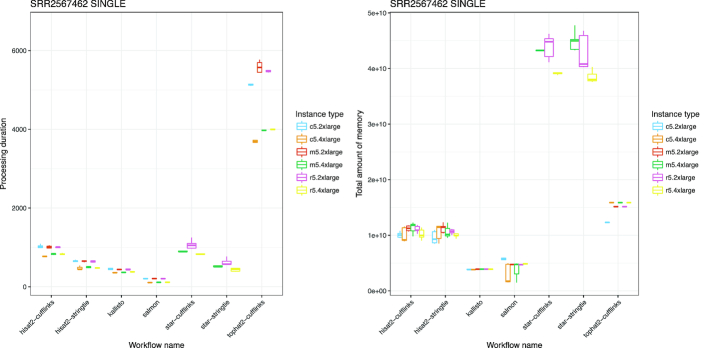
Box plot of processing duration and maximum memory usage of sample SRR2567462 per workflow. Values of processing duration exclude data download time. Both plots used values of workflow executions as a single-end input of SRR2567462. The x-axis shows workflow names, and the y-axis shows the processing duration in seconds and total memory usage in bytes. Each combination of workflow and instance type was iterated 5 times. The plot of processing duration shows that there is a difference in execution time between the TopHat2 workflow and others. While the differences in processing durations are relatively small, workflows using STAR aligner require 4 or 5 times more memory than HISAT2 workflows. These data suggest that users should know about the runtime metrics of their workflows before selecting an appropriate cloud instance type.

The runtime metrics data provided by CWL-metrics also helps to compare tools. Fig. 5 shows the differences in processing times between the workflows tested. Although users must consider the design concept and the individual strengths of the tools in order to select the most appropriate one for their research objectives, this result helps us to understand differences in the resource requirements of workflows used for similar purposes. For example, HISAT2 and STAR had almost the same processing time, but STAR uses far more memory. The plot of processing time also shows that TopHat2 is remarkably slower than the other tools.

## Discussion

CWL-metrics enables users to choose an appropriate cloud instance on which to run workflows, based on runtime metrics data. Metrics data summarized by workflow inputs, such as the number of threads to use or total file size of input data, inform more efficient cloud use for research projects. Each user can perform different analyses and visualizations depending on the input parameters of their choice. JSON and TSV data files—the outputs of CWL-metrics—can be parsed and used for visualization in any language.

CWL-metrics is applicable for most bioinformatics data analyses. However, there are cases for which the system does not work as effectively as expected. For example, the current implementation of CWL-metrics cannot capture precise runtime metrics data of a tool that scatters its processes to multiple computation nodes. Also, it cannot estimate the performance of software that uses hardware acceleration systems such as Graphics Processing Unit (GPU) because information for these specific architectures is not available via Docker API. Another limitation of the current implementation of CWL-metrics is that it does not record network usage because the Docker API does not provide network usage information per container [24]. Nevertheless, in our example using RNA-Seq workflows, we showed that CWL-metrics can provide beneficial information to help users decide on the best cloud infrastructure to use.

Other workflow operation frameworks exist that are able to capture runtime metrics, including Galaxy [25], Toil [26], Cromwell [27], and Nextflow [28]. These frameworks have features to enable the efficient execution of jobs that cwltool does not currently support, such as those run on a parallel computing platform. As an example, such workflow jobs might be submitted using a batch job queuing system such as the popular Univa Grid Engine [29]. This allows parallel execution of a workflow or workflow step, resulting in better performance in terms of processing duration. It is common practice in bioinformatics data analysis to parallelize a workflow or a workflow step.

We chose to use CWL as our workflow description framework, and its reference implementation cwltool as the workflow runner for the system, because CWL provides a way to share the workflow across different workflow systems. Once users have collected workflow runtime metrics with CWL-metrics, it is possible to execute the same workflow description using multiple workflow runner implementations. Fifteen implementations are listed as being able to support CWL [30]. While some implementations, including Galaxy, do not currently have the full ability to import and export CWL workflows, others, including Arvados, Toil, and Apache Airflow, are already available to users. If one wanted to use a workflow system that does not yet support CWL, the summary of runtime metrics collected through Docker containers is still valuable information for different frameworks that execute command line tools in a similar fashion.

We believe that CWL-metrics has the potential to support further applications in bioinformatics and data science. Our first target to improve the CWL-metrics tool is to add a feature to collect metrics data from parallelized job executions. We also expect to develop metrics collection for workflows that use different container technologies such as Singularity [31], which CWL-metrics does not currently support. Future implementations of CWL-metrics will cover different runtime environments for greater usability. We also aim to improve the implementation so that it can provide metrics—other than instance running time—related to the cost of cloud usage, e.g., total data transfer size or total disk usage of the workflow run, which users need to be able to estimate the cost of cloud infrastructure.

CWLProv, a subproject of the CWL project, provides provenance information for workflow executions to improve the reproducibility of workflows by tracking intermediate files and logs [32]. Provenance information helps users to track the inputs and outputs of workflow runs using file checksums but does not record details of the resource usage. Bundling CWL-metrics−generated runtime metrics data with provenance information will provide data needed for deployment, which helps users to reproduce runs in an appropriate computing environment.

It is essential for researchers to have a flexible computing environment that can be quickly scaled according to the amount of data processed. Rapid deployment of a data analysis environment to an appropriate cloud instance—supported by Docker, CWL, and CWL-metrics—is one way to achieve such computational scale-up, which brings a huge benefit for bioinformatics researchers.

## Potential Implications

The CWL project aims to support workflow description specification for all domains that work with data analysis pipelines. CWL-metrics is therefore able to contribute to other domains through the application of CWL. Sharing CWL workflows, with the metrics data captured by CWL-metrics, can help users to deploy them in appropriate cloud-based environments.

## Methods

### CWL-metrics software components

The CWL-metrics runtime metrics-capturing system comprises 5 software components: Telegraf [33], Fluentd [34], Elasticsearch [35], Kibana [36], and a Perl daemon script. Telegraf is an agent to collect runtime metrics of running containers via Docker API using the Telegraf Docker plugin. Fluentd works as a log data collector to send metrics data produced by Telegraf to the Elasticsearch server. Elasticsearch acts as a data store to accumulate runtime metrics data and workflow metadata, accepting JSON format data via an API end point. Kibana is a data-browsing dashboard for Elasticsearch to view raw JSON data, and to summarize and visualize data [37]. Telegraf, Fluentd, and Elasticsearch/Kibana launch as a set of containers during CWL-metrics initialization. CWL-metrics runs a Perl script, which monitors processes on the host machine to track cwltool processes. To send execution logs to the system, users must run cwltool with the specified option to output the cwltool log to a file. Once the script has found a cwltool process, it runs a function to collect workflow information via a debug output of the cwltool process, which is recorded in a file, “docker info” command output, a Docker container log via the “docker ps” command, and output of system commands to collect environment information. CWL-metrics collects data about the running time of workflow steps via the Docker container log. Thus, the duration equals the time of the life of the containers, which does not include the time of invocation by the workflow runner. CWL-metrics provides a command, "cwl-metrics," which allows users to start and stop the metrics collection system, and fetch summarized runtime metrics data in a specified format: JSON or TSV. The script used to launch the whole system, CWL-metrics installation instructions, and the documentation are available on GitHub [38].

### Packaging RNA-Seq tools and workflows

We created 7 different RNA-Seq quantification workflows to capture runtime metrics data and analyze cloud infrastructure performance. Each workflow begins with a tool to download sequence data from the Sequence Read Archive (SRA) [39] and then converts the SRA-formatted file to FASTQ format. Consequently, each pipeline performs sequence alignment to the reference genome sequence (HISAT2 [HISAT2, RRID:SCR_015530] [11], STAR [STAR, RRID:SCR_015899] [10], and TopHat2 [TopHat, RRID:SCR_013035] [12]), quasi-mapping (Salmon [Salmon, RRID:SCR_017036] [15]), or pseudo-alignment (Kallisto [Kallisto, RRID:SCR_016582] [14]) to the set of reference transcript sequences, then performs transcript quantification. Most of the tool containers used in the workflows are from the BioContainers [40] registry. We containerized those tools that were not available in the registry and uploaded them to the container registry service Quay [41]. We described tool definitions such as input and output of tool execution and the workflow procedures in CWL tool files, which are available on GitHub [42]. Each workflow has 2 options for sequence read layout: single-end and paired-end; thus, we created 14 workflow variants in total. Additional Table 1 shows the tool versions, the online location of the CWL tool files, and the original tool website locations.

### Selection of RNA-Seq workflow input sequence data from the public data repository

To analyze the effect of sequence data quality on workflow runtime performance, we chose 9 samples of different read lengths and numbers of reads from the public raw sequencing data repository, SRA (Table 2). We used the Quanto database [43] to select the data by filtering for read length (50, 75, or 100 base pairs) and approximate number of sequence reads (1,000,000, 5,000,000, or 10,000,000). We filtered the data using the string-match queries “organism: Homo sapiens,” “study type: RNA-Seq,” “read layout: PAIRED,” and “instrument model: Illumina HiSeq,” then manually picked data with sufficient descriptions from the returned results. Both single-end and paired-end workflows used the same dataset, although single-end workflows treated paired-end read file reads as 2 single-end read files. The version of the reference genome used was GRCh38 [44]. We downloaded the reference genome file from the University of California, Santa Cruz Genomics Institute's UCSC genome browser [45], and the Gencode gene annotation file version 28 from the Gencode website [46].

### Running workflows on AWS EC2

To evaluate the performance of running different RNA-Seq workflows, we selected instance types of 2 different sizes, 2xlarge and 4xlarge, from 3 categories: general purpose, compute optimized, and memory optimized, to run all workflows for all samples (Table 3). Each combination of instance type, workflow, and sample data was executed 5 times, while CWL-metrics was run on the same machine to capture the runtime metrics information. All workflow runs used Elastic Block Storage of General Purpose solid-state drive volumes as file storage. We downloaded all of the reference data used for workflows in advance. The source of the reference data used, and details of the scripts used to run workflows, are available online [42].

### Collecting and summarizing runtime metrics

After executing each workflow, we collected summarized metrics data from Elasticsearch using the "cwl-metrics fetch" command. Exported JSON data were parsed using a Ruby script to create data summarized per workflow run, loaded in a Jupyter Notebook [47] for further analysis. We calculated median values of metrics for replications using R language functions [48], and we created box plots using the ggplot2 package [49]. The notebook file is available on GitHub [50].

## Availability of source code and requirements

For CWL-metrics, the runtime metrics-capturing system:

Project name: CWL-metrics

Project home page: https://inutano.github.io/cwl-metrics/

doi:10.5281/zenodo.2583319

Operating system(s): Platform independent

Programming language: Perl v5.18.2 or higher

Other requirements: Docker 18.06.0-ce or higher and Docker Compose 1.22.0 or higher, cwltool 1.0.20180820141117 or higher

License: MIT

Any restrictions to use by non-academics: None

RRID:SCR_017076

For the scripts and notebook for visualization of this article:

Project name: cwl-metrics-manuscript

Project home page: https://github.com/inutano/cwl-metrics-manuscript

doi:10.5281/zenodo.2583314

Operating system(s): Platform independent

Programming language: Ruby 2.5.1 or higher

Other requirements: Docker 18.06.0-ce or higher

License: MIT

Any restrictions to use by non-academics: None

For the CWL definitions for tools and workflows used for benchmarking:

Project name: Pitagora CWL

Project home page: https://github.com/pitagora-network/pitagora-cwl

doi:10.5281/zenodo.2583023

Operating system(s): Platform independent

Programming language: Common Workflow Language v1.0

Other requirements: None

License: Apache 2.0

Any restrictions to use by non-academics: None

For the SRA data download tool:

Project name: download-sra

Project home page: https://github.com/inutano/download-sra

doi:10.5281/zenodo.2590835

Operating system(s): Platform independent

Programming language: Shell

Other requirements: wget, curl

License: MIT

Any restrictions to use by non-academics: None

For the SRA-formatted data parallel decompress tool:

Project name: pfastq-dump

Project home page: https://github.com/inutano/pfastq-dump

doi:10.5281/zenodo.2590841

Operating system(s): Platform independent

Programming language: Shell

Other requirements: SRA toolkit

License: MIT

Any restrictions to use by non-academics: None

## Availability of supporting data and materials

Source code and the documentation for the CWL-metrics system is available on GitHub [51]. The workflows and scripts used for the benchmarking experiment are published on GitHub [42]. The reference data used for workflow execution are available on Zenodo [52]. The intermediate and output files are also provided on Zenodo [53]. The dataset used for the visualizations of this article is available in figshare [54, 55]. The full summary data and visualization of Jupyter Notebook is available on GitHub [50]. Snapshots and Research Object bundles are also collected together in GigaDB [56]. A Jupyter Notebook file used to reproduce the visualizations in the article is also available [57, 58].

## Additional files

**Additional File 1:** Box plot of processing duration for all workflows. The x-axis shows SRA Run IDs of input data, with read length and numbers of reads. The y-axis shows the processing duration in seconds, excluding data download time. In most of the workflows tested, read length and the number of reads of input data affect the processing time. Workflows using STAR aligner require a large amount of memory; thus, executions on instance types with smaller amounts of memory have failed.

**Additional File 2:** Box plot of maximum memory usage for all workflows. The x-axis shows SRA Run IDs of input data, with read length and numbers of reads. The y-axis shows the maximum amount of memory used during the process in bytes. The distributions of values are large, especially for runs that complete quickly, probably because the 60-second interval of metrics capture was not able to return consistent values.

**Additional Table 1:** Versions and containers of tools used in the RNA-Seq workflows. We used 11 tools to construct 7 RNA-Seq quantification workflows. We packaged the 2 tools we developed, "download-sra" [41] and "pfastq-dump" [41], into containers ourselves. The Salmon container is available on its developer's build. The rest of the tools were obtained from the BioContainers registry. We wrapped all the tools as CWL CommandLineTool class files, and these are available on GitHub [42].

## Abbreviations

API: application programming interface; AWS: Amazon Web Services; CPU: central processing unit; CWL: Common Workflow Language; EC2: Elastic Compute Cloud; I/O: input/output; JSON: JavaScript Object Notation; RNA-Seq: RNA-sequencing; SRA: Sequence Read Archive; TSV: tab-separated values.

## Competing interests

The authors declare that they have no competing interests.

## Funding

This work was supported by the CREST program of the Japan Science and Technology Agency (grant No. JPMJCR1501).

## Authors’ contributions

T.O. and T.T. conceived and developed the methodology and software and conducted the investigation. T.O. visualized the data and wrote the manuscript. O.O. supervised the project. All authors read and approved the final version of the manuscript.

## Supplementary Material

GIGA-D-18-00427_Original_Submission.pdf

GIGA-D-18-00427_Revision_1.pdf

GIGA-D-18-00427_Revision_2.pdf

Response_to_Reviewer_Comments_Original_Submission.pdf

Response_to_Reviewer_Comments_Revision_1.pdf

Reviewer_1_Report_Original_Submission -- Kyle Hernandez, Ph.D. 11/18/2018 Reviewed

Reviewer_2_Report_Original_Submission -- Paolo Di Tommaso 12/9/2018 Reviewed

Reviewer_3_Report_Original_Submission -- Stian Soiland-Reyes 12/18/2018 Reviewed

Supplemental Files
