# Supplementary material for: Accumulating computational resource usage of genomic data analysis workflow to optimize cloud computing instance selection
Source: Gigascience. 2019 Apr 24;8(4):giz052. doi: 10.1093/gigascience/giz052 (PMC6479428; doi:10.1093/gigascience/giz052)
Supplement: GIGA-D-18-00427_Revision_1.pdf [file giz052_giga-d-18-00427_revision_1.pdf]

GigaScience

Accumulating computational resource usage of genomic data analysis workflow to optimize cloud computing instance selection

--Manuscript Draft--

**Manuscript Number:**	GIGA-D-18-00427R1
**Full Title:**	Accumulating computational resource usage of genomic data analysis workflow to optimize cloud computing instance selection
**Article Type:**	Research
**Funding Information:**	Core Research for Evolutional Science and Technology (JPMJCR1501) — Dr Osamu Ogasawara
**Abstract:**	Background<br>Container virtualization technologies such as Docker are popular in the bioinformatics domain as they improve portability and reproducibility of software deployment. Along with software packaged in containers, the workflow description standards Common Workflow Language also enable to perform data analysis on multiple different computing environments with ease. These technologies accelerate the use of on-demand cloud computing platform which can scale out according to the amount of data. However, to optimize the time and the budget on a cloud usage, users need to select a suitable instance type corresponding to the resource requirements of their workflows.<br>Results<br>We developed CWL-metrics, a utility tool for cwltool, the reference implementation of CWL, to collect runtime metrics of Docker containers and workflow metadata to analyze resource requirement of workflows. We demonstrate the analysis by using seven transcriptome quantification workflows on six instance types. The result showed instance type options of lower financial cost and faster execution time with required amount of computational resources.<br>Conclusions<br>The summary of resource requirements of workflow executions provided by CWL-metrics can help users to optimize the selection of cloud computing instances. The runtime metrics data also help users to share workflows among different workflow management frameworks.
**Corresponding Author:**	Tazro Ohta<br><br>JAPAN
**Corresponding Author Secondary Information:**	
**Corresponding Author's Institution:**	
**Corresponding Author's Secondary Institution:**	
**First Author:**	Tazro Ohta
**First Author Secondary Information:**	
**Order of Authors:**	Tazro Ohta<br>Tomoya Tanjo<br>Osamu Ogasawara
**Order of Authors Secondary Information:**	
**Response to Reviewers:**	GIGA-D-18-00427<br>Accumulating computational resource usage of genomic data analysis workflow to optimize cloud computing instance selection<br>Tazro Ohta; Tomoya Tanjo; Osamu Ogasawara<br>GigaScience

Powered by Editorial Manager® and ProduXion Manager® from Aries Systems Corporation

We deeply thank the reviewers for their positive comments and suggestions. Those suggestions have greatly improved the quality of our project. Our responses are below in-line (the reviewer's comments are in italics).

Response to the reviewers' comments:

Reviewer #1:

Overall the authors provide a useful utility to cwltool that allows for an easy to use collection of runtime metrics. These metrics can be used to make informed decisions on estimating cost and which VM flavors are most efficient. My biggest concern is that the language throughout the manuscript except for a small part of the discussion conveys the idea that this tool is CWL-specific. CWL is simply the specification for defining a workflow and does not define anyway to report logs or runtime metrics which are defined by specific CWL engines. Thus, CWL-metrics is actually dependent on the cwltool reference implementation and the way it currently outputs logs. To me, CWL-metrics is more like an enhancement utility to the cwltool package than a part of CWL itself. I think this is an important distinction to make, especially since cwltool currently only handles serial operation of highly parallelizable workflows. So, if you were really concerned about cost, you likely wouldn't use cwltool as an engine, but look into something like Cromwell, Cavatica (Seven Bridges), or use a GA4GH TES implementation like Funnel. Again, this tool is indeed useful as I have personally used it, I just think that it is important to better clarify this in the manuscript.

Response: We agree with the reviewer's assessment. We started this project to develop a method to provide runtime metrics of CWL workflows so users can share workflows with their resource requirements. The current implementation of CWL-metrics, however, as the reviewer indicates, is the utility tool for cwltool. This is just because the cwltool is the reference implementation of CWL, and will be maintained with the updates of the specification. Yet we think we need to increase the coverage of workflow runners so users can get more practical information. We agree that it is important to make the current status clear. To emphasize the CWL-metrics depends on cwltool, we changed the sentence in the abstract to "We developed CWL-metrics, a utility tool for cwltool, the reference implementation of CWL, to collect runtime metrics of Docker containers and workflow metadata to analyze resource requirement of workflows" and added "CWL-metrics works with cwltool, the reference implementation of CWL" in the last paragraph of the background section.

Related to this, the authors mention Nextflow and Galaxy among others; however, they don't mention WDL or Cromwell which I believe is much more closely related to CWL and I think merit mentioning, especially since Cromwell can run CWL workflows at scale and in parallel.

Response: In the original manuscript, we mentioned Galaxy, Toil, and nextflow because they are the runners that can collect runtime information like CWL-metrics. We added a new paragraph in the discussion section to mention Cromwell and the limitation of cwltool in parallel job execution.

The following are minor things that I believe need to be addressed. The authors use the term "alignment-like" when referring to tools like Kallisto and Salmon; however, I believe the appropriate term is "pseudo-alignment".

Response: The Kallisto is using the term "pseudo-alignment" in its paper and documentation, but the Salmon does not. The authors of Salmon call its algorithm "quasi-mapping" and did not use the "pseudo-alignment" (https://doi.org/10.1038/nmeth.4197). Therefore we used the term "alignment-like", but it is still confusing. We changed the sentence to call them software use "different alignment approaches".

In figures 4 and 5, I assume the duration y-axis is in seconds; however, this doesn't seem to be mentioned in the description or axis labels and is important especially for people unfamiliar with the workflows.

Powered by Editorial Manager® and ProduXion Manager® from Aries Systems Corporation

Response: We added the corresponding units to the Y-axis labels of the plots. Thank you for pointing out the issue.

Finally, I don't feel like the authors provided any clear "future features" they would like to work on (or have the community contribute to). For example, they mention some limitations (e.g., scatter/gather across nodes) that could be overcome by having a centralized service (could be containerized) that all workers post metrics to.

Response: We added a new paragraph in the discussion section with the future prospects including the support of parallelized job execution and the different container runtime like Singularity.

Reviewer #2:

In this paper the authors describe a system to collect execution runtime metrics for computational workflows described using the Common Workflow Language notation. They also provide a benchmark of different tools executed against different dataset to show the benefits for their approach.

The topic is quite interesting because, given the exponential growth of genomics data, there's a pressing need to optimise bioinformatics tools and workflows for better resources allocations and usage in order to optimise the overall costs of long running in-silico data analyses.

The paper is easy to read, well structured and informative. It's particularly interesting the benchmark comparing different genome sequence aligners resource usage.

The only point to make is that the manuscript would capture the interest of a broader audience if the authors would provide a more balanced comparison with similar technologies such as Galaxy and Nextflow. For example the authors mention that their system only work for specific a CWL implementation (cwl-runner) and require the usage of Docker compliant system along with the deployment of other third party tools (eg. Telegraph, Elasticsearch, etc.) which installation could be challenging the average workflow users. Nextflow implements a very similar feature as the one describe in this manuscript to collect, visualise and export the execution metrics. However it can be used irrespective the execution platform supported by the tool (ie. local execution, clusters and clouds), in a single node or multi-node deployment and does not require the installation of any third party software components, either with or without containerised execution (disclaimer, the writer is the creator of the Nextflow tool).

Response: We added a paragraph in the discussion section for a more clear comparison of runners in terms of parallel job execution. The main difference between the cwltool and the other runners that can collect runtime metrics is the ability to capture the parallelized workflow job. We described the limitation of the current implementation of CWL-metrics that depends on cwltool. We want to note that the CWL-metrics users do not need to install Telegraph or Elasticsearch by themselves because the system automatically fetches those components as Docker containers. The prerequisites of the system are git, curl, Perl, Docker, and Docker compose. We added a sentence to mention this in the "Implementation of CWL-metrics" in the result section.

Very minor note, the usage of a human friendly format and data units for time and memory (e.g. seconds or Mega/Giga bytes) values would make the charts more readable.

Response: We added the corresponding units to the Y-axis labels of the plots. Thank you for your suggestion.

- Paolo

Response: Thank you very much, Paolo! Taz :)

Reviewer #3:

This manuscript proposes cwl-metrics, a way to collect metrics from Common Workflow Language executions of bioinformatics tools using Docker containers.

The manuscript is submitted as a Technical Note, but the research is of such a high quality that this could even be a Research article had it also provided a broader Background and a Discussion with comparison of workflow metrics systems beyond CWL.

Response: We submitted this manuscript via the direct submission system through bioRxiv, which did not give us an article type selection. We suppose that the GigaScience editorial office made a decision to make this a Technical Note, but we would be happy to change the category to Research article if possible.

The authors have been diligent in reproducibility and been good practitioners of Open Science, recording rich details of their evaluations and providing installation scripts for not just the software but also the evaluation setup. Some small issues remain to make this truly reproducible.

The English language of the manuscript is however not of a good quality for publication, to a degree where this can be confusing. Knowing the authors work from the CWL community I have provided feedback within the annotated manuscript (attached PDF) using ISO5776 text proof notation.

See the detailed review PDF for further comments according to GigaScience review guidelines, in particular the reproducibility section.

My detailed review is also web-accessible at the (secret) URL https://gist.github.com/stain/30e49363238d5a35e26f9fb1a31ebf8e

Response: Thank you very much for the many practical suggestions and corrections in detail. The correction of English writing was very helpful. We greatly appreciate your help.

Minor Revisions required:

* English Language needs to be revised

Response: We modified the text following your suggestions and corrections.

* Missing DOIs for citations

Response: We added the DOIs to the reference section.

* Missing DOIs for code/workflows, not (just) GitHub links

Response: For our GitHub repositories, we assigned DOIs and used them in the reference section.

* License on CWL workflows/tools

Response: We added the license to the repository and to the individual files (https://github.com/pitagora-network/pitagora-cwl). The license is Apache-2.0 following the best practice of the Common Workflow Language project.

* Fix Reproducibility issue in Notebook (missing files)

Response: We uploaded the missing files to the Figshare and fixed the Notebook code to fix the issue. We also tested the Notebook correctly works on a new machine environment.

* License and source/upstream attribution for quay.io docker images

Response: We added the GPL-3.0 license for the Docker images we uploaded to

	Quay.io.<br><br>Other suggestions in this review are recommended, but not required.<br><br>Response: We also fixed documentations on GitHub to provide more details for workflows and test scripts we provide. The documentation for reference data we used for the benchmark is also available on the new GitHub repo (https://github.com/pitagora-network/pitagora-cwl). We uploaded the reference data index to Zenodo (https://doi.org/10.5281/zenodo.2587201). We also uploaded the intermediate files and final outputs of the workflows executed for the benchmarking to Zenodo (https://doi.org/10.5281/zenodo.2586546). Thank you very much again for your suggestions, we are sure that the CWL-metrics project is now more reproducible.
**Additional Information:**	
**Question**	**Response**
Are you submitting this manuscript to a special series or article collection?	No
**Experimental design and statistics**<br><br>Full details of the experimental design and statistical methods used should be given in the Methods section, as detailed in our Minimum Standards Reporting Checklist. Information essential to interpreting the data presented should be made available in the figure legends.<br><br>Have you included all the information requested in your manuscript?	Yes
**Resources**<br><br>A description of all resources used, including antibodies, cell lines, animals and software tools, with enough information to allow them to be uniquely identified, should be included in the Methods section. Authors are strongly encouraged to cite Research Resource Identifiers (RRIDs) for antibodies, model organisms and tools, where possible.<br><br>Have you included the information requested as detailed in our Minimum Standards Reporting Checklist?	Yes
**Availability of data and materials**	Yes

All datasets and code on which the conclusions of the paper rely must be either included in your submission or deposited in publicly available repositories (where available and ethically appropriate), referencing such data using a unique identifier in the references and in the "Availability of Data and Materials" section of your manuscript.<br><br>Have you have met the above requirement as detailed in our Minimum Standards Reporting Checklist?	

Manuscript
Click here to view linked References
Click here to access/download;Manuscript;CWL-metrics manuscript revise1.pdf

Accumulating computational resource usage of genomic data analysis workflow to optimize cloud computing instance selection

Tazro Ohta[1], Tomoya Tanjo[2], Osamu Ogasawara[3]

Affiliation

1. Database Center for Life Science, Joint Support-Center for Data Science Research, Research Organization of Information and Systems, Yata 1111, Mishima, Shizuoka 411-8540, Japan
2. National Institute of Informatics, Research Organization of Information and Systems, Tokyo 101–8430, Japan
3. DNA Data Bank of Japan, National Institute of Genetics, Research Organization of Information and Systems, Yata, Mishima 411-8540, Japan

Correspondence should be addressed to T.O. (t.ohta@dbcls.rois.ac.jp)

Abstract

Background

Container virtualization technologies such as Docker are popular in the bioinformatics domain as they improve portability and reproducibility of software deployment. Along with software packaged in containers, the workflow description standards Common Workflow Language also enable to perform data analysis on multiple different computing environments with ease. These technologies accelerate the use of on-demand cloud computing platform which can scale out according to the amount of data. However, to optimize the time and the budget on a cloud usage, users need to select a suitable instance type corresponding to the resource requirements of their workflows.

Results

We developed CWL-metrics, a utility tool for cwltool, the reference implementation of CWL, to collect runtime metrics of Docker containers and workflow metadata to analyze resource requirement of workflows. We demonstrate the analysis by using seven transcriptome quantification workflows on six instance types. The result showed instance type options of lower financial cost and faster execution time with required amount of computational resources.

Conclusions

The summary of resource requirements of workflow executions provided by CWL-metrics can help users to optimize the selection of cloud computing instances. The runtime metrics data also help users to share workflows among different workflow management frameworks.

Keywords

High-throughput nucleotide sequencing, Cloud computing, Common Workflow Language

Background

According to the improvement of DNA sequencing technology in accuracy and quantity, various sequencing methods are now available to measure different genomic features. Each method produces a massive amount of nucleotide sequence data that requires a different data processing approach [1]. Bioinformatics researchers develop data analysis tools for each sequencing technique, and they frequently publish implementations as open source software

[2]. To start data analysis, researchers need to select the tools according to their experimental design and install them to their computing environment.

Installing open source tools in one's computational environment is, however, not always straightforward. Tools developed by different developers and different programming frameworks require different prerequisites, which forces one to follow the instruction provided by each tool's developer. Installing various software in one environment also can cause a conflict of software dependencies that are hard to resolve. Even if one could successfully install all the tools required for the analysis, maintaining the environment where all the tools keep working as expected is also a burden. There are also many events that can break the environment such as changes or updates of hardware, operating system, or software libraries. Therefore, the complexity of data analysis environment management gets higher when a project performs genomic data analysis that requires many tools. The high cost of setting up an environment results in the prevention of scaling out the computational resources as well. The difficulty also brings researchers' dependency to the existing computing platform already set up, and the concentration of data processing jobs to the limited resource.

Container virtualization technology, represented by Docker, enables users to create a software runtime environment isolated from the host machine [3]. This technology that is getting popular also in the biomedical research domain is a promising method to solve the problem of installing software tools [4]. Along with the containers, using workflow description and execution frameworks such as those from the Galaxy project [5] or the Common Workflow Language (CWL) project [6] lower the barrier to deploy the data analysis environment to a new computing environment. Moreover, the workflows described in a standardized format can help researchers to share the environment with collaborators with ease. The improvement of portability of data analysis environment, consequently, has made on-demand cloud infrastructure an appealing option for researchers.

On-demand cloud is beneficial for most cases in genome science because users can increase or decrease the number of computing instances without maintaining hardware as the amount of data from laboratory experiments changes [7]. For example, some sequencing applications require data analysis software that uses a considerable amount of memory, but individual research projects often cannot afford a large scale computing platform. Users can budget

by using the on-demand cloud platform as most of the service providers charge per usage.

However, to use an on-demand cloud environment efficiently regarding time and economic cost, it is essential to select a suitable computing unit, so-called *instance type*, from many options offered by the cloud service providers. For example, Amazon Web Service (AWS), one of the popular cloud service providers, offers instance types of different scales for five categories (general purpose, compute optimized, memory optimized, accelerated computing, and storage optimized) [8]. Each data analysis tool has different minimum requirement of computational resources such as memory or storage, and it can change by input parameters. Executing data analysis workflows on an instance without enough computational resource may result in a runtime failure or unexpected outputs. For example, tools to assemble short reads to construct a genome by constructing a De Bruijn graph usually take a processing time and a large amount of memory. If one failed to estimate the required amount of memory, the process might fail after a few days of execution, wasting time and budget. Thus, to select a suitable instance type, users need to know the minimum amount of computational resource required by the execution of their workflows.

To optimize the instance type selection concerning processing time or running cost, users need to compare runtime metrics of workflow executions across environments of different computational specs. Here we present *CWL-metrics*, a system to accumulate runtime metrics of workflow executions with information of the workflow and the machine environment. CWL-metrics works with cwltool, the reference implementation of CWL, providing runtime metrics summary such as usage of CPU, memory, storage I/O with workflow's input files and parameters to help users to select the proper cloud instance for their workflows.

Results

Implementation of CWL-metrics

CWL-metrics is designed to capture runtime metrics data of workflows described in CWL [6], a workflow description specification developed by an open source community. We designed the system so that it does not require the users to perform any configurations to capture runtime metrics. Figure 1 shows the procedures of runtime metrics collection by CWL-metrics. To start collecting metrics, users only need to install the system, and then run their

workflows with *cwltool*, the CWL reference implementation [9] with options to increase log level and log file redirection to a file. The installation of CWL-metrics requires only several prerequisites that are easy to install with package managers including git, curl, perl, docker, and docker-compose. After the installation, the system automatically fetches the modules and starts monitoring the processes running on the host machine. Once the system detect a cwltool process, it automatically starts collecting runtime metrics via Docker API and environmental information from the host machine. CWL-metrics also captures the log file generated by cwltool command line to extract workflow metadata such as input files and input parameters.

To capture and store the information from multiple data source, CWL-metrics launches multiple components as Docker containers (Figure 2). These components are automatically fetched by the system, and keep running on the host machine after the initialization to support the data collection. The Telegraf container collects runtime metrics data from the Docker API every sixty seconds, and send the data to the Elasticsearch container. The Elasticsearch container provides data storage and the data access API. CWL-metrics automatically launches and stops these components on the single host machine. To collect metrics of workflows running on multiple instances,

users need to install CWL-metrics on each instance and manually assemble the summary data after the metrics data capture. Users can specify an Elasticsearch server on a different host as a central data store by setting environment variable ES_HOST and ES_PORT before initializing CWL-metrics.

To access and analyze the data collected by CWL-metrics, the command *cwl-metrics* return the data in JSON (Figure 3) or tab separated values (TSV) format. The JSON format contains workflow metadata such as the name of the workflow, the time of start and end of the workflow execution. It also has information of the environment including the total amount of memory and the size of storage available on the machine. The *steps* field of the JSON format contains information of the runtime metrics, the executed container, and the input files and parameters. Users can parse the data to analyze the performance of a tool execution or the whole workflow. The TSV format provides basic information for each container execution so that one can easily compare the metrics data of steps.

Using CWL-metrics to capture runtime metrics of RNA-Seq workflows

As an example use case to capture and analyze runtime metrics of workflows, we performed an analysis to optimize instance type selection for RNA-Seq quantification workflows. We ran 7 RNA-Seq workflows (Table 1) for 9 public human RNA-Seq data with different read length and number of reads (Table 2) on 6 types of Amazon Web Service (AWS) Elastic Compute Cloud (EC2) service (Table 3) to capture the runtime metrics with CWL-metrics for each combination. Each workflow description has two different options for read layout; single-end and paired-end. For the selection of workflows, we chose two read mapping tools STAR [10] and Hisat2 [11], with two transcriptome assembly and read count programs Cufflinks [12] and StringTie [13]. We also used two popular tools using different alignment approaches, Kallisto [14] and Salmon [15]. TopHat2 [12], the program which was one of the most popular transcript expression analysis tools, but announced on February 2016 that it is outdated on its website says "is now largely superseded by HISAT2 which provides the same core functionality (i.e. spliced alignment of RNA-Seq reads), in a more accurate and much more efficient way" [16], was added among them for comparing purpose. We performed metrics data collection 5 times for each combination of workflow, input data, and instance type. The workflows STAR-cufflinks and STAR-stringtie on the instance types

with less than 30GB memory have failed, thus the analysis does not include the runs.

Table 4 shows that the summary of runtime metrics, processing duration, and the calculated cost of instance usage per run for two workflows, HISAT2-Cufflinks and TopHat2-Cufflinks. The fastest processing time was one of the HISAT2-Cufflinks workflow run on the *c5.4xlarge* instance, but the execution at the cheapest cost was the HISAT2-Cufflinks workflow on the *c5.2xlarge* instance. It indicates that workflows on cloud instances can have a trade-off between processing time and financial cost. Each research project will prioritize time and cost differently, determining how the project will optimize instance selection. The table also shows the possibility of loss of time or money when one failed to choose a proper instance type. For example, if one used the *r5.4xlarge* instance to run the HISAT2-cufflinks workflow, it is 7% slower than *c5.4xlarge*, and about 1.6 times expensive per sample. The impact of an instance type optimization failure will be more serious the data processing jobs that take days or weeks.

Figure 4 shows the results of processing duration of the HISAT2-StringTie workflow. There are clear differences in processing time

between the samples, where the samples with smaller number of reads have less differences between the instance types, while the runs on instance types with more CPU (4xlarge) marked shorter processing time for the samples with the larger number of reads. Each workflow run used as many CPU cores as available on the environment; thus the difference in duration can be explained from the difference in number of threads. The read length and processing duration also have a strong linear relationship. This correlation should be useful to estimate the resource usage from the size of input data. Supplementary Figure 1 shows the plots of the processing time of the different workflows in which similar results were found.

On the other hand, the result of the comparison of the total amount of memory per input data in Supplementary Figure 2 invites a different interpretation. Unlike HISAT2 and TopHat2, Kallisto and Salmon did not show a strong correlation in memory usage for different sizes of input data. The result indicates that the users need to know the behavior of the tool beforehand since the resource usage depends rather on the algorithms and the implementations.

The runtime metrics data provided by CWL-metrics also helps to perform tool comparison. Figure 5 shows the difference in processing time between the used workflows. Although users need to consider the design concept and the individual strengths of the tools to select the most appropriate one for their research objectives, this result helps to understand the difference of the resource requirement of the workflows for similar purpose. For example, HISAT2 and STAR marked almost the same processing time, but STAR uses far more memory. The plot of the processing time also shows that TopHat2 is remarkably slower than the other tools.

Discussion

CWL-metrics enable users to choose an appropriate cloud instance for workflow runs based on the runtime metrics data. The metrics data summarized by workflow inputs, such as the number of threads to use or total file size of input data, informs more efficient cloud use for research projects. Each user might perform different analyses and visualizations depending on input parameters of their choice. Thus CWL-metrics outputs JSON and TSV data to be parsed and used for visualization by any language.

CWL-metrics is applicable for most cases in bioinformatics data analysis. However, there are cases that the system does not work as effectively as expected. For example, the current implementation of CWL-metrics cannot capture precise runtime metrics data of a tool that scatter its processes to multiple computation nodes. Also, it cannot estimate the performance of software that uses hardware acceleration systems such as GPU, since the information of those specific architectures is not available via Docker API. Another limitation of the current implementation of CWL-metrics is that it does not record network usage because the Docker API does not provide network usage information per container [17]. Nevertheless, in the example use case of RNA-Seq workflows, we showed CWL-metrics could provide beneficial information to help users to decide on how to use of cloud infrastructure.

There are also the other workflow operation frameworks that have functions to capture runtime metrics, such as Galaxy [18], Toil [19], Cromwell [20], or Nextflow [21]. Those frameworks have strong features to enable the efficient job execution that the current cwltool is not supporting, such as the job execution on a parallel computing platform. For example, all those workflow runners can submit a job over the job queuing system such as Univa Grid Engine [22], one of the most popular batch job queuing system. This allows a

parallel execution of a workflow, or enables to parallelize a calculation in a step of the workflow, which results in a better performance in terms of the processing duration. To parallelize a workflow or a step of workflow is the common practice in the bioinformatics data analysis.

However, we chose CWL as the workflow description framework and its reference implementation *cwltool* as the workflow runner for the system because CWL provide a way to share the workflow across different workflow systems. Once users collected the runtime metrics of workflows with CWL-metrics, they can execute the same workflow description with multiple workflow runner implementations. There are fifteen implementations listed as those supporting CWL [23]. While some implementations including Galaxy are currently not covering full functionality to import and export CWL workflows, others including Arvados, Toil, and Apache Airflow are already available to users. If one wanted to use a workflow system that does not support CWL yet, the summary of runtime metrics collected through Docker containers is still valuable information with different frameworks that execute command line tools in a similar fashion.

Yet we consider that the CWL-metrics has possibility to support more common use cases in bioinformatics and other data science applications. First, we aim to add a feature to collect metrics of parallelized job execution by CWL-metrics. We also expect there will be more workflows that need to use different container technologies such as Singularity [24], which currently the CWL-metrics is not able to support metrics collection. The future implementation of the CWL-metrics will cover those different runtime for more usability. We also aim to improve the implementation as it can provide other metrics related to the cost of cloud usage other than the instance running time, such as total data transfer size or total disk usage of the workflow run, which users need to mind to estimate the cost of cloud infrastructure.

The Common Workflow Language project has a subproject, CWLProv, to provide provenance information of workflow executions to improve reproducibility of workflows by tracking intermediate files and logs [25]. The provenance information helps users to track inputs and outputs of workflow runs by using file checksums but does not record the detail of the resource usage. Adding runtime metrics data to the provenance information will cover the information regarding deployment, which helps users to reproduce the runs

on a appropriate computing environment. Thus, the summary of runtime metrics collected by CWL-metrics should be bundled with the provenance information.

It is essential for researchers to have a flexible computing environment that can quickly scale out according to the amount of data. The fast deployment of the data analysis environment to an appropriate cloud instance supported by Docker, CWL, and CWL-metrics is a way to achieve the computational scale out, which brings a huge benefit for bioinformatics researchers.

Potential Implications

The Common Workflow Language project aims to support workflow description specification for all domains that work with data analysis pipelines. Therefore, CWL-metrics can contribute to other domains through the application of CWL. Sharing CWL workflows with the metrics data captured by CWL-metrics can help users to deploy them on an appropriate environment.

Methods

CWL-metrics software components

CWL-metrics runtime metrics capturing system is composed of five software components: Telegraf [26], Fluentd [27], Elasticsearch [28], Kibana [29], and a Perl daemon script. Telegraf is an agent to collect runtime metrics of running containers via Docker API using Telegraf Docker plugin. Fluentd works as a log data collector to send metrics data produced by Telegraf to the Elasticsearch server. Elasticsearch acts as a data store to accumulate runtime metrics data and workflow metadata, accepting JSON format data via API endpoint. Kibana is a data browsing dashboard for Elasticsearch to view raw JSON data and to summarize and visualize data [30]. Telegraf, Fluentd, Elasticsearch/Kibana launch as a set of containers during the initialization of CWL-metrics. CWL-metrics runs a Perl script which monitors processes on the host machine to track cwltool processes. To send execution log to the system, users need to run cwltool with the specified option to output the cwltool log to a file. Once the script found a cwltool process, the script runs a function to collect workflow information via debug output of the cwltool process recorded in a file, "docker info" command output, Docker container log via "docker ps" command, and output of system commands to collect environment information. CWL-metrics collect the running time of workflow steps via the Docker container log, thus the duration equals the time of the life of containers, which does not include the time of invocation by the workflow runner. CWL-metrics

provides a command *cwl-metrics*, which allows users to start and stop the metrics collection system, and fetch summarized runtime metrics data in a specified format, JSON or tab-separated format. The script to launch the whole system, CWL-metrics installation instructions, and the documentation are available on GitHub pages [31].

Packaging RNA-Seq tools and workflows

We created seven different RNA-Seq quantification workflows to capture runtime metrics and analyze performance on cloud infrastructure. Each workflow starts with a tool to download sequence data from Sequence Read Archive (SRA) [32], then convert the SRA-formatted file to FASTQ format. Consequently, each pipeline does sequence alignment to the reference genome sequence (HISAT2 [11], STAR [10], and TopHat2 [12]), quasi-mapping (Salmon), or pseudo-alignment (Kallisto [14]) to the set of reference transcript sequence, then perform transcript quantification. Most of the tool containers used in the workflows are from the Biocontainers [33] registry. We containerized the those tools were not available in the registry and uploaded them to the container registry service Quay [34, 35]. We described tool definitions such as input and output of tool execution and the workflow procedures in CWL tool files, which are available on GitHub [36]. Each

workflow has two options for sequence read layout, single-end and paired-end; thus we created 14 workflow variants in total. The Supplementary Table 1 shows the tool versions, the online location of the CWL tool files, and the original tool website locations.

Select RNA-Seq workflow input sequence data from the public data repository

To analyze the effect of sequence data quality to workflow runtime performance, we chose 9 samples of different read length and number of reads from the public raw sequencing data repository, SRA (Table 2). We used the Quanto database [37] to select the data by filtering length and number of sequence reads, with the condition of read length, 50, 75, or 100 and the approximate number of sequence, 1,000,000, 5,000,000, or 10,000,000. We filtered the data with the query of string matches with "organism == Homo sapiens", "study type == RNA-Seq", "read layout == PAIRED", and "instrument model == Illumina HiSeq", then manually picked data with sufficient amount of description from the corresponding results. Both single-end and paired-end workflows used the same dataset although single-end workflows treated paired-end read files reads as two single-end read files. The version of the reference genome is GRCh38 [38]. We downloaded the reference genome

file from the UCSC genome browser [39], and the Gencode gene annotation file version 28 from Gencode website [40].

Run workflows on AWS EC2

To evaluate the performance on running different RNA-Seq workflows, we selected instance types of two different sizes 2xlarge and 4xlarge from three categories, general purpose, compute optimized, and memory optimized to run all workflows for all samples (Table 3). Each combination of instance type, workflow, and sample data was executed for five times while CWL-metrics is running on the same machine to capture the runtime metrics information. All workflow runs used Elastic Block Storage of General Purpose SSD volumes as file storage. We downloaded all the reference data used for workflows in advance. The source of the used reference data, scripts to run workflows are available online [36].

Collect runtime metrics and summarize

After the workflow executions, we collected summarized metrics data from Elasticsearch with the *cwl-metrics fetch* command. Exported JSON format data were parsed by a Ruby script to create data summarized per workflow run, loaded in a Jupyter notebook [41] for further analysis. We calculated median

values of metrics for replications by R language functions [42], and we created the box plots by the ggplot2 package [43]. The notebook file is available on GitHub [44].

Figure legends

Figure 1: The container runtime metrics collection procedure with CWL-metrics

CWL-metrics was designed to capture runtime metrics of workflow steps automatically. After the initialization of the system, users only need to run a workflow *cwltool* to start capturing metrics. The system collects runtime metrics of containers, then the workflow metadata is captured after the workflow process finished. To retrieve runtime metrics, the *cwl-metrics* command can output summary data in JSON or tab-delimited format.

Figure 2: The CWL-metrics components and working process

CWL-metrics consists of a daemon process and several Docker containers on the host machine. The process and containers keep running until the system is terminated. Once a *cwltool* process starts running on the same machine, the CWL-metrics system monitors the process to get the list of workflow step

containers and log files. Every sixty seconds, the Telegraf container try to access the Docker daemon to get runtime metrics of running containers. The Fluentd container sends runtime metrics data collected by Telegraf to the Elasticsearch container. The CWL-metrics daemon process captures the cwltool log file and sends workflow metadata to Elasticsearch.

Figure 3: An example of runtime metrics data summarized by CWL-metrics

CWL-metrics can output JSON formatted data which includes workflow metadata, tool container metadata, and tool container runtime metrics. The workflow metadata appears once for one workflow run with data of multiple steps in "*steps*" key; this example only shows one step in the workflow for brevity. Each step has a name, exit status, input files with file size, and details of the Docker container. Runtime metric values may be null for short-time steps since CWL-metrics collects these metrics with sixty seconds interval.

Figure 4: Box plot of per sample processing duration distribution of HISAT2-StringTie workflow

We plotted the values of processing duration of workflow runs excluding data download time. The x-axis shows SRA Run ID of samples used as input data

with read length and number of reads. The y-axis shows the workflow processing duration in seconds. Values are separated and colored by the used instance type. Some runs on specific instance types are not in the plots because the failed executions are excluded. Each combination of sample and instance type were iterated 5 times to show the distribution of metrics. The plot shows that read length and the number of reads are both factors that affect the processing duration, and the differences between instance types are relatively small for smaller number of reads (1G bases), while instances with more CPU cores (*.4xlarge) show shorter processing duration for 10G base pair reads.

Figure 5: Box plot of processing duration and maximum memory usage of sample SRR2567462 per workflow

The values of processing duration exclude data download time. Both plots used values of workflow executions as single end input of SRR2567462. The x-axis shows workflow names, and the y-axis shows the processing duration in seconds and total memory usage in bytes. We iterated each combination of workflow and instance type for 5 times. The plot of processing duration shows that there is a significant difference in execution time between the TopHat2 workflow and the others. While the difference of processing durations is relatively small, workflows with STAR aligner require four or five times much

memory than HISAT2 workflows. These data suggest users should know about runtime metrics of workflows before selecting cloud instance type.

Supplementary Figure 1: Box plot of processing duration for all workflows

The x-axis shows *SRA Run ID* of input data with the read length and the number of reads. The y-axis shows the processing duration in seconds excluding data downloading time. In most of the used workflows, the read length and the number of reads of input data affect the processing time. Workflows with *STAR aligner* requires a large amount of memory; thus the executions on instance types with a smaller amount of memory have failed.

Supplementary Figure 2: Box plot of max memory usage for all workflows

The x-axis shows SRA Run ID of input data with the read length and the number of reads. The y-axis shows the maximum amount of memory used during the process in bytes. The distributions of values are large especially on runs which finishes in a short time, probably because 60 seconds interval of metrics capturing could not get consistent values.

Table legends

Table 1: The components of RNA-Seq quantification workflows

We described 7 different RNA-Seq quantification workflows in CWL. Each workflow description has two different options for read layout, single-end and paired-end. We selected two major read mapping tools STAR and Hisat2, with two transcriptome assemble and read count programs Cufflinks and StringTie. We also used two popular tools with different quantification approach, Kallisto and Salmon. We added TopHat2, one of the most popular programs for comparison purpose.

Table 2: The read characteristics of processed RNA-Seq data

We chose 9 different RNA-Seq data from the SRA, a public high-throughput sequencing repository. For performance comparison, each sequence was selected to be different in their read length and total number of reads. All data are from human samples sequenced by the Illumina HiSeq platform.

Table 3: The machine specs of AWS EC2 instance types used in the metrics collection

To compare the performance of workflow runs on different computing platforms, we selected 3 categories from AWS EC2 categories, general purpose, compute optimized, and memory optimized. We further selected two different instance types from those three categories according to the number of virtual

CPUs, 2xlarge and 4xlarge, with 8 and 16 CPU cores, respectively. Instance usage prices are as of 14 August 2018 for on-demand use in the US East (N. Virginia) region. Prices are not including charges for storage, network usage, and other AWS features.

Table 4: The runtime metrics comparison of TopHat2 and HISAT2

We summarized the runtime metrics values to compare two different workflows HISAT2-cufflinks and TopHat2-cufflinks. All runs used input data SRR2567462. The read length was 100bp, the number of reads was 10,007,044.00, and the read layout was single-end. The shown values are workflow duration in seconds, the maximum CPU usage in percentage, the total amount of memory in bytes, the total amount of cache in bytes, the total amount of block IO in bytes, and the cost per run in USD. We calculated the median values for metrics values from the data of 5 times workflow iteration. Values can be zero for short-time steps since CWL-metrics collects these metrics with 60 seconds interval.

Supplementary Table 1: The versions and containers of tools used in the RNA-Seq workflows

We used 11 tools in total to construct 7 RNA-Seq quantification workflows. The two tools we developed, *download-sra* [35] and *pfastq-dump* [36], are packaged in containers by ourselves. The container of Salmon was available on its developer's build. We found the rest of tools in Biocontainers registry. We wrapped all the tools as CWL CommandLineTool class files and available on GitHub.

Availability of source code and requirements

For CWL-metrics, the runtime metrics capturing system:

Project name: CWL-metrics

Project home page: https://inutano.github.io/cwl-metrics/

DOI: 10.5281/zenodo.2583319

Operating system(s): Platform independent

Programming language: Perl v5.18.2 or higher

Other requirements: Docker 18.06.0-ce or higher and Docker Compose 1.22.0 or higher, cwltool 1.0.20180820141117 or higher

License: MIT

Any restrictions to use by non-academics: NA

For the scripts and the notebook for visualization on this manuscript:

Project name: cwl-metrics-manuscript

Project home page: https://github.com/inutano/cwl-metrics-manuscript

DOI: 10.5281/zenodo.2583314

Operating system(s): Platform independent

Programming language: Ruby 2.5.1 or higher

Other requirements: Docker 18.06.0-ce or higher

License: MIT

Any restrictions to use by non-academics: NA

For the CWL definitions for tools and workflows used for benchmark:

Project name: Pitagora CWL

Project home page: https://github.com/pitagora-network/pitagora-cwl

DOI: 10.5281/zenodo.2583023

Operating system(s): Platform independent

Programming language: Common Workflow Language v1.0

Other requirements: NA

License: Apache 2.0

Any restrictions to use by non-academics: NA

For the SRA data download tool:

Project name: download-sra

Project home page: https://github.com/inutano/download-sra

DOI: 10.5281/zenodo.2590835

Operating system(s): Platform independent

Programming language: Shell

Other requirements: wget, curl

License: MIT

Any restrictions to use by non-academics: NA

For the SRA-formatted data parallel decompress tool:

Project name: pfastq-dump

Project home page: https://github.com/inutano/pfastq-dump

DOI: 10.5281/zenodo.2590841

Operating system(s): Platform independent

Programming language: Shell

Other requirements: SRA toolkit

License: MIT

Any restrictions to use by non-academics: NA

Availability of supporting data and materials

The source code and documentation for CWL-metrics system is available on GitHub [45]. The workflows and the scripts used for benchmark experiment was published on GitHub [36]. The reference data used for workflow execution are available on Zenodo [46]. The intermediate and output files are also provided on Zenodo [47]. The data set used for the visualizations of this article is available in figshare [48, 49]. The full summary data and visualization on Jupyter notebook is available on GitHub [44] and nbviewer [50].

Declarations

List of abbreviations

AWS: Amazon Web Service,

bp: base pairs

CWL: Common Workflow Language

EC2: Elastic Compute Cloud

IO: input/output

SRA: Sequence Read Archive

TSV: tab separated values

Competing interests

The authors declare that they have no competing interests.

Funding

This work has been supported by CREST, Japan Science and Technology Agency (JST), JPMJCR1501.

Authors' contributions

Conceptualization, Methodology, Software, Investigation: TO TT. Visualization, Writing original draft: TO. Supervision: OO.

Acknowledgements

The authors are grateful to Prof. Kento Aida and the Inter-Cloud CREST team for constructive comments and discussions. The authors also thank the open source communities: Pitagora Galaxy, Galaxy Project, Common Workflow Language, Bioinformatics Open Source Conference, and the BioHackathon for

many comments and suggestions. We performed implementation and testing of the system on the NIG supercomputer at ROIS National Institute of Genetics.

References

1. Chang J. Core services: Reward bioinformaticians. Nature 2015;520:151–2. DOI: 10.1038/520151a
2. Prins P, de Ligt J, Tarasov A et al. Toward effective software solutions for big biology. Nature Biotechnology 2015;33:686–7. DOI: 10.1038/nbt.3240
3. Merkel D. Docker: lightweight Linux containers for consistent development and deployment. Linux Journal. 2014 Mar 1;2014(239):2. ISSN: 1075-3583
4. Di Tommaso P, Palumbo E, Chatzou M et al. The impact of Docker containers on the performance of genomic pipelines. PeerJ 2015;3:e1273. DOI: 10.7717/peerj.1273

5. Afgan E, Baker D, Batut B et al. The Galaxy platform for accessible, reproducible and collaborative biomedical analyses: 2018 update. Nucleic Acids Research 2018;46:W537–44. DOI: 10.1093/nar/gky379

6. Amstutz P, Crusoe MR, Nebojša Tijanić et al. Common Workflow Language, v1.0. 2016. DOI: 10.6084/m9.figshare.3115156.v2.

7. Stein LD. The case for cloud computing in genome informatics. Genome Biology 2010;11:207. DOI: 10.1186/gb-2010-11-5-207

8. Amazon EC2 Instance Types https://aws.amazon.com/ec2/instance-types/ Accessed 11 Mar. 2018.

9. common-workflow-language/cwltool https://github.com/common-workflow-language/cwltool Accessed 11 Mar. 2018.

10. Dobin A, Davis CA, Schlesinger F, Drenkow J, Zaleski C, Jha S et al. STAR: ultrafast universal RNA-seq aligner. Bioinformatics 2012; 29: 15–21. DOI: 10.1093/bioinformatics/bts635

11. Kim D, Langmead B, Salzberg SL. HISAT: a fast spliced aligner with low memory requirements. Nature Methods 2015; 12: 357–360. DOI: 10.1038/nmeth.3317

12. Trapnell C, Roberts A, Goff L, Pertea G, Kim D, Kelley DR et al. Differential gene and transcript expression analysis of RNA-seq experiments with TopHat and Cufflinks. Nature Protocols 2012; 7: 562–578. DOI: 10.1038/nprot.2012.016

13. Pertea M, Pertea GM, Antonescu CM, Chang T-C, Mendell JT, Salzberg SL. StringTie enables improved reconstruction of a transcriptome from RNA-seq reads. Nature Biotechnology 2015; 33: 290–295. DOI: 10.1038/nbt.3122

14. Bray NL, Pimentel H, Melsted P, Pachter L. Near-optimal probabilistic RNA-seq quantification. Nature Biotechnology 2016; 34: 525–527. DOI: 10.1038/nbt.3519

15. Patro R, Duggal G, Love MI, Irizarry RA, Kingsford C. Salmon provides fast and bias-aware quantification of transcript expression. Nature Methods 2017; 14: 417–419. DOI: 10.1038/nmeth.4197

16. TopHat: A spliced read mapper for RNA-Seq. https://ccb.jhu.edu/software/tophat/index.shtml Accessed 11 Mar. 2018.

17. Runtime metrics | Docker Documentation https://docs.docker.com/config/containers/runmetrics/#network-metrics Accessed 11 Mar. 2018.

18. Tyryshkina A, Coraor N, Nekrutenko A. Predicting runtimes of bioinformatics tools based on historical data: Five years of Galaxy usage. Bioinformatics. 2019. DOI: 10.1093/bioinformatics/btz054

19. Vivian J, Rao AA, Nothaft FA, Ketchum C, Armstrong J, Novak A, Pfeil J, Narkizian J, Deran AD, Musselman-Brown A, Schmidt H. Toil enables reproducible, open source, big biomedical data analyses. Nature biotechnology. 2017 Apr 11;35(4):314. http://doi.org/10.1038/nbt.3772

20. Voss K, Van der Auwera G and Gentry J. Full-stack genomics pipelining with GATK4 + WDL + Cromwell. 2017. F1000Research, 6(ISCB Comm J):1381. DOI: 10.7490/f1000research.1114634.1

21. Di Tommaso P, Chatzou M, Floden EW et al. Nextflow enables reproducible computational workflows. Nature Biotechnology 2017;35:316–9. DOI: 10.1038/nbt.3820

22. Univa Grid Engine http://www.univa.com/products/ Accessed on 30 Oct 2018.

23. Common Workflow Language https://www.commonwl.org/ Accessed on 30 Oct 2018.

24. Kurtzer GM, Sochat V, Bauer MW. Singularity: Scientific containers for mobility of compute. 2017. PloS one, 12(5), e0177459. DOI: 10.1371/journal.pone.0177459

25. Khan FZ, Soiland-Reyes S, Sinnott RO et al. CWLProv: Interoperable Retrospective Provenance Capture And Computational Analysis Sharing. 2018. DOI: 10.5281/zenodo.1966881

26. Telegraf https://www.influxdata.com/time-series-platform/telegraf/ Accessed 11 Mar. 2018.

27. Fluentd https://www.fluentd.org/ Accessed 11 Mar. 2018.

28. Elasticsearch https://www.elastic.co/products/elasticsearch Accessed 11 Mar. 2018.

29. Kibana https://www.elastic.co/products/kibana Accessed 11 Mar. 2018.

30. Kibana User Guide https://www.elastic.co/guide/en/kibana/current/index.html Accessed 11 Mar. 2018.

31. CWL-metrics https://inutano.github.io/cwl-metrics/ Accessed 11 Mar. 2018.

32. Kodama Y, Shumway M, Leinonen R. The sequence read archive: explosive growth of sequencing data. Nucleic Acids Research 2011; 40: D54–D56. DOI: 10.1093/nar/gkr854

33.

34. da Veiga Leprevost F, Grüning BA, Alves Aflitos S et al. BioContainers: an open-source and community-driven framework for software standardization. Valencia A (ed.). Bioinformatics 2017;33:2580–2.

35. Tazro Inutano Ohta. inutano/download-sra: v0.1.3. 2019. DOI:10.5281/zenodo.2590835

36. Tazro Inutano Ohta. inutano/download-sra: v0.1.3. 2019. DOI:10.5281/zenodo.2590835.

37. Tazro Inutano Ohta, yyabuki, Tomoya Tanjo, & Michael R. Crusoe. (2019, March 4). pitagora-network/pitagora-cwl: v0.1.0 (Version v0.1.0). Zenodo. http://doi.org/10.5281/zenodo.2583024

38. Ohta T, Nakazato T, Bono H. Calculating the quality of public high-throughput sequencing data to obtain a suitable subset for reanalysis from the Sequence Read Archive. GigaScience 2017;6. DOI: 10.1093/gigascience/gix029

39. Schneider VA, Graves-Lindsay T, Howe K, Bouk N, Chen H-C, Kitts PA et al. Evaluation of GRCh38 and de novo haploid genome assemblies demonstrates the enduring quality of the reference assembly. Genome Research 2017; 27: 849–864. DOI: 10.1101/gr.213611.116

40. Casper J, Zweig AS, Villarreal C, Tyner C, Speir ML, Rosenbloom KR, Raney BJ, Lee CM, Lee BT, Karolchik D, Hinrichs AS. The UCSC genome browser database: 2018 update. Nucleic Acids Research. 2017;46(D1):D762-9. DOI: 10.1093/nar/gkx1020

41. Harrow J, Frankish A, Gonzalez JM et al. GENCODE: The reference human genome annotation for The ENCODE Project. Genome Research 2012;22:1760–74. DOI: 10.1101/gr.135350.111

42. Kluyver Thomas, Ragan-Kelley Benjamin, Pérez Fernando, Granger Brian, Bussonnier Matthias, Frederic Jonathan et al. Jupyter Notebooks - a publishing format for reproducible computational workflows. STAL 2016; 0: 87–90. DOI: 10.3233/978-1-61499-649-1-87

43. R Core Team (2018). R: A language and environment for statistical computing. R Foundation for Statistical Computing, Vienna, Austria. URL https://www.R-project.org/. Accessed 11 Mar. 2018.

44. Wickham H. ggplot2: Elegant Graphics for Data Analysis. Springer-Verlag New York; 2009. DOI: 10.1007/978-0-387-98141-3

45. Ohta T. inutano/cwl-metrics-manuscript: v0.1.0 (Version v0.1.0). 2019. Zenodo. DOI: 10.5281/zenodo.2583315

46. Ohta T, Tanjo T. inutano/cwl-metrics: v0.1.0 (Version v0.1.0). 2019-03-05. Zenodo. DOI: 10.5281/zenodo.2583320

47. Ohta, T. The reference index files used for RNA-seq workflow benchmark in CWL-metrics manuscript [Data set]. 2019. Zenodo. DOI: 10.5281/zenodo.2587202

48. Ohta, T. The output and the log files from RNA-Seq workflow benchmark for CWL-metrics manuscript [Data set]. 2019. Zenodo. DOI: 10.5281/zenodo.2586547

49. Ohta, T. Runtime metrics data of 7 different RNA-Seq quantification workflows. figshare. 2018-10-18 DOI: 10.6084/m9.figshare.7222775.v1

50. Ohta, T. The supplemental information for RNA-Seq workflow benchmark by CWL-metrics. figshare. 2019-03-05. DOI: 10.6084/m9.figshare.7801046.v1

51. CWL-metrics: workflow runtime metrics analysis https://nbviewer.jupyter.org/github/inutano/cwl-metrics-manuscript/blob/master/notebook/CWL-metrics%20runtime%20metrics%20analysis.ipynb Accessed 11 Mar. 2018.

 Click here to access/download;Table;file-6.pdf

Table 1: The components of RNA-Seq quantification workflows

Workflow name	Steps	CWL definition files
tophat2-cufflinks	download-sra, pfastq-dump, tophat2-mapping, cufflinks	https://github.com/pitagora-galaxy/cwl/tree/master/workflows/tophat2-cufflinks
hisat2-cufflinks	download-sra, pfastq-dump, hisat2-mapping, samtools_sam2bam, samtools_sort, cufflinks	https://github.com/pitagora-galaxy/cwl/tree/master/workflows/hisat2-cufflinks
hisat2-stringtie	download-sra, pfastq-dump, hisat2-mapping, samtools_sam2bam, samtools_sort, stringtie	https://github.com/pitagora-galaxy/cwl/tree/master/workflows/hisat2-stringtie
star-cufflinks	download-sra, pfastq-dump, star-mapping, samtools_sam2bam, samtools_sort, cufflinks	https://github.com/pitagora-galaxy/cwl/tree/master/workflows/star-cufflinks
star-stringtie	download-sra, pfastq-dump, star-mapping, samtools_sam2bam, samtools_sort, stringtie	https://github.com/pitagora-galaxy/cwl/tree/master/workflows/star-stringtie
kallisto	download-sra, pfastq-dump, kallisto-quant	https://github.com/pitagora-galaxy/cwl/tree/master/workflows/kallisto
salmon	download-sra, pfastq-dump, salmon-quant	https://github.com/pitagora-galaxy/cwl/tree/master/workflows/salmon

Click here to access/download;Table;file-7.pdf

Table 2: The read characteristics of processed RNA-Seq data

SRA Run ID	Read length	Number of reads per strand	BioSample ID	Sample description	Sequencing instrument
SRR4250750	50	1,000,425.00	SAMN05779985	cultured embryonic stem cells	Illumina HiSeq 2500
SRR5185518	50	5,008,398.00	SAMN06239034	cultured embryonic stem cells	Illumina HiSeq 2500
SRR2932901	50	10,017,495.00	SAMN04211783	fetal lung fibroblasts	Illumina HiSeq 2500
SRR4428678	75	1,043,870.00	SAMN05913930	embryonic stem cell derived macrophage	Illumina HiSeq 4000
SRR4241930	75	5,004,985.00	SAMN05770731	PGC-like cells (PGCLCs)	Illumina HiSeq 2000
ERR204893	75	10,234,883.00	SAMEA1573291	lymphoblastoid cell line	Illumina HiSeq 2000
SRR5168756	100	1,006,868.00	SAMN06218220	subcutaneous metastasis	Illumina HiSeq 2500
SRR5023408	100	5,004,554.00	SAMN06017954	primary breast cancer	Illumina HiSeq 2500
SRR2567462	100	10,007,044.00	SAMN04147557	prostate cancer cells LNCaP	Illumina HiSeq 2500

 Click here to access/download;Table;file-8.pdf

Table 3: The machine specs of AWS EC2 instance types used in the metrics collection

Instance type	Category	vCPU	ECU	Memory (GiB)	Linux/UNIX Usage (per Hour)
m5.2xlarge	General Purpose	8	31	32	$0.384
m5.4xlarge	General Purpose	16	60	64	$0.768
c5.2xlarge	Compute Optimized	8	34	16	$0.34
c5.4xlarge	Compute Optimized	16	68	32	$0.68
r5.2xlarge	Memory Optimized	8	31	64	$0.504
r5.4xlarge	Memory Optimized	16	60	128	$1.008

Figure 4

Click here to access/download;Table;file-9.pdf

Table 4: The runtime metrics comparison of TopHat2 and HISAT2

Workflow name	Instance type	Workflow duration	Max CPU usage	Total amount of memory	Total amount of memory cache	Total amount of BlockIO	Cost per run
HISAT2-Cufflinks	c5.2xlarge	1014.5	796.8330796	10033995776	5183479808	4748816384	0.0958
HISAT2-Cufflinks	c5.4xlarge	778	1595.031529	9163902976	4314202112	1204879360	0.147
HISAT2-Cufflinks	m5.2xlarge	1013	799.0908131	11254398976	6396575744	1204858880	0.1081
HISAT2-Cufflinks	m5.4xlarge	846	1538.403444	11802640384	6938824704	331776	0.1805
HISAT2-Cufflinks	r5.2xlarge	1015	798.2115564	10912165888	6065545216	3608539136	0.1421
HISAT2-Cufflinks	r5.4xlarge	834	1588.403182	9973350400	5116166144	0	0.2335
TopHat2-Cufflinks	c5.2xlarge	5139	797.8534259	12310124544	8869050368	12343222272	0.4854
TopHat2-Cufflinks	c5.4xlarge	3695	1587.471528	15879102464	7833452544	1204891648	0.6979
TopHat2-Cufflinks	m5.2xlarge	5579	799.5529991	15149662208	9395200000	51970048	0.5951
TopHat2-Cufflinks	m5.4xlarge	3981	1595.226713	15875092480	7913992192	49848320	0.8493
TopHat2-Cufflinks	r5.2xlarge	5487	798.6095883	15152807936	9492783104	49848320	0.7682
TopHat2-Cufflinks	r5.4xlarge	4001	1291.353527	15877746688	7930822656	49848320	1.1203

Figure 1

Click here to access/download;Figure;file-1.pdf

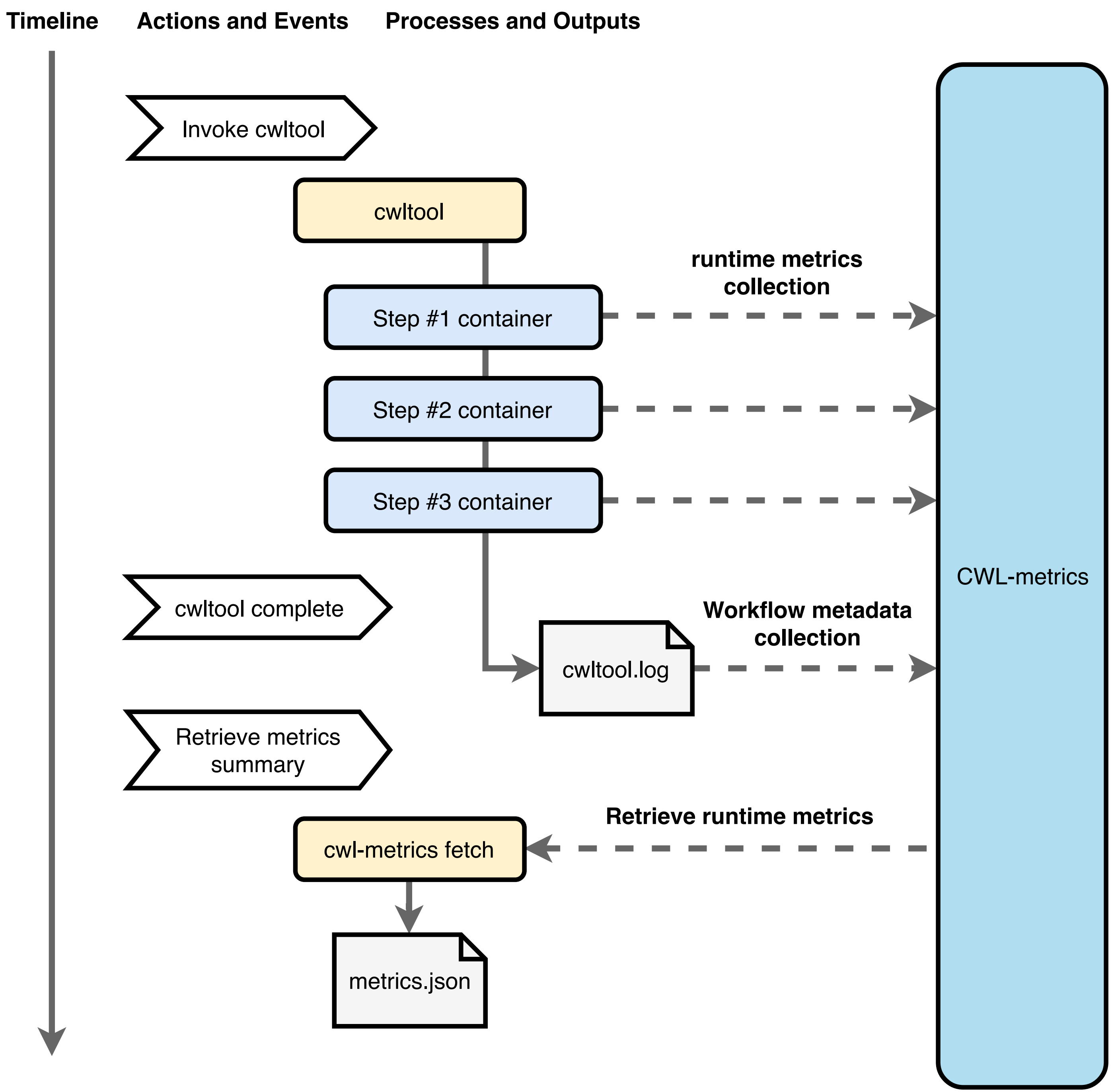

Figure 3 Click here to access/download;Figure;file-3.pdf


```
{
  "CWL-metrics": [
    {
      "workflow_id": "3b66284a-969d-11e8-8d0f-0ae229374f7a",
      "workflow_name": "hisat2-cufflinks_wf_pe.cwl",
      "workflow_start_date": "2018-08-02T21:41:43+00:00",
      "workflow_end_date": "2018-08-02T21:44:25+00:00",
      "workflow_elapsed_sec": 162,
      "platform": {
        "instance_type": "c5.4xlarge",
        "region": "us-east-1a",
        "hostname": "4138af0fad86",
        "total_memory": "31897692",
        "disk_size": "508187044"
      },
      "steps": {
        "fcc52b5d2d3bf6dc1106c83117f5956c968047cbf0c5642144b86dbee32da619": {
          "stepname": "hisat2_mapping",
          "tool_status": "success",
          "input_files": {
            "SRR4428678_1.fastq.gz": 43828265,
            "SRR4428678_2.fastq.gz": 53452040,
            "out.sam": 778641728
          },
          "docker_image": "quay.io/biocontainers/hisat2:2.1.0--py36h2d50403_1",
          "docker_cmd": "hisat2 -S /var/spool/cwl/out.sam -x /var/lib/cwl/stg94f48183-8e7c-4fcb-
bc4b-58b2a7d33240/hisat2_GRCh38/genome --downstream-transcriptome-assembly --dta-cufflinks -1
/var/lib/cwl/stge9112392-7277-4049-8410-25324f93ec7c/SRR4428678_1.fastq.gz -2
/var/lib/cwl/stg31076a0b-02ab-4de9-9e8b-3b9af44152f8/SRR4428678_2.fastq.gz --threads 16 --time",
          "docker_start_date": "2018-08-02T21:41:52+00:00",
          "docker_end_date": "2018-08-02T21:42:09+00:00",
          "docker_elapsed_sec": 17.517223481,
          "docker_exit_code": 0,
          "metrics": {
            "cpu_total_percent": 1571.87279333333,
            "memory_max_usage": 5096611840,
            "memory_cache": 309497856,
            "blkio_total_bytes": null
          }
        }
      }
    }
  ]
}
```

Figure 4

Click here to access/download;Figure;file-4.pdf

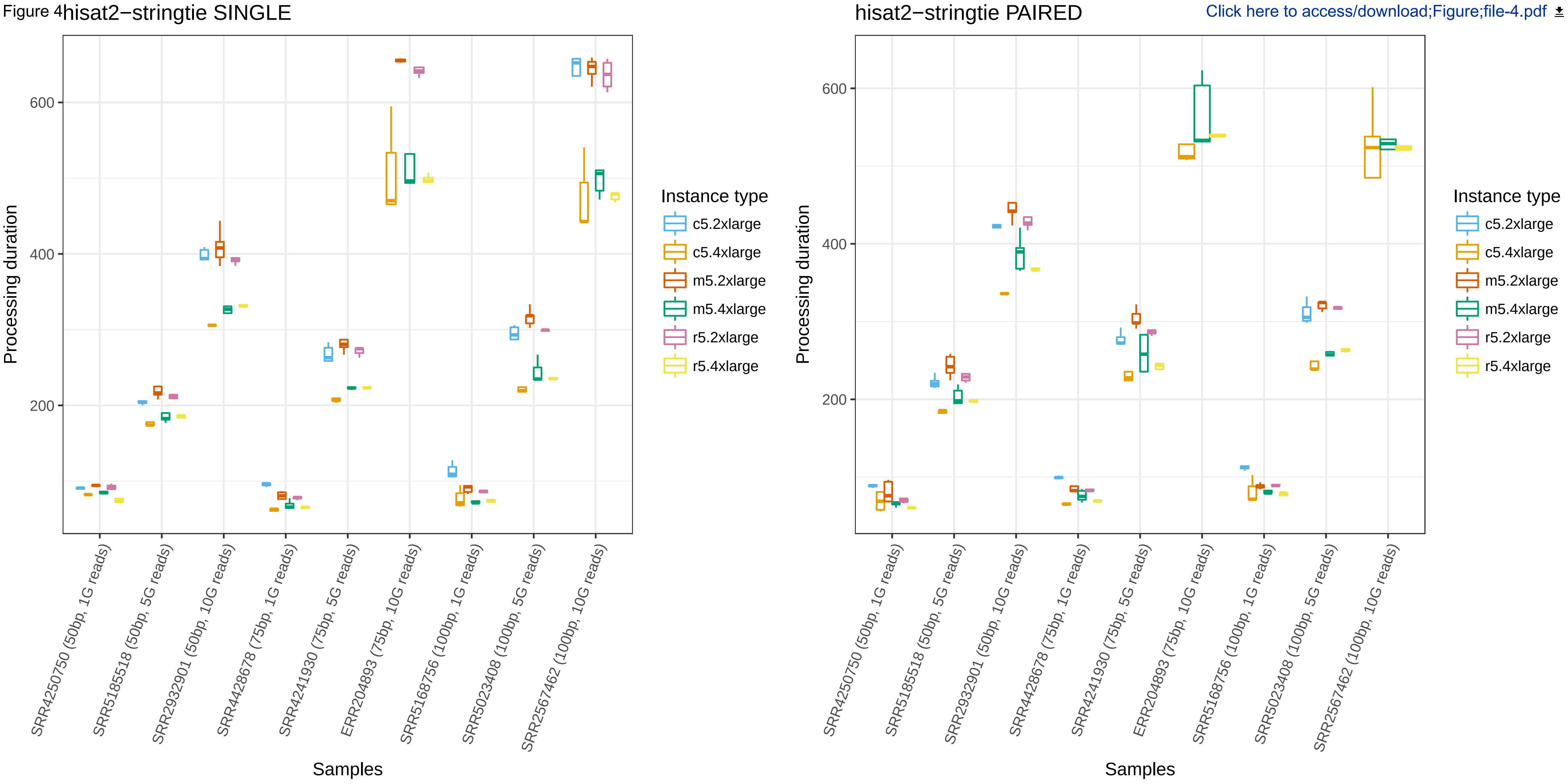

Figure 5

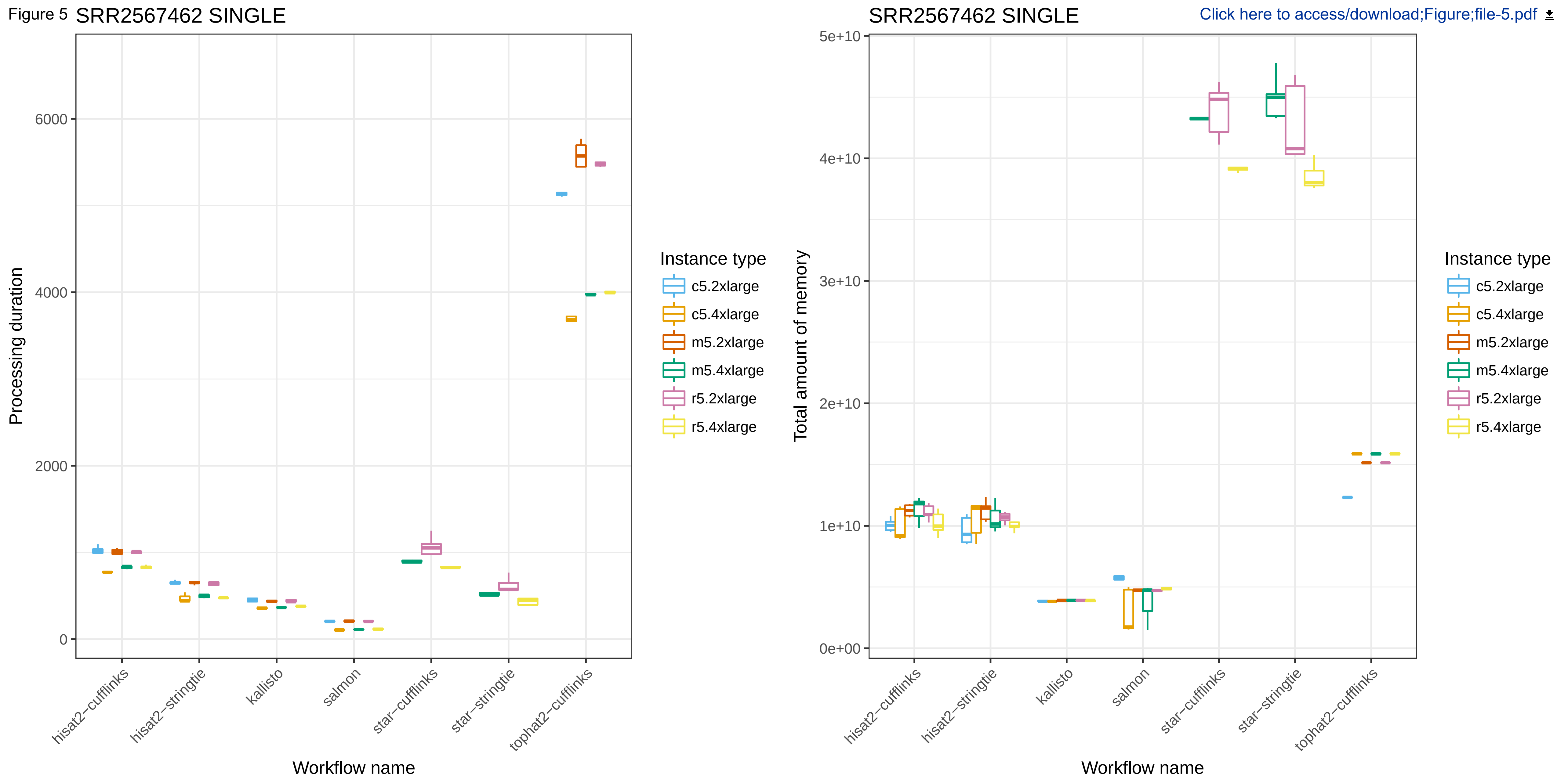

Click here to access/download;Figure;file-5.pdf

Figure 2

Click here to access/download;Figure;Figure2.pdf

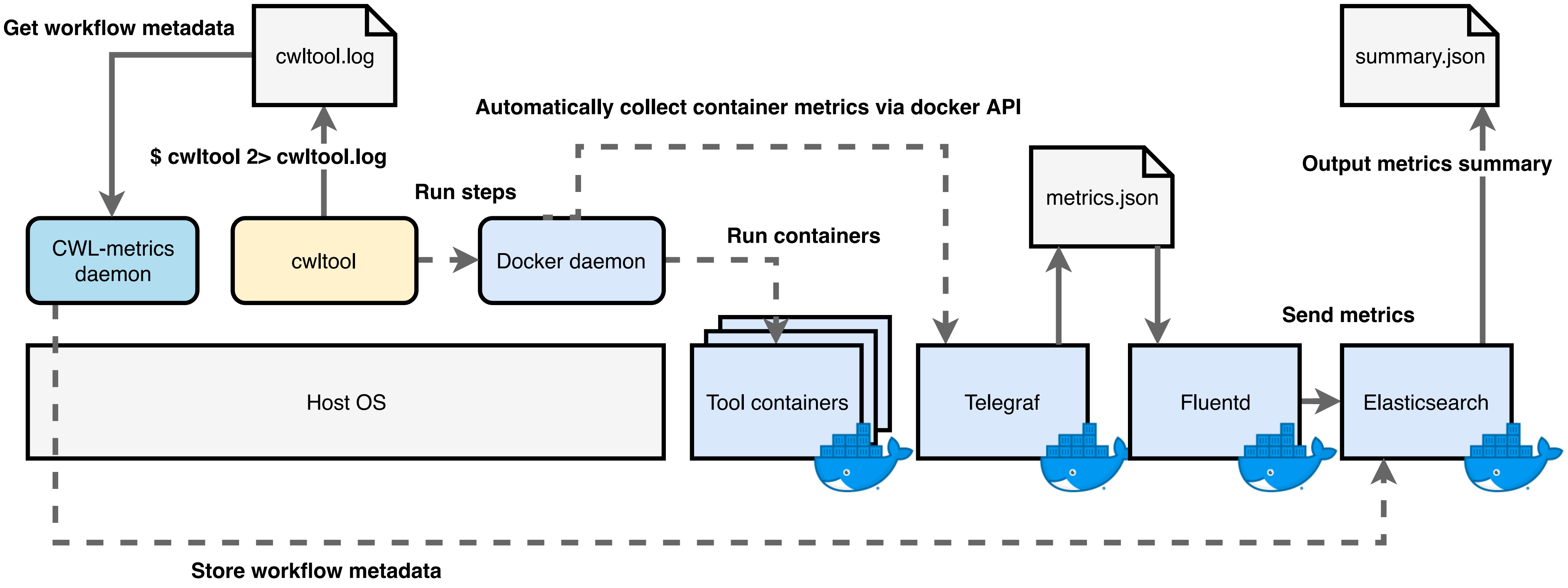

Supplemental file 1

Click here to access/download
Supplementary Material
supp-1.pdf

Click here to access/download
Supplementary Material
supp-2.pdf

Supplemental file 3

Click here to access/download
Supplementary Material
supp-3.pdf

response to reviewers Click here to access/download;Personal Cover;Response to reviewers.pdf

GIGA-D-18-00427
Accumulating computational resource usage of genomic data analysis workflow to optimize cloud computing instance selection
Tazro Ohta; Tomoya Tanjo; Osamu Ogasawara
GigaScience

We deeply thank the reviewers for their positive comments and suggestions. Those suggestions have greatly improved the quality of our project. Our responses are below in-line (the reviewer's comments are in italics).

Response to the reviewers' comments:

Reviewer #1:

Overall the authors provide a useful utility to cwltool that allows for an easy to use collection of runtime metrics. These metrics can be used to make informed decisions on estimating cost and which VM flavors are most efficient. My biggest concern is that the language throughout the manuscript except for a small part of the discussion conveys the idea that this tool is CWL-specific. CWL is simply the specification for defining a workflow and does not define anyway to report logs or runtime metrics which are defined by specific CWL engines. Thus, CWL-metrics is actually dependent on the cwltool reference implementation and the way it currently outputs logs. To me, CWL-metrics is more like an enhancement utility to the cwltool package than a part of CWL itself. I think this is an important distinction to make, especially since cwltool currently only handles serial operation of highly parallelizable workflows. So, if you were really concerned about cost, you likely wouldn't use cwltool as an engine, but look into something like Cromwell, Cavatica (Seven Bridges), or use a GA4GH TES implementation like Funnel. Again, this tool is indeed useful as I have personally used it, I just think that it is important to better clarify this in the manuscript.

Response: We agree with the reviewer's assessment. We started this project to develop a method to provide runtime metrics of CWL workflows so users can share workflows with their resource requirements. The current implementation of CWL-metrics, however, as the reviewer indicates, is the utility tool for cwltool. This is just because the cwltool is the reference implementation of CWL, and will be maintained with the updates of the specification. Yet we think we need to increase the coverage of workflow runners so users can get more practical information. We agree that it is important to make the current status clear. To emphasize the CWL-metrics depends on cwltool, we changed the sentence in the abstract to "We developed CWL-metrics, **a utility tool for cwltool**, the reference implementation of CWL, to collect runtime metrics of Docker containers and workflow metadata to analyze resource requirement of workflows" and added "**CWL-metrics works with cwltool, the reference implementation of CWL**" in the last paragraph of the background section.

Related to this, the authors mention Nextflow and Galaxy among others; however, they don't mention WDL or Cromwell which I believe is much more closely related to CWL and I think merit mentioning, especially since Cromwell can run CWL workflows at scale and in parallel.

Response: In the original manuscript, we mentioned Galaxy, Toil, and nextflow because they are the runners that can collect runtime information like CWL-metrics. We added a new paragraph in the discussion section to mention Cromwell and the limitation of cwltool in parallel job execution.

The following are minor things that I believe need to be addressed. The authors use the term "alignment-like" when referring to tools like Kallisto and Salmon; however, I believe the appropriate term is "pseudo-alignment".

Response: The Kallisto is using the term "pseudo-alignment" in its paper and documentation, but the Salmon does not. The authors of Salmon call its algorithm "quasi-mapping" and did not use the "pseudo-alignment" (https://doi.org/10.1038/nmeth.4197). Therefore we used the term "alignment-like", but it is still confusing. We changed the sentence to call them software use "different alignment approaches".

In figures 4 and 5, I assume the duration y-axis is in seconds; however, this doesn't seem to be mentioned in the description or axis labels and is important especially for people unfamiliar with the workflows.

Response: We added the corresponding units to the Y-axis labels of the plots. Thank you for pointing out the issue.

Finally, I don't feel like the authors provided any clear "future features" they would like to work on (or have the community contribute to). For example, they mention some limitations (e.g., scatter/gather across nodes) that could be overcome by having a centralized service (could be containerized) that all workers post metrics to.

Response: We added a new paragraph in the discussion section with the future prospects including the support of parallelized job execution and the different container runtime like Singularity.

Reviewer #2:

In this paper the authors describe a system to collect execution runtime metrics for computational workflows described using the Common Workflow Language notation. They also provide a benchmark of different tools executed against different dataset to show the benefits for their approach.

The topic is quite interesting because, given the exponential growth of genomics data, there's a pressing need to optimise bioinformatics tools and workflows for better resources allocations and usage in order to optimise the overall costs of long running in-silico data analyses.

The paper is easy to read, well structured and informative. It's particularly interesting the benchmark comparing different genome sequence aligners resource usage.

The only point to make is that the manuscript would capture the interest of a broader audience if the authors would provide a more balanced comparison with similar technologies such as Galaxy and Nextflow. For example the authors mention that their system only work for specific a CWL implementation (cwl-runner) and require the usage of Docker compliant system along with the deployment of other third party tools (eg. Telegraph, Elasticsearch, etc.) which installation could be challenging the average workflow users. Nextflow implements a very similar feature as the one describe in this manuscript to collect, visualise and export the execution metrics. However it can be used irrespective the execution platform supported by the tool (ie. local execution, clusters and clouds), in a single node or multi-node deployment and does not require the installation of any third party software components, either with or without containerised execution (disclaimer, the writer is the creator of the Nextflow tool).

Response: We added a paragraph in the discussion section for a more clear comparison of runners in terms of parallel job execution. The main difference between the cwltool and the other runners that can collect runtime metrics is the ability to capture the parallelized workflow job. We described the limitation of the current implementation of CWL-metrics that depends on cwltool. We want to note that the CWL-metrics users do not need to install Telegraph or Elasticsearch by themselves because the system automatically fetches those components as Docker containers. The prerequisites of the system are git, curl, Perl, Docker, and Docker compose. We added a sentence to mention this in the "Implementation of CWL-metrics" in the result section.

Very minor note, the usage of a human friendly format and data units for time and memory (e.g. seconds or Mega/Giga bytes) values would make the charts more readable.

Response: We added the corresponding units to the Y-axis labels of the plots. Thank you for your suggestion.

- Paolo

Response: Thank you very much, Paolo! Taz :)

Reviewer #3:

This manuscript proposes cwl-metrics, a way to collect metrics from Common Workflow Language executions of bioinformatics tools using Docker containers.

The manuscript is submitted as a Technical Note, but the research is of such a high quality that this could even be a Research article had it also provided a broader Background and a Discussion with comparison of workflow metrics systems beyond CWL.

Response: We submitted this manuscript via the direct submission system through bioRxiv, which did not give us an article type selection. We suppose that the GigaScience editorial office made a decision to make this a Technical Note, but we would be happy to change the category to Research article if possible.

The authors have been diligent in reproducibility and been good practitioners of Open Science, recording rich details of their evaluations and providing installation scripts for not just the software but also the evaluation setup. Some small issues remain to make this truly reproducible.

The English language of the manuscript is however not of a good quality for publication, to a degree where this can be confusing. Knowing the authors work from the CWL community I have provided feedback within the annotated manuscript (attached PDF) using ISO5776 text proof notation.

See the detailed review PDF for further comments according to GigaScience review guidelines, in particular the reproducibility section.

My detailed review is also web-accessible at the (secret) URL https://gist.github.com/stain/30e49363238d5a35e26f9fb1a31ebf8e

Response: Thank you very much for the many practical suggestions and corrections in detail. The correction of English writing was very helpful. We greatly appreciate your help.

Minor Revisions required:

** English Language needs to be revised*

Response: We modified the text following your suggestions and corrections.

** Missing DOIs for citations*

Response: We added the DOIs to the reference section.

** Missing DOIs for code/workflows, not (just) GitHub links*

Response: For our GitHub repositories, we assigned DOIs and used them in the reference section.

** License on CWL workflows/tools*

Response: We added the license to the repository and to the individual files (https://github.com/pitagora-network/pitagora-cwl). The license is Apache-2.0 following the best practice of the Common Workflow Language project.

** Fix Reproducibility issue in Notebook (missing files)*

Response: We uploaded the missing files to the Figshare and fixed the Notebook code to fix the issue. We also tested the Notebook correctly works on a new machine environment.

** License and source/upstream attribution for quay.io docker images*

Response: We added the GPL-3.0 license for the Docker images we uploaded to Quay.io.

Other suggestions in this review are recommended, but not required.

Response: We also fixed documentations on GitHub to provide more details for workflows and test scripts we provide. The documentation for reference data we used for the benchmark is also available on the new GitHub repo (https://github.com/pitagora-network/pitagora-cwl). We uploaded the reference data index to Zenodo (https://doi.org/10.5281/zenodo.2587201). We also uploaded the intermediate files and final outputs of the workflows executed for the benchmarking to Zenodo (https://doi.org/10.5281/zenodo.2586546). Thank you very much again for your suggestions, we are sure that the CWL-metrics project is now more reproducible.
