# Supplementary material for: Accumulating computational resource usage of genomic data analysis workflow to optimize cloud computing instance selection
Source: Gigascience. 2019 Apr 24;8(4):giz052. doi: 10.1093/gigascience/giz052 (PMC6479428; doi:10.1093/gigascience/giz052)
Supplement: Supplemental Files [file giz052_supplemental_files.zip › supp-1.pdf]

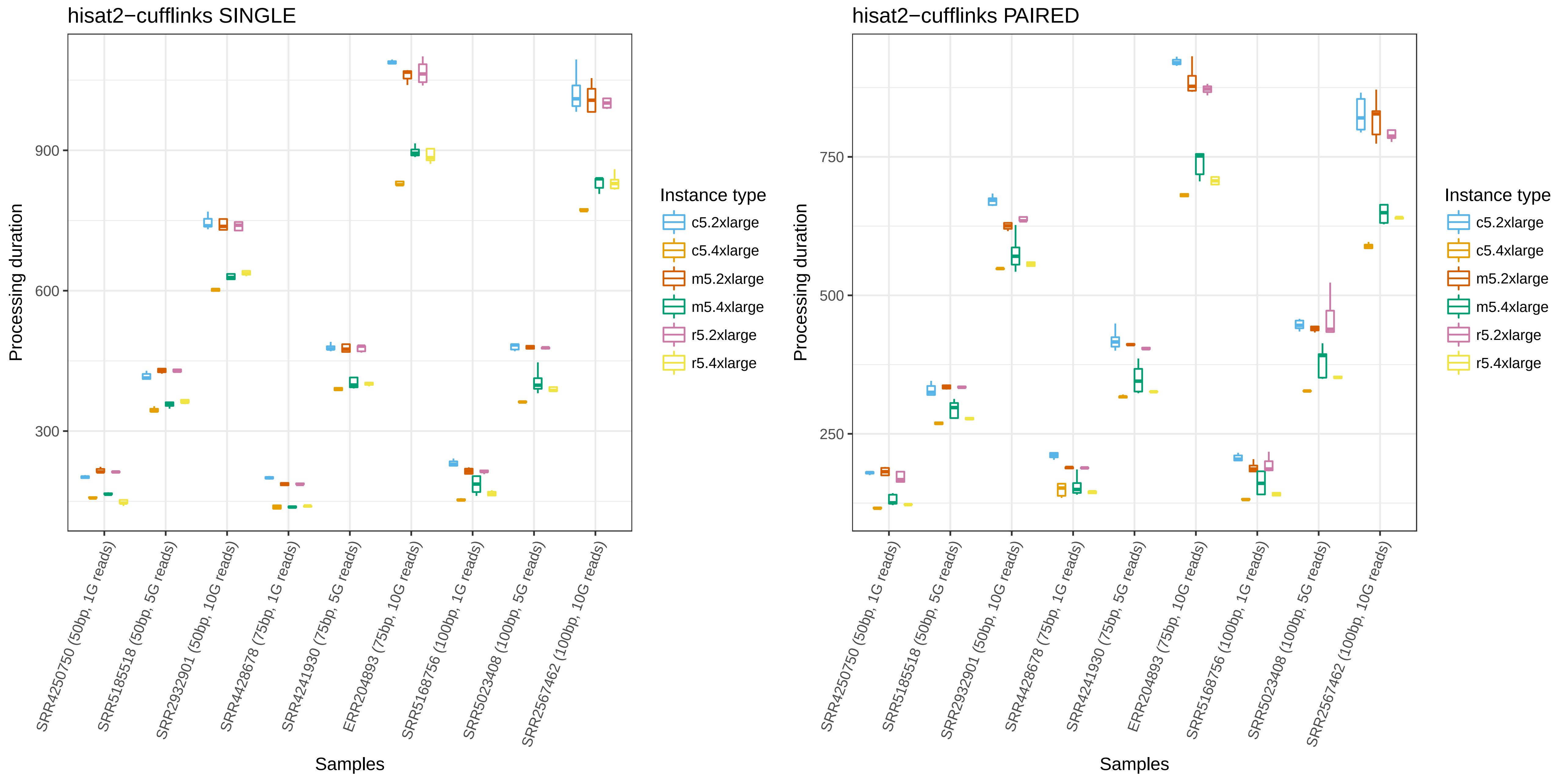
hisat2−cufflinks SINGLE
Processing duration
300
600
900
Samples
SRR4250750 (50bp, 1G reads)
SRR5185518 (50bp, 5G reads)
SRR2932901 (50bp, 10G reads)
SRR4428678 (75bp, 1G reads)
SRR4241930 (75bp, 5G reads)
ERR204893 (75bp, 10G reads)
SRR5168756 (100bp, 1G reads)
SRR5023408 (100bp, 5G reads)
SRR2567462 (100bp, 10G reads)
Instance type
c5.2xlarge
c5.4xlarge
m5.2xlarge
m5.4xlarge
r5.2xlarge
r5.4xlarge
hisat2−cufflinks PAIRED
Processing duration
250
500
750
Samples
SRR4250750 (50bp, 1G reads)
SRR5185518 (50bp, 5G reads)
SRR2932901 (50bp, 10G reads)
SRR4428678 (75bp, 1G reads)
SRR4241930 (75bp, 5G reads)
ERR204893 (75bp, 10G reads)
SRR5168756 (100bp, 1G reads)
SRR5023408 (100bp, 5G reads)
SRR2567462 (100bp, 10G reads)
Instance type
c5.2xlarge
c5.4xlarge
m5.2xlarge
m5.4xlarge
r5.2xlarge
r5.4xlarge

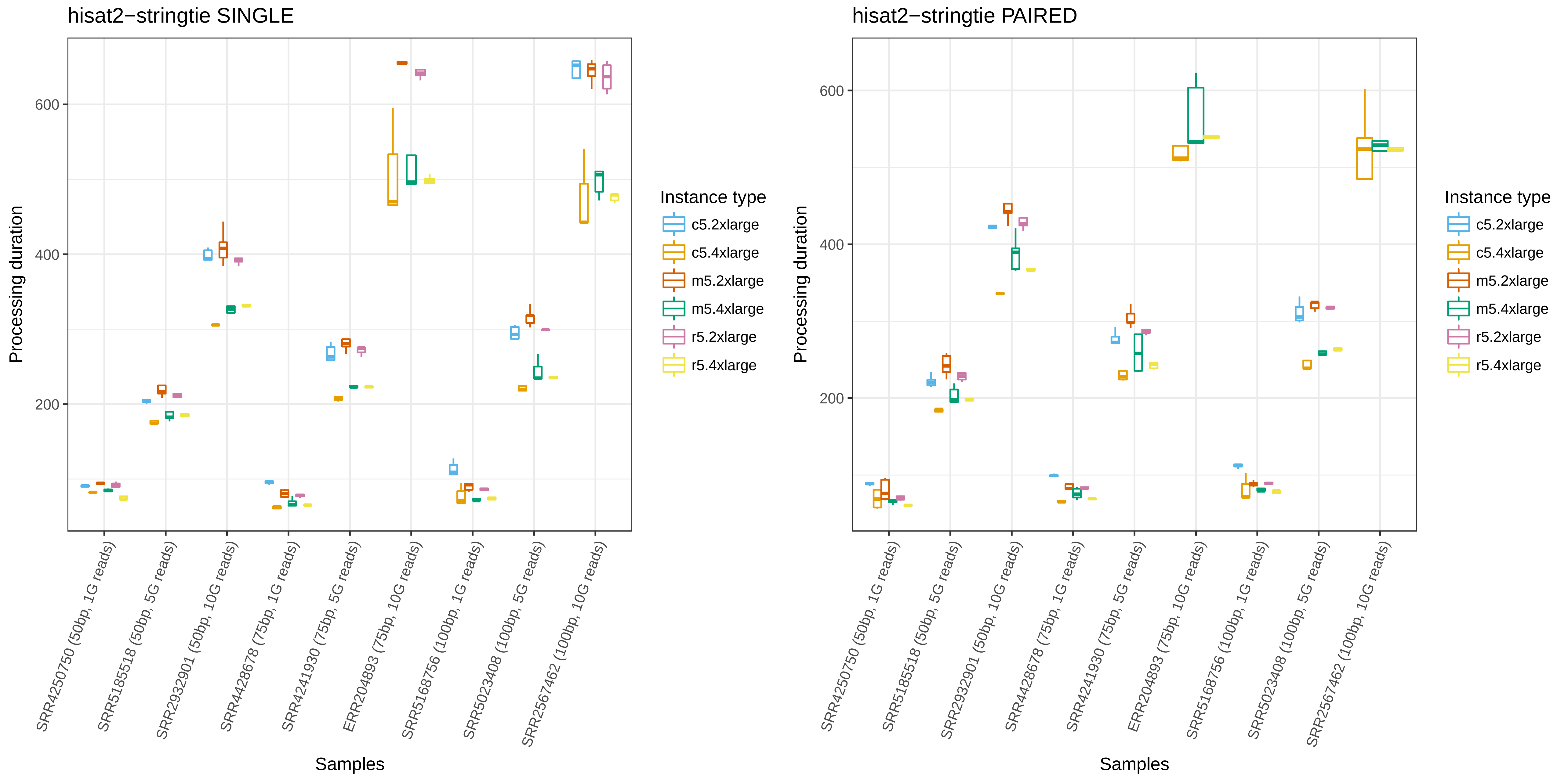

hisat2−stringtie SINGLE
hisat2−stringtie PAIRED
Processing duration
200
400
600
Samples
SRR4250750 (50bp, 1G reads)
SRR5185518 (50bp, 5G reads)
SRR2932901 (50bp, 10G reads)
SRR4428678 (75bp, 1G reads)
SRR4241930 (75bp, 5G reads)
ERR204893 (75bp, 10G reads)
SRR5168756 (100bp, 1G reads)
SRR5023408 (100bp, 5G reads)
SRR2567462 (100bp, 10G reads)
Instance type
c5.2xlarge
c5.4xlarge
m5.2xlarge
m5.4xlarge
r5.2xlarge
r5.4xlarge

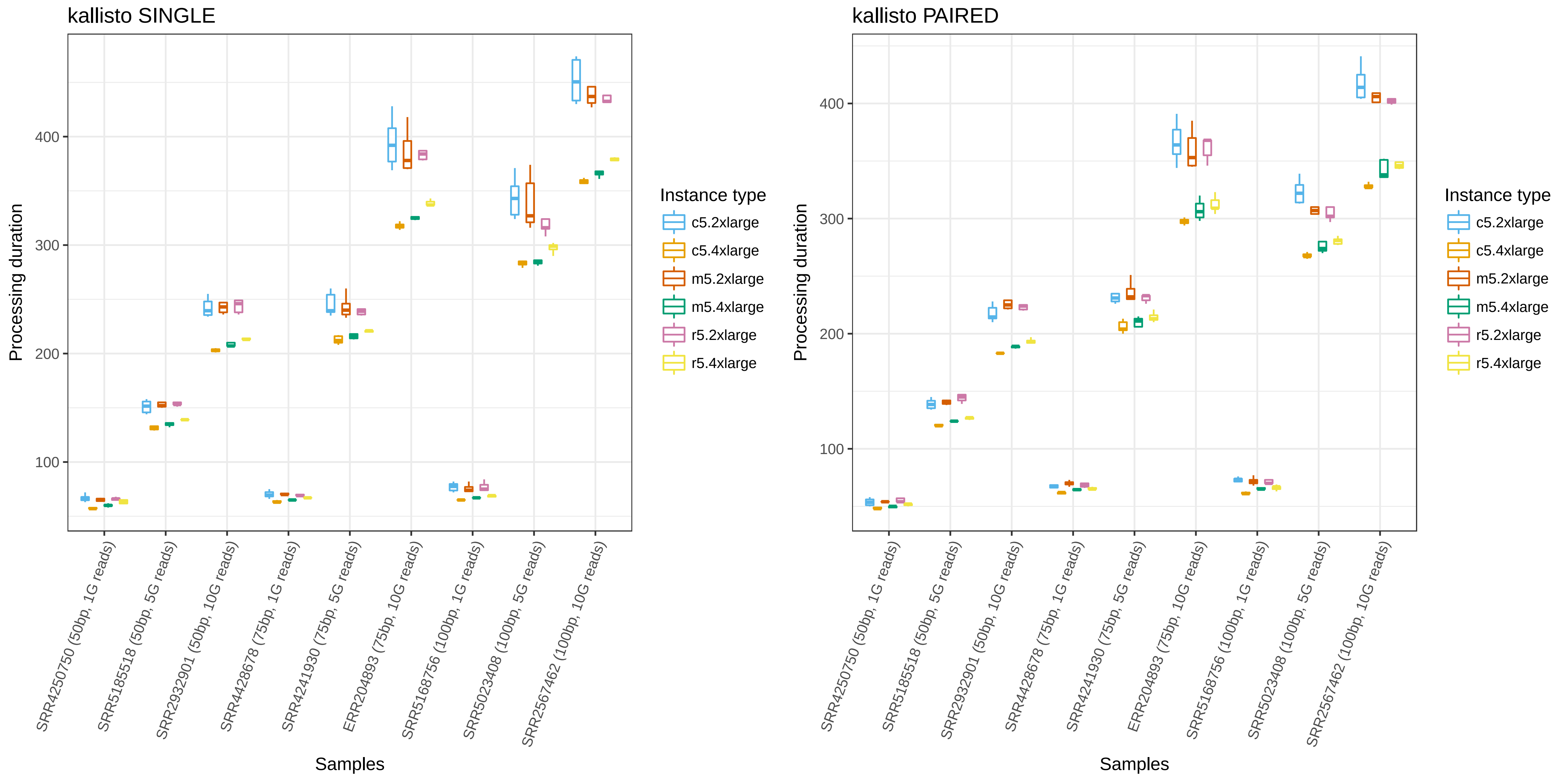
kallisto SINGLE
kallisto PAIRED
Processing duration
Samples
Instance type
c5.2xlarge
c5.4xlarge
m5.2xlarge
m5.4xlarge
r5.2xlarge
r5.4xlarge
100
200
300
400
SRR4250750 (50bp, 1G reads)
SRR5185518 (50bp, 5G reads)
SRR2932901 (50bp, 10G reads)
SRR4428678 (75bp, 1G reads)
SRR4241930 (75bp, 5G reads)
ERR204893 (75bp, 10G reads)
SRR5168756 (100bp, 1G reads)
SRR5023408 (100bp, 5G reads)
SRR2567462 (100bp, 10G reads)

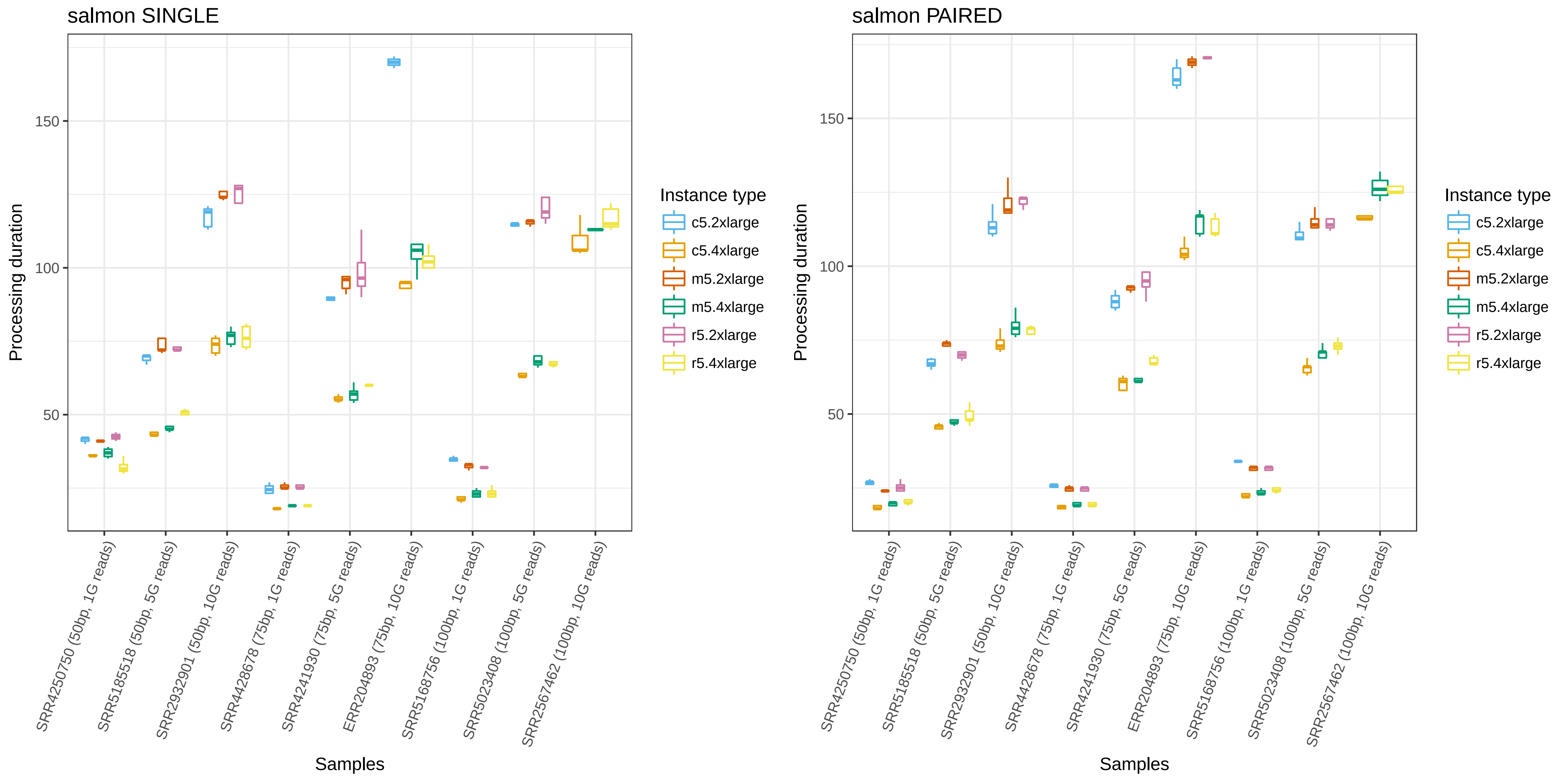
salmon SINGLE
salmon PAIRED
Processing duration
150
100
50
Samples
SRR4250750 (50bp, 1G reads)
SRR5185518 (50bp, 5G reads)
SRR2932901 (50bp, 10G reads)
SRR4428678 (75bp, 1G reads)
SRR4241930 (75bp, 5G reads)
ERR204893 (75bp, 10G reads)
SRR5168756 (100bp, 1G reads)
SRR5023408 (100bp, 5G reads)
SRR2567462 (100bp, 10G reads)
Instance type
c5.2xlarge
c5.4xlarge
m5.2xlarge
m5.4xlarge
r5.2xlarge
r5.4xlarge

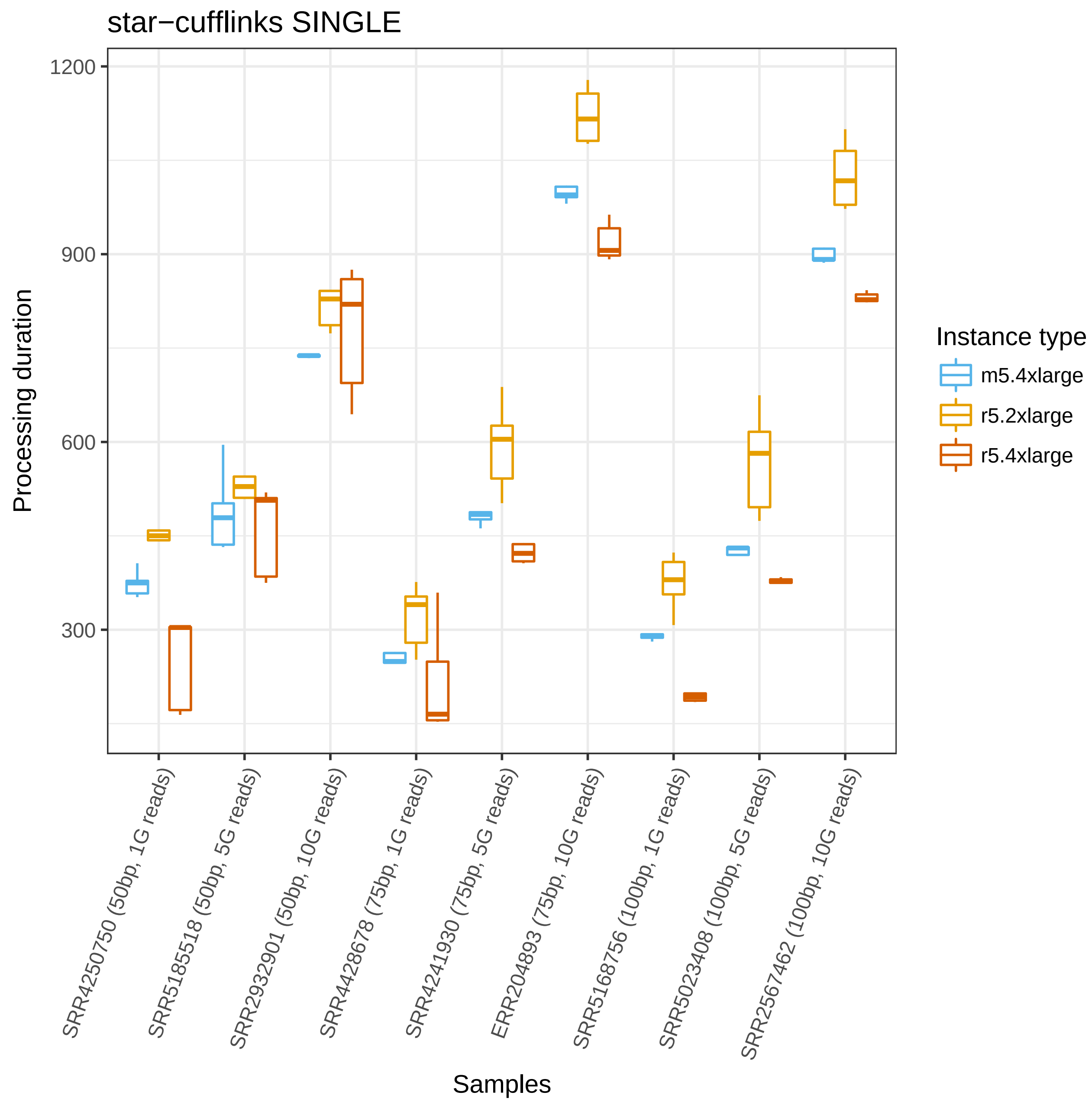
star−cufflinks SINGLE
Processing duration
1200
900
600
300
SRR4250750 (50bp, 1G reads)
SRR5185518 (50bp, 5G reads)
SRR2932901 (50bp, 10G reads)
SRR4428678 (75bp, 1G reads)
SRR4241930 (75bp, 5G reads)
ERR204893 (75bp, 10G reads)
SRR5168756 (100bp, 1G reads)
SRR5023408 (100bp, 5G reads)
SRR2567462 (100bp, 10G reads)
Samples
Instance type
m5.4xlarge
r5.2xlarge
r5.4xlarge

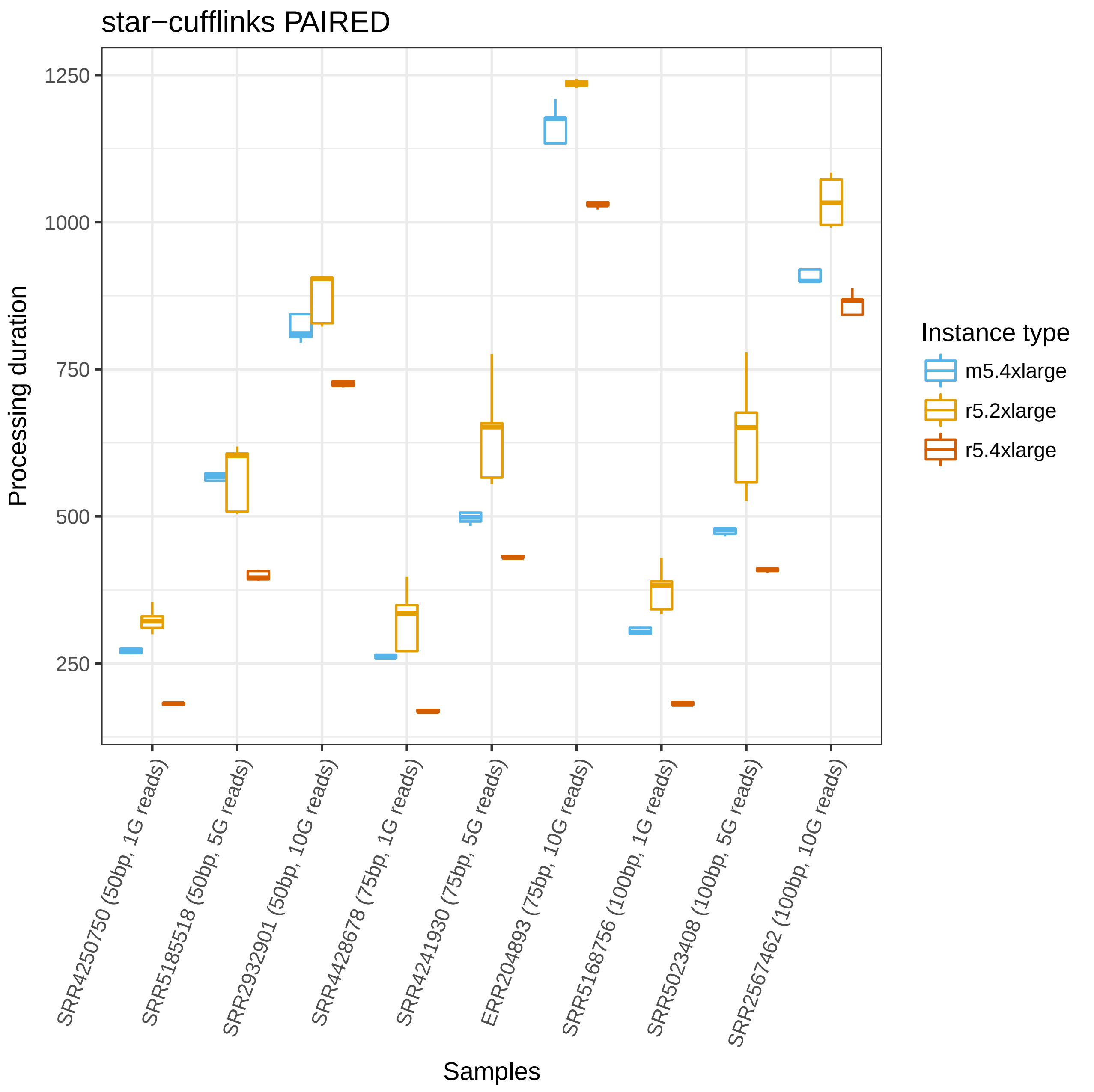
star−cufflinks PAIRED
Processing duration
1250
1000
750
500
250
SRR4250750 (50bp, 1G reads)
SRR5185518 (50bp, 5G reads)
SRR2932901 (50bp, 10G reads)
SRR4428678 (75bp, 1G reads)
SRR4241930 (75bp, 5G reads)
ERR204893 (75bp, 10G reads)
SRR5168756 (100bp, 1G reads)
SRR5023408 (100bp, 5G reads)
SRR2567462 (100bp, 10G reads)
Samples
Instance type
m5.4xlarge
r5.2xlarge
r5.4xlarge

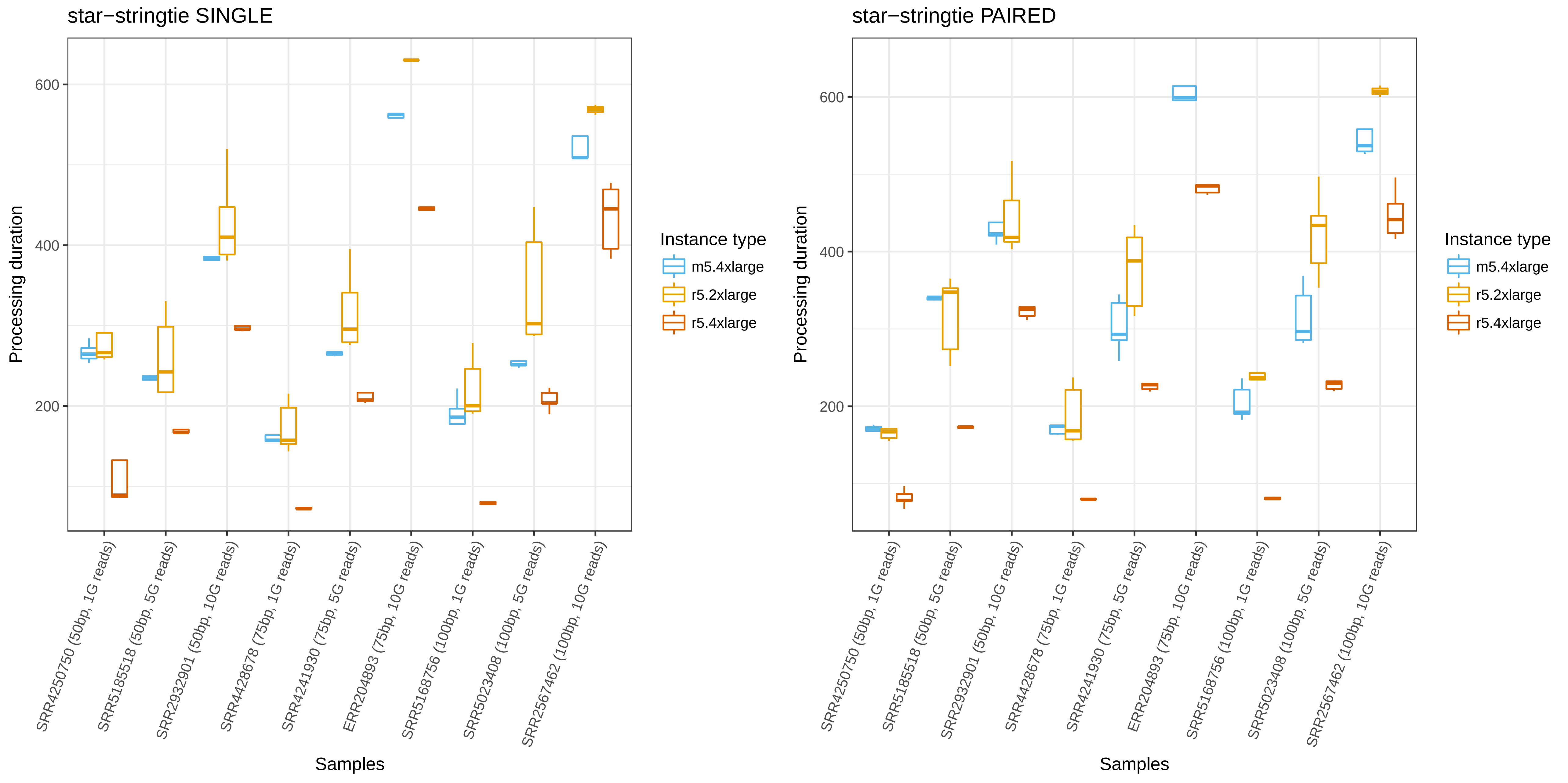
star−stringtie SINGLE
star−stringtie PAIRED
Processing duration
200
400
600
Samples
Instance type
m5.4xlarge
r5.2xlarge
r5.4xlarge
SRR4250750 (50bp, 1G reads)
SRR5185518 (50bp, 5G reads)
SRR2932901 (50bp, 10G reads)
SRR4428678 (75bp, 1G reads)
SRR4241930 (75bp, 5G reads)
ERR204893 (75bp, 10G reads)
SRR5168756 (100bp, 1G reads)
SRR5023408 (100bp, 5G reads)
SRR2567462 (100bp, 10G reads)

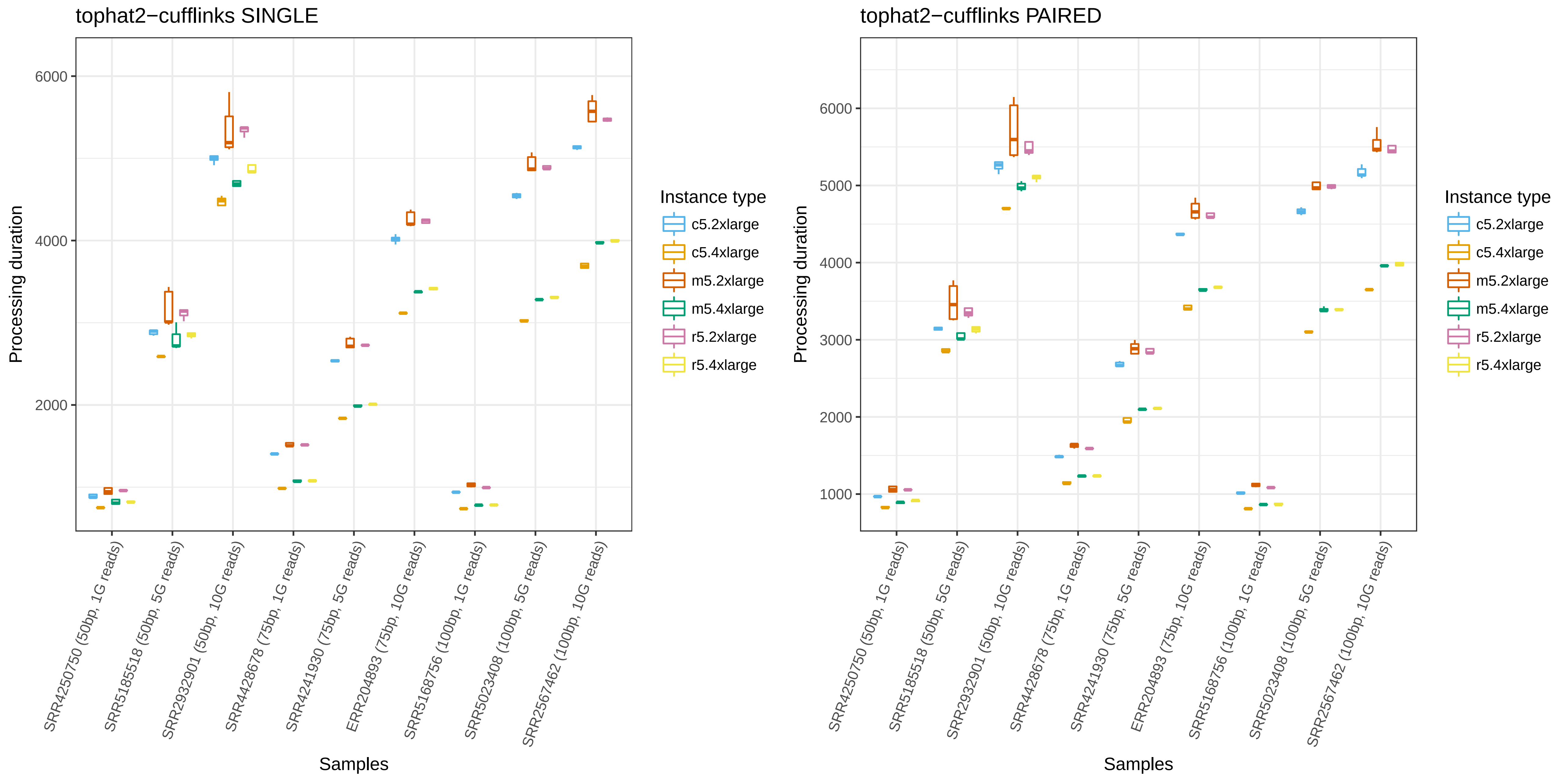
tophat2−cufflinks SINGLE
Processing duration
6000
4000
2000
Samples
SRR4250750 (50bp, 1G reads)
SRR5185518 (50bp, 5G reads)
SRR2932901 (50bp, 10G reads)
SRR4428678 (75bp, 1G reads)
SRR4241930 (75bp, 5G reads)
ERR204893 (75bp, 10G reads)
SRR5168756 (100bp, 1G reads)
SRR5023408 (100bp, 5G reads)
SRR2567462 (100bp, 10G reads)
Instance type
c5.2xlarge
c5.4xlarge
m5.2xlarge
m5.4xlarge
r5.2xlarge
r5.4xlarge
tophat2−cufflinks PAIRED
Processing duration
6000
5000
4000
3000
2000
1000
Samples
SRR4250750 (50bp, 1G reads)
SRR5185518 (50bp, 5G reads)
SRR2932901 (50bp, 10G reads)
SRR4428678 (75bp, 1G reads)
SRR4241930 (75bp, 5G reads)
ERR204893 (75bp, 10G reads)
SRR5168756 (100bp, 1G reads)
SRR5023408 (100bp, 5G reads)
SRR2567462 (100bp, 10G reads)
Instance type
c5.2xlarge
c5.4xlarge
m5.2xlarge
m5.4xlarge
r5.2xlarge
r5.4xlarge
