# Supplementary material for: Accumulating computational resource usage of genomic data analysis workflow to optimize cloud computing instance selection
Source: Gigascience. 2019 Apr 24;8(4):giz052. doi: 10.1093/gigascience/giz052 (PMC6479428; doi:10.1093/gigascience/giz052)
Supplement: Supplemental Files [file giz052_supplemental_files.zip › supp-2.pdf]

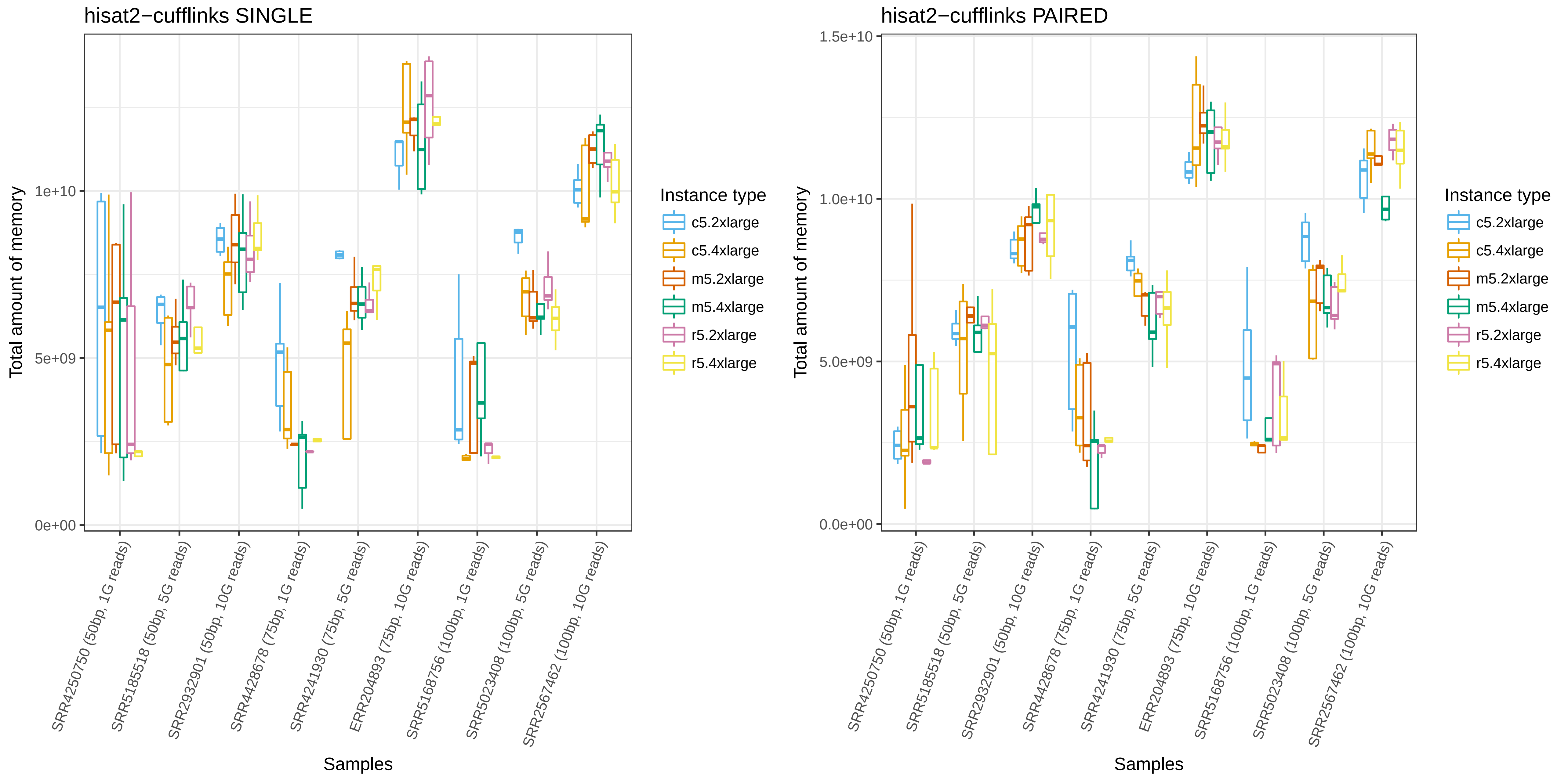
hisat2−cufflinks SINGLE
hisat2−cufflinks PAIRED
Total amount of memory
0e+00
5e+09
1e+10
0.0e+00
5.0e+09
1.0e+10
1.5e+10
Samples
SRR4250750 (50bp, 1G reads)
SRR5185518 (50bp, 5G reads)
SRR2932901 (50bp, 10G reads)
SRR4428678 (75bp, 1G reads)
SRR4241930 (75bp, 5G reads)
ERR204893 (75bp, 10G reads)
SRR5168756 (100bp, 1G reads)
SRR5023408 (100bp, 5G reads)
SRR2567462 (100bp, 10G reads)
Instance type
c5.2xlarge
c5.4xlarge
m5.2xlarge
m5.4xlarge
r5.2xlarge
r5.4xlarge

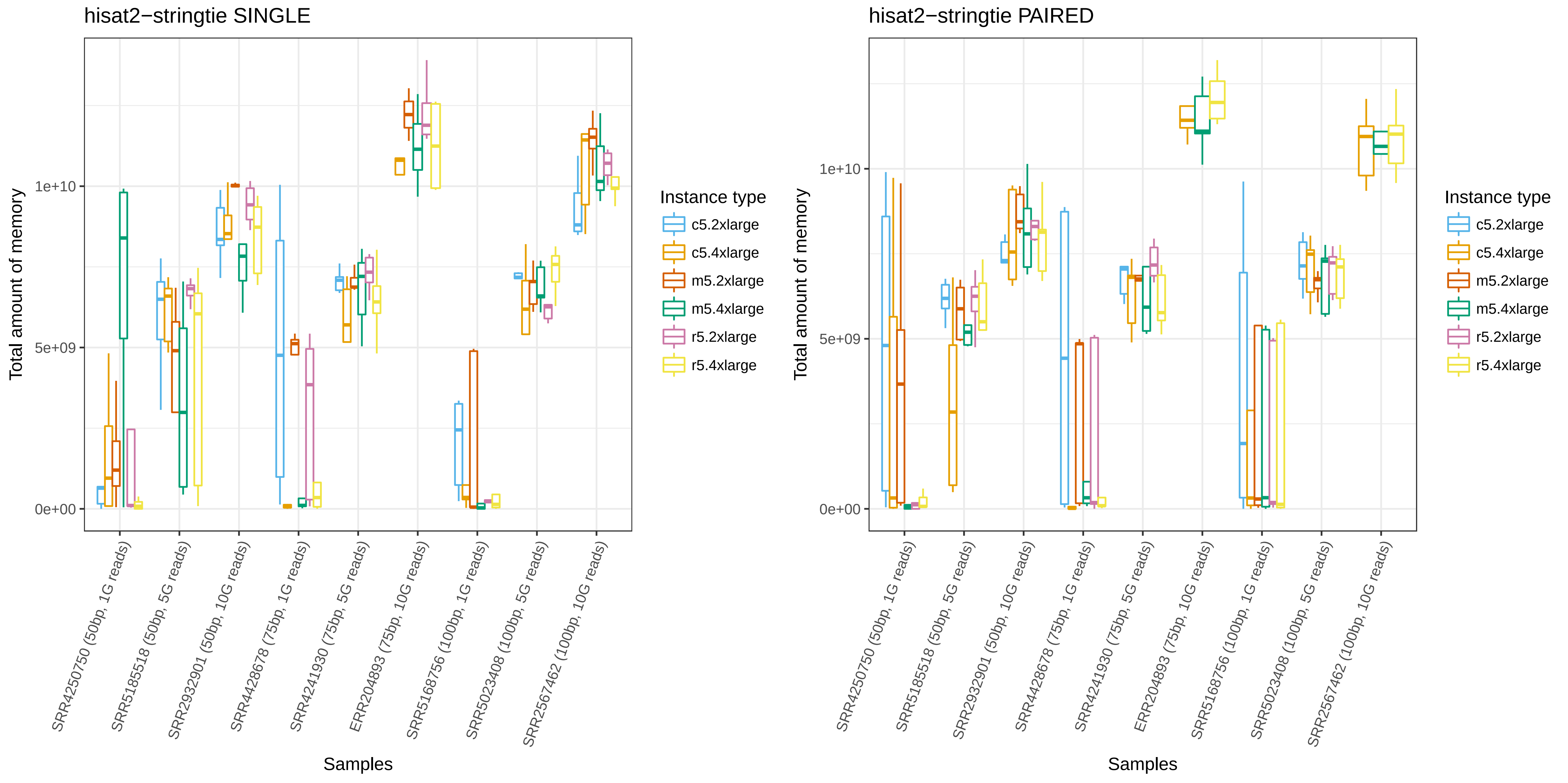
hisat2−stringtie SINGLE
hisat2−stringtie PAIRED
Total amount of memory
0e+00
5e+09
1e+10
Samples
SRR4250750 (50bp, 1G reads)
SRR5185518 (50bp, 5G reads)
SRR2932901 (50bp, 10G reads)
SRR4428678 (75bp, 1G reads)
SRR4241930 (75bp, 5G reads)
ERR204893 (75bp, 10G reads)
SRR5168756 (100bp, 1G reads)
SRR5023408 (100bp, 5G reads)
SRR2567462 (100bp, 10G reads)
Instance type
c5.2xlarge
c5.4xlarge
m5.2xlarge
m5.4xlarge
r5.2xlarge
r5.4xlarge

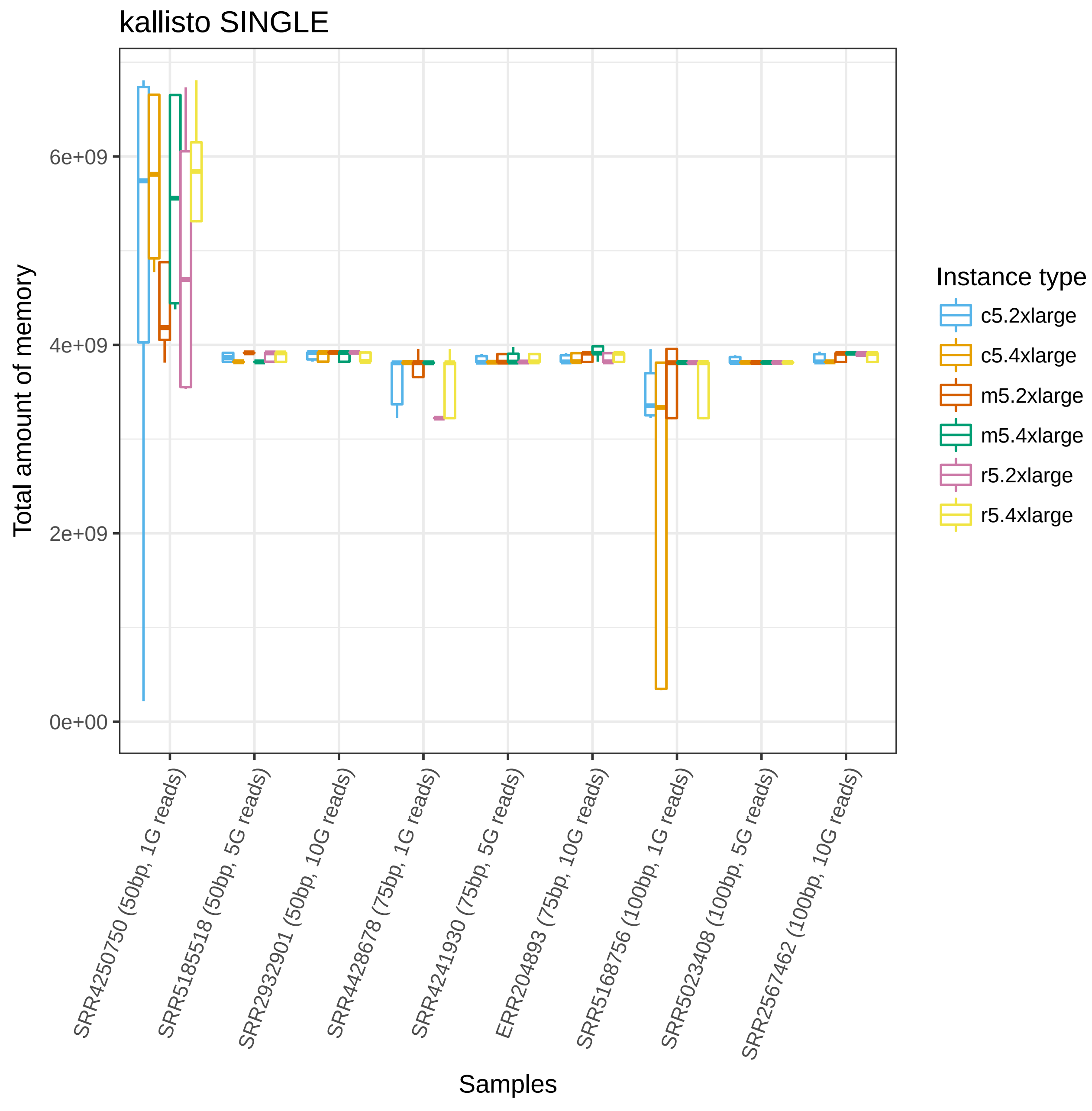
kallisto SINGLE
Total amount of memory
6e+09
4e+09
2e+09
0e+00
Samples
SRR4250750 (50bp, 1G reads)
SRR5185518 (50bp, 5G reads)
SRR2932901 (50bp, 10G reads)
SRR4428678 (75bp, 1G reads)
SRR4241930 (75bp, 5G reads)
ERR204893 (75bp, 10G reads)
SRR5168756 (100bp, 1G reads)
SRR5023408 (100bp, 5G reads)
SRR2567462 (100bp, 10G reads)
Instance type
c5.2xlarge
c5.4xlarge
m5.2xlarge
m5.4xlarge
r5.2xlarge
r5.4xlarge

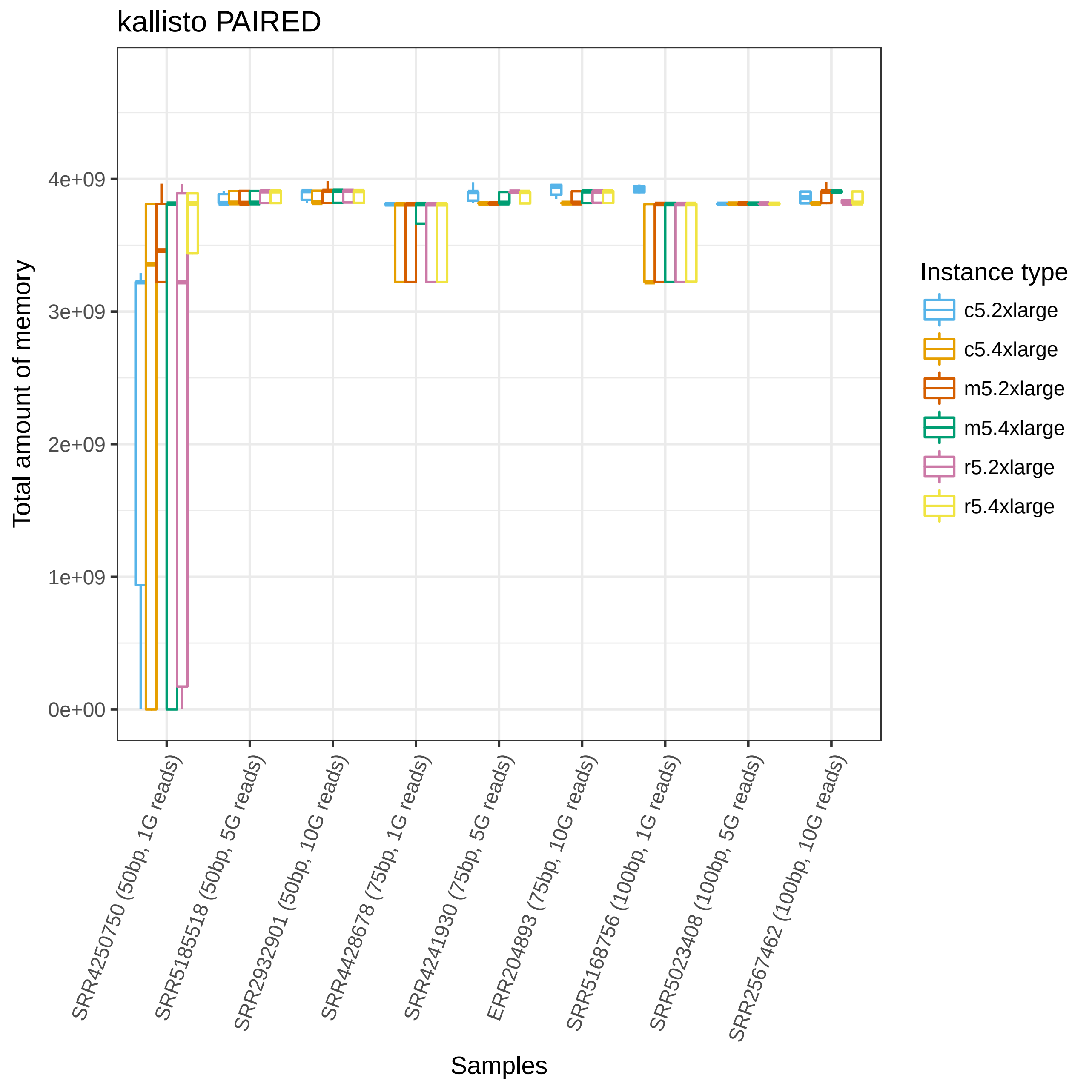
kallisto PAIRED
Total amount of memory
4e+09
3e+09
2e+09
1e+09
0e+00
Samples
SRR4250750 (50bp, 1G reads)
SRR5185518 (50bp, 5G reads)
SRR2932901 (50bp, 10G reads)
SRR4428678 (75bp, 1G reads)
SRR4241930 (75bp, 5G reads)
ERR204893 (75bp, 10G reads)
SRR5168756 (100bp, 1G reads)
SRR5023408 (100bp, 5G reads)
SRR2567462 (100bp, 10G reads)
Instance type
c5.2xlarge
c5.4xlarge
m5.2xlarge
m5.4xlarge
r5.2xlarge
r5.4xlarge

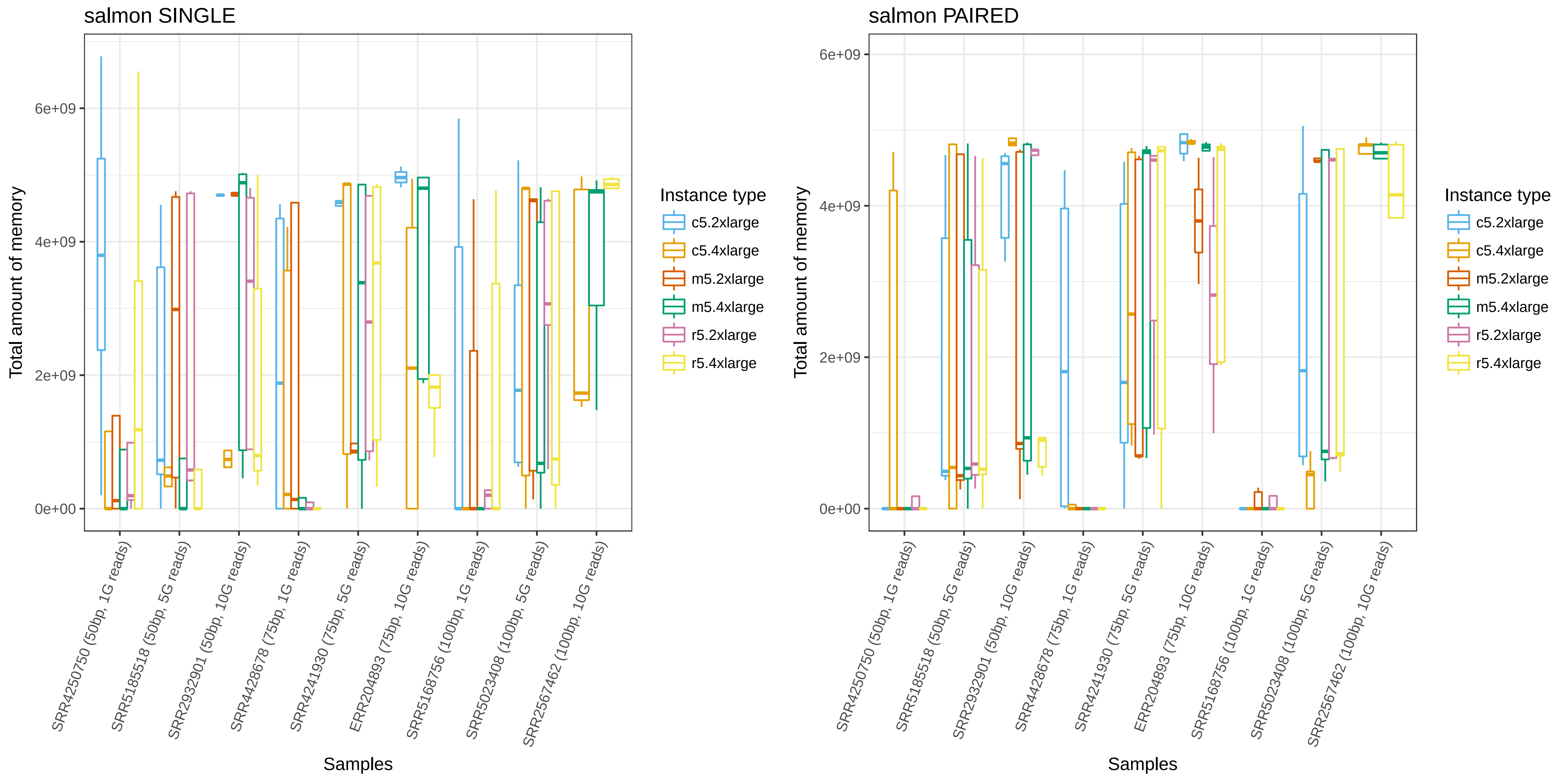

salmon SINGLE
salmon PAIRED
Total amount of memory
0e+00
2e+09
4e+09
6e+09
Samples
SRR4250750 (50bp, 1G reads)
SRR5185518 (50bp, 5G reads)
SRR2932901 (50bp, 10G reads)
SRR4428678 (75bp, 1G reads)
SRR4241930 (75bp, 5G reads)
ERR204893 (75bp, 10G reads)
SRR5168756 (100bp, 1G reads)
SRR5023408 (100bp, 5G reads)
SRR2567462 (100bp, 10G reads)
Instance type
c5.2xlarge
c5.4xlarge
m5.2xlarge
m5.4xlarge
r5.2xlarge
r5.4xlarge

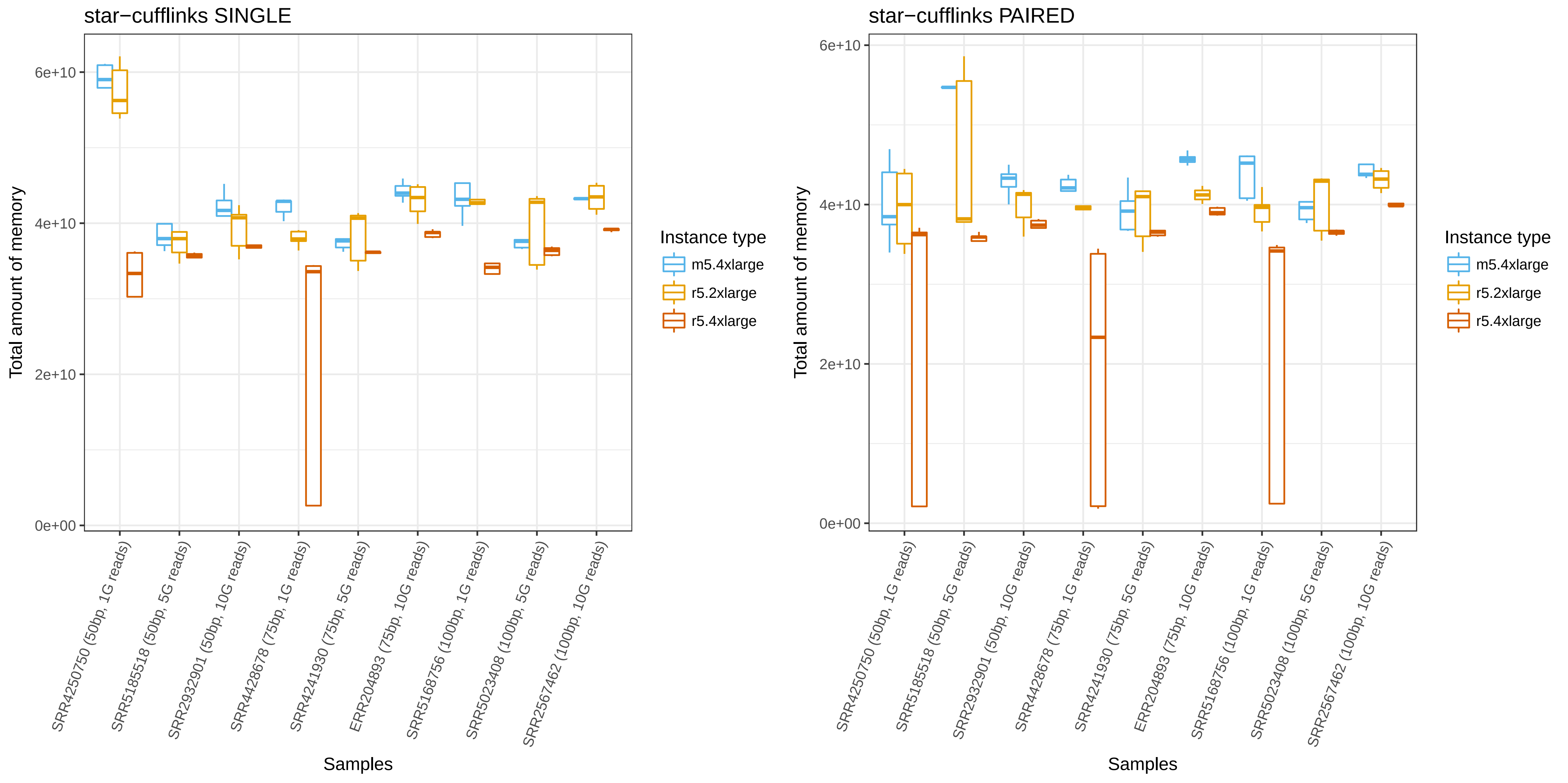
star−cufflinks SINGLE
star−cufflinks PAIRED
Total amount of memory
0e+00
2e+10
4e+10
6e+10
Samples
SRR4250750 (50bp, 1G reads)
SRR5185518 (50bp, 5G reads)
SRR2932901 (50bp, 10G reads)
SRR4428678 (75bp, 1G reads)
SRR4241930 (75bp, 5G reads)
ERR204893 (75bp, 10G reads)
SRR5168756 (100bp, 1G reads)
SRR5023408 (100bp, 5G reads)
SRR2567462 (100bp, 10G reads)
Instance type
m5.4xlarge
r5.2xlarge
r5.4xlarge

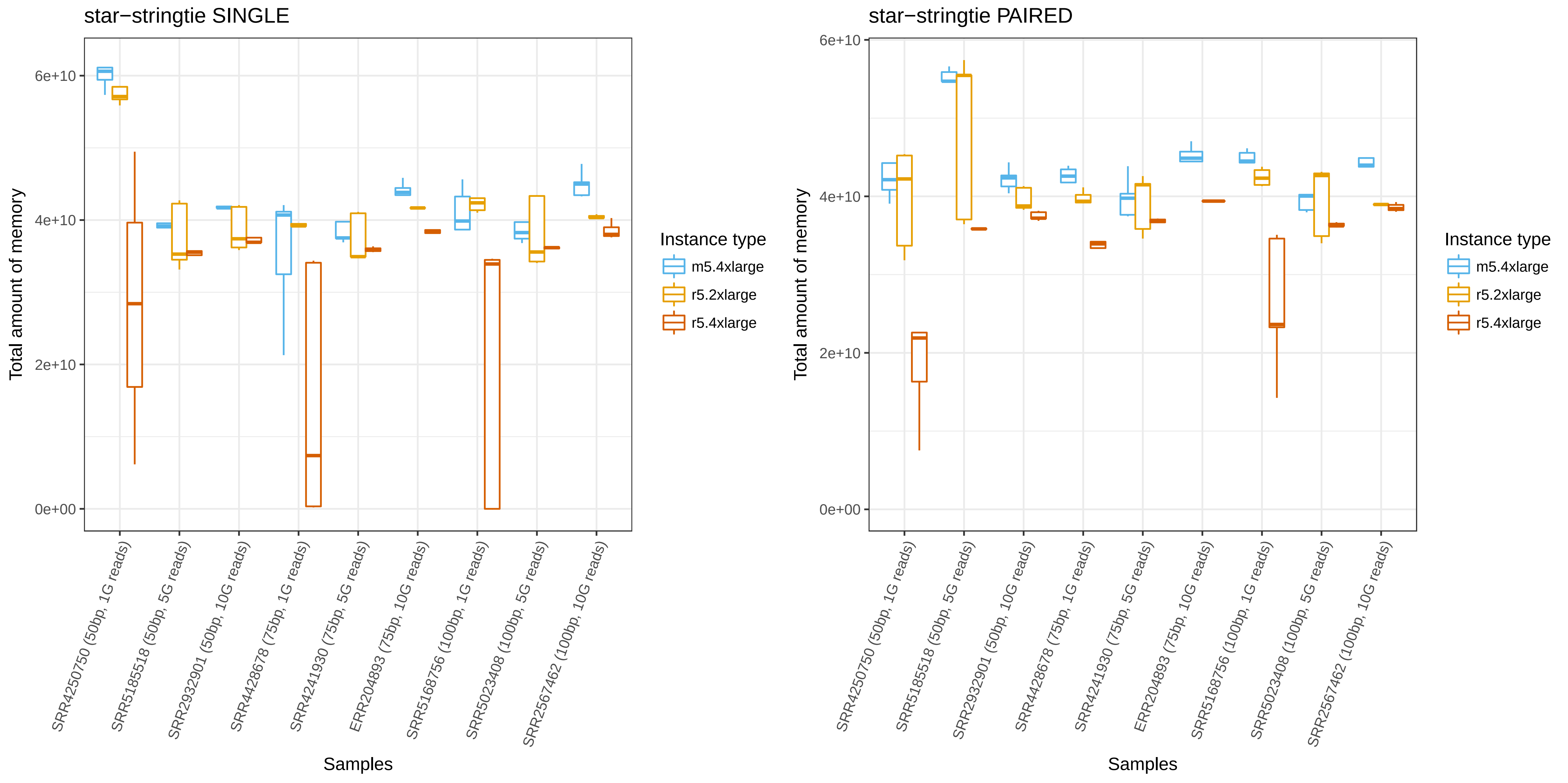

star−stringtie SINGLE
star−stringtie PAIRED
Total amount of memory
0e+00
2e+10
4e+10
6e+10
Samples
SRR4250750 (50bp, 1G reads)
SRR5185518 (50bp, 5G reads)
SRR2932901 (50bp, 10G reads)
SRR4428678 (75bp, 1G reads)
SRR4241930 (75bp, 5G reads)
ERR204893 (75bp, 10G reads)
SRR5168756 (100bp, 1G reads)
SRR5023408 (100bp, 5G reads)
SRR2567462 (100bp, 10G reads)
Instance type
m5.4xlarge
r5.2xlarge
r5.4xlarge

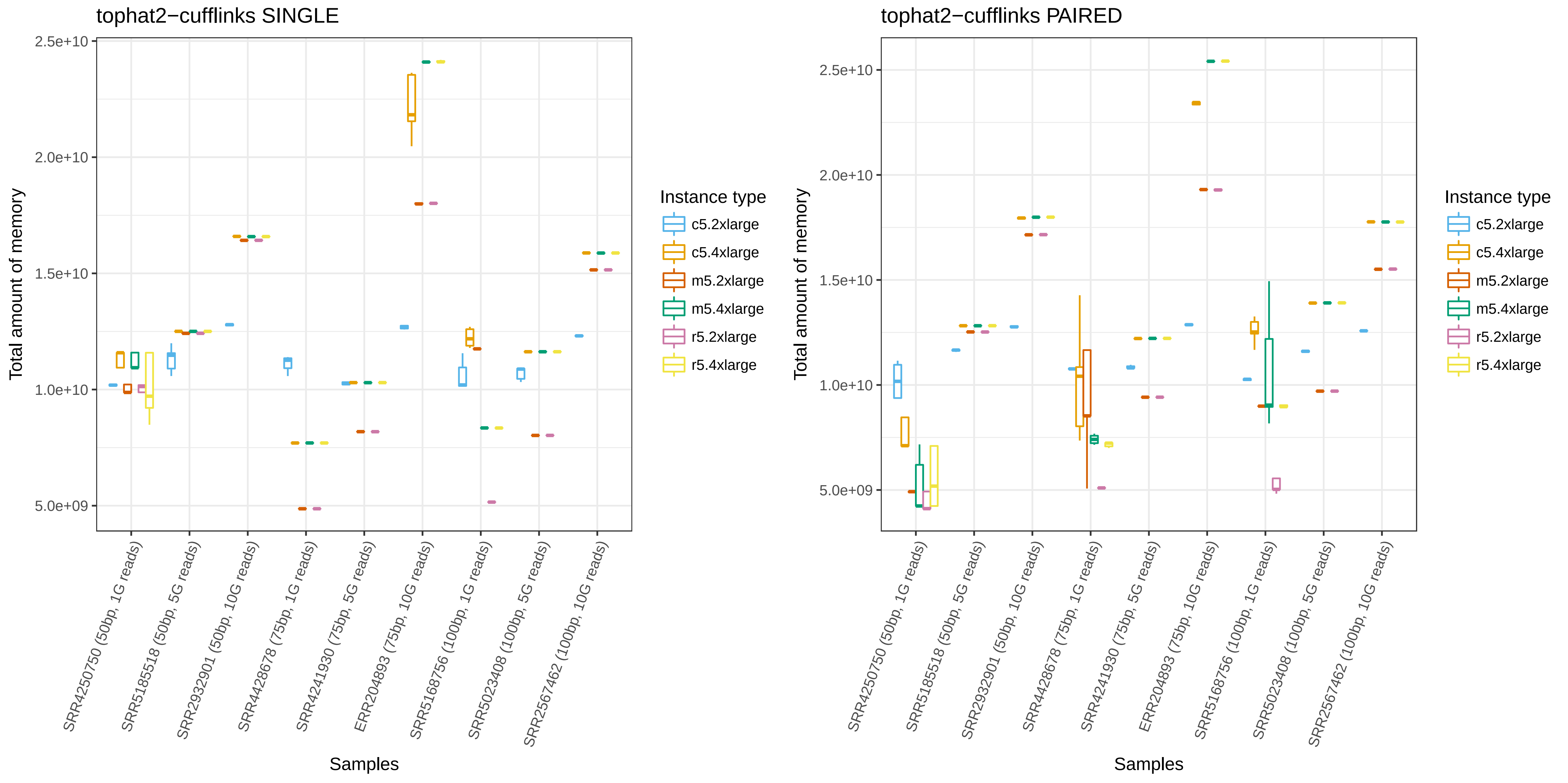

tophat2−cufflinks SINGLE
tophat2−cufflinks PAIRED
Total amount of memory
Samples
Instance type
c5.2xlarge
c5.4xlarge
m5.2xlarge
m5.4xlarge
r5.2xlarge
r5.4xlarge
2.5e+10
2.0e+10
1.5e+10
1.0e+10
5.0e+09
SRR4250750 (50bp, 1G reads)
SRR5185518 (50bp, 5G reads)
SRR2932901 (50bp, 10G reads)
SRR4428678 (75bp, 1G reads)
SRR4241930 (75bp, 5G reads)
ERR204893 (75bp, 10G reads)
SRR5168756 (100bp, 1G reads)
SRR5023408 (100bp, 5G reads)
SRR2567462 (100bp, 10G reads)
