# Supplementary material for: Accumulating computational resource usage of genomic data analysis workflow to optimize cloud computing instance selection
Source: Gigascience. 2019 Apr 24;8(4):giz052. doi: 10.1093/gigascience/giz052 (PMC6479428; doi:10.1093/gigascience/giz052)
Supplement: Response_to_Reviewer_Comments_Original_Submission.pdf [file giz052_response_to_reviewer_comments_original_submission.pdf]

Author's Response To Reviewer Comments

Close

GIGA-D-18-00427
Accumulating computational resource usage of genomic data analysis workflow to optimize cloud computing instance selection
Tazro Ohta; Tomoya Tanjo; Osamu Ogasawara
GigaScience

We deeply thank the reviewers for their positive comments and suggestions. Those suggestions have greatly improved the quality of our project. Our responses are below in-line (the reviewer's comments are in italics).

Response to the reviewers' comments:

Reviewer #1:

Overall the authors provide a useful utility to cwltool that allows for an easy to use collection of runtime metrics. These metrics can be used to make informed decisions on estimating cost and which VM flavors are most efficient. My biggest concern is that the language throughout the manuscript except for a small part of the discussion conveys the idea that this tool is CWL-specific. CWL is simply the specification for defining a workflow and does not define anyway to report logs or runtime metrics which are defined by specific CWL engines. Thus, CWL-metrics is actually dependent on the cwltool reference implementation and the way it currently outputs logs. To me, CWL-metrics is more like an enhancement utility to the cwltool package than a part of CWL itself. I think this is an important distinction to make, especially since cwltool currently only handles serial operation of highly parallelizable workflows. So, if you were really concerned about cost, you likely wouldn't use cwltool as an engine, but look into something like Cromwell, Cavatica (Seven Bridges), or use a GA4GH TES implementation like Funnel. Again, this tool is indeed useful as I have personally used it, I just think that it is important to better clarify this in the manuscript.

Response: We agree with the reviewer's assessment. We started this project to develop a method to provide runtime metrics of CWL workflows so users can share workflows with their resource requirements. The current implementation of CWL-metrics, however, as the reviewer indicates, is the utility tool for cwltool. This is just because the cwltool is the reference implementation of CWL, and will be maintained with the updates of the specification. Yet we think we need to increase the coverage of workflow runners so users can get more practical information. We agree that it is important to make the current status clear. To emphasize the CWL-metrics depends on cwltool, we changed the sentence in the abstract to "We developed CWL-metrics, a utility tool for cwltool, the reference implementation of CWL, to collect runtime metrics of Docker containers and workflow metadata to analyze resource requirement of workflows" and added "CWL-metrics works with cwltool, the reference implementation of CWL" in the last paragraph of the background section.

Related to this, the authors mention Nextflow and Galaxy among others; however, they don't mention WDL or Cromwell which I believe is much more closely related to CWL and I think merit mentioning, especially since Cromwell can run CWL workflows at scale and in parallel.

Response: In the original manuscript, we mentioned Galaxy, Toil, and nextflow because they are the runners that can collect runtime information like CWL-metrics. We added a new paragraph in the discussion section to mention Cromwell and the limitation of cwltool in parallel job execution.

The following are minor things that I believe need to be addressed. The authors use the term "alignment-like" when referring to tools like Kallisto and Salmon; however, I believe the appropriate term is "pseudo-alignment".

Response: The Kallisto is using the term "pseudo-alignment" in its paper and documentation, but the

Salmon does not. The authors of Salmon call its algorithm "quasi-mapping" and did not use the "pseudo-alignment" (https://doi.org/10.1038/nmeth.4197). Therefore we used the term "alignment-like", but it is still confusing. We changed the sentence to call them software use "different alignment approaches".

In figures 4 and 5, I assume the duration y-axis is in seconds; however, this doesn't seem to be mentioned in the description or axis labels and is important especially for people unfamiliar with the workflows.

Response: We added the corresponding units to the Y-axis labels of the plots. Thank you for pointing out the issue.

Finally, I don't feel like the authors provided any clear "future features" they would like to work on (or have the community contribute to). For example, they mention some limitations (e.g., scatter/gather across nodes) that could be overcome by having a centralized service (could be containerized) that all workers post metrics to.

Response: We added a new paragraph in the discussion section with the future prospects including the support of parallelized job execution and the different container runtime like Singularity.

Reviewer #2:

In this paper the authors describe a system to collect execution runtime metrics for computational workflows described using the Common Workflow Language notation. They also provide a benchmark of different tools executed against different dataset to show the benefits for their approach.

The topic is quite interesting because, given the exponential growth of genomics data, there's a pressing need to optimise bioinformatics tools and workflows for better resources allocations and usage in order to optimise the overall costs of long running in-silico data analyses.

The paper is easy to read, well structured and informative. It's particularly interesting the benchmark comparing different genome sequence aligners resource usage.

The only point to make is that the manuscript would capture the interest of a broader audience if the authors would provide a more balanced comparison with similar technologies such as Galaxy and Nextflow. For example the authors mention that their system only work for specific a CWL implementation (cwl-runner) and require the usage of Docker compliant system along with the deployment of other third party tools (eg. Telegraph, Elasticsearch, etc.) which installation could be challenging the average workflow users. Nextflow implements a very similar feature as the one describe in this manuscript to collect, visualise and export the execution metrics. However it can be used irrespective the execution platform supported by the tool (ie. local execution, clusters and clouds), in a single node or multi-node deployment and does not require the installation of any third party software components, either with or without containerised execution (disclaimer, the writer is the creator of the Nextflow tool).

Response: We added a paragraph in the discussion section for a more clear comparison of runners in terms of parallel job execution. The main difference between the cwltool and the other runners that can collect runtime metrics is the ability to capture the parallelized workflow job. We described the limitation of the current implementation of CWL-metrics that depends on cwltool. We want to note that the CWL-metrics users do not need to install Telegraph or Elasticsearch by themselves because the system automatically fetches those components as Docker containers. The prerequisites of the system are git, curl, Perl, Docker, and Docker compose. We added a sentence to mention this in the "Implementation of CWL-metrics" in the result section.

Very minor note, the usage of a human friendly format and data units for time and memory (e.g. seconds or Mega/Giga bytes) values would make the charts more readable.

Response: We added the corresponding units to the Y-axis labels of the plots. Thank you for your suggestion.

- Paolo

Response: Thank you very much, Paolo! Taz :)

Reviewer #3:

This manuscript proposes cwl-metrics, a way to collect metrics from Common Workflow Language executions of bioinformatics tools using Docker containers.

The manuscript is submitted as a Technical Note, but the research is of such a high quality that this could even be a Research article had it also provided a broader Background and a Discussion with comparison of workflow metrics systems beyond CWL.

Response: We submitted this manuscript via the direct submission system through bioRxiv, which did not give us an article type selection. We suppose that the GigaScience editorial office made a decision to make this a Technical Note, but we would be happy to change the category to Research article if possible.

The authors have been diligent in reproducibility and been good practitioners of Open Science, recording rich details of their evaluations and providing installation scripts for not just the software but also the evaluation setup. Some small issues remain to make this truly reproducible.

The English language of the manuscript is however not of a good quality for publication, to a degree where this can be confusing. Knowing the authors work from the CWL community I have provided feedback within the annotated manuscript (attached PDF) using ISO5776 text proof notation.

See the detailed review PDF for further comments according to GigaScience review guidelines, in particular the reproducibility section.

My detailed review is also web-accessible at the (secret) URL
https://gist.github.com/stain/30e49363238d5a35e26f9fb1a31ebf8e

Response: Thank you very much for the many practical suggestions and corrections in detail. The correction of English writing was very helpful. We greatly appreciate your help.

Minor Revisions required:

* English Language needs to be revised

Response: We modified the text following your suggestions and corrections.

* Missing DOIs for citations

Response: We added the DOIs to the reference section.

* Missing DOIs for code/workflows, not (just) GitHub links

Response: For our GitHub repositories, we assigned DOIs and used them in the reference section.

* License on CWL workflows/tools

Response: We added the license to the repository and to the individual files (https://github.com/pitagora-network/pitagora-cwl). The license is Apache-2.0 following the best practice of the Common Workflow Language project.

* Fix Reproducibility issue in Notebook (missing files)

Response: We uploaded the missing files to the Figshare and fixed the Notebook code to fix the issue. We also tested the Notebook correctly works on a new machine environment.

* License and source/upstream attribution for quay.io docker images

Response: We added the GPL-3.0 license for the Docker images we uploaded to Quay.io.

Other suggestions in this review are recommended, but not required.

Response: We also fixed documentations on GitHub to provide more details for workflows and test scripts we provide. The documentation for reference data we used for the benchmark is also available on the new GitHub repo (https://github.com/pitagora-network/pitagora-cwl). We uploaded the reference data index to Zenodo (https://doi.org/10.5281/zenodo.2587201). We also uploaded the intermediate files and final outputs of the workflows executed for the benchmarking to Zenodo (https://doi.org/10.5281/zenodo.2586546). Thank you very much again for your suggestions, we are sure that the CWL-metrics project is now more reproducible.

Close
