# Supplementary material for: Accumulating computational resource usage of genomic data analysis workflow to optimize cloud computing instance selection
Source: Gigascience. 2019 Apr 24;8(4):giz052. doi: 10.1093/gigascience/giz052 (PMC6479428; doi:10.1093/gigascience/giz052)
Supplement: Response_to_Reviewer_Comments_Revision_1.pdf [file giz052_response_to_reviewer_comments_revision_1.pdf]

Author's Response To Reviewer Comments

Close

We have confirmed all the changes suggested in the main manuscript file. We also included tables in the main manuscript file and removed PDF files for them. Thank you very much for your help to improve our manuscript!

Close
