# Supplementary material for: Accumulating computational resource usage of genomic data analysis workflow to optimize cloud computing instance selection
Source: Gigascience. 2019 Apr 24;8(4):giz052. doi: 10.1093/gigascience/giz052 (PMC6479428; doi:10.1093/gigascience/giz052)
Supplement: Reviewer_1_Report_Original_Submission -- Kyle Hernandez, Ph.D. — 11/18/2018 Reviewed [file giz052_reviewer_1_report_original_submission.pdf]

Reviewer Report

Title: Accumulating computational resource usage of genomic data analysis workflow to optimize cloud computing instance selection

Version: Original Submission **Date:** 11/18/2018

Reviewer name: Kyle Hernandez, Ph.D.

Reviewer Comments to Author:

Overall the authors provide a useful utility to cwltool that allows for an easy to use collection of runtime metrics. These metrics can be used to make informed decisions on estimating cost and which VM flavors are most efficient. My biggest concern is that the language throughout the manuscript except for a small part of the discussion conveys the idea that this tool is CWL-specific. CWL is simply the specification for defining a workflow and does not define anyway to report logs or runtime metrics which are defined by specific CWL engines. Thus, CWL-metrics is actually dependent on the cwltool reference implementation and the way it currently outputs logs. To me, CWL-metrics is more like an enhancement utility to the cwltool package than a part of CWL itself. I think this is an important distinction to make, especially since cwltool currently only handles serial operation of highly parallelizable workflows. So, if you were really concerned about cost, you likely wouldn't use cwltool as an engine, but look into something like Cromwell, Cavatica (Seven Bridges), or use a GA4GH TES implementation like Funnel. Again, this tool is indeed useful as I have personally used it, I just think that it is important to better clarify this in the manuscript. Related to this, the authors mention Nextflow and Galaxy among others; however, they don't mention WDL or Cromwell which I believe is much more closely related to CWL and I think merit mentioning, especially since Cromwell can run CWL workflows at scale and in parallel.
The following are minor things that I believe need to be addressed. The authors use the term "alignment-like" when referring to tools like Kallisto and Salmon; however, I believe the appropriate term is "pseudo-alignment". In figures 4 and 5, I assume the duration y-axis is in seconds; however, this doesn't seem to be mentioned in the description or axis labels and is important especially for people unfamiliar with the workflows. Finally, I don't feel like the authors provided any clear "future features" they would like to work on (or have the community contribute to). For example, they mention some limitations (e.g., scatter/gather across nodes) that could be overcome by having a centralized service (could be containerized) that all workers post metrics to.

Methods

Are the methods appropriate to the aims of the study, are they well described, and are necessary controls included? Choose an item.

Conclusions

Are the conclusions adequately supported by the data shown? Choose an item.

Reporting Standards

Does the manuscript adhere to the journal's guidelines on [minimum standards of reporting?] Choose an item.

Choose an item.

Statistics

Are you able to assess all statistics in the manuscript, including the appropriateness of statistical tests used? Choose an item.

Quality of Written English

Please indicate the quality of language in the manuscript: Choose an item.

Declaration of Competing Interests

Please complete a declaration of competing interests, considering the following questions:

- Have you in the past five years received reimbursements, fees, funding, or salary from an organisation that may in any way gain or lose financially from the publication of this manuscript, either now or in the future?
- Do you hold any stocks or shares in an organisation that may in any way gain or lose financially from the publication of this manuscript, either now or in the future?
- Do you hold or are you currently applying for any patents relating to the content of the manuscript?
- Have you received reimbursements, fees, funding, or salary from an organization that holds or has applied for patents relating to the content of the manuscript?
- Do you have any other financial competing interests?
- Do you have any non-financial competing interests in relation to this paper?

If you can answer no to all of the above, write 'I declare that I have no competing interests' below. If your reply is yes to any, please give details below.

I declare that I have no competing interests

I agree to the open peer review policy of the journal. I understand that my name will be included on my report to the authors and, if the manuscript is accepted for publication, my named report including any attachments I upload will be posted on the website along with the authors' responses. I agree for my report to be made available under an Open Access Creative Commons CC-BY license (http://creativecommons.org/licenses/by/4.0/). I understand that any comments which I do not wish to be included in my named report can be included as confidential comments to the editors, which will not be published.

I agree to the open peer review policy of the journal

To further support our reviewers, we have joined with Publons, where you can gain additional credit to further highlight your hard work (see: https://publons.com/journal/530/gigascience). On publication of this paper, your review will be automatically added to Publons, you can then choose whether or not to claim your Publons credit. I understand this statement.

Yes Choose an item.
