# Supplementary material for: Accumulating computational resource usage of genomic data analysis workflow to optimize cloud computing instance selection
Source: Gigascience. 2019 Apr 24;8(4):giz052. doi: 10.1093/gigascience/giz052 (PMC6479428; doi:10.1093/gigascience/giz052)
Supplement: Reviewer_2_Report_Original_Submission -- Paolo Di Tommaso — 12/9/2018 Reviewed [file giz052_reviewer_2_report_original_submission.pdf]

Reviewer Report

Title: Accumulating computational resource usage of genomic data analysis workflow to optimize cloud computing instance selection

Version: Original Submission **Date:** 12/9/2018

Reviewer name: Paolo Di Tommaso

Reviewer Comments to Author:

In this paper the authors describe a system to collect execution runtime metrics for computational workflows described using the Common Workflow Language notation. They also provide a benchmark of different tools executed against different dataset to show the benefits for their approach.
The topic is quite interesting because, given the exponential growth of genomics data, there's a pressing need to optimise bioinformatics tools and workflows for better resources allocations and usage in order to optimise the overall costs of long running in-silico data analyses.
The paper is easy to read, well structured and informative. It's particularly interesting the benchmark comparing different genome sequence aligners resource usage.
The only point to make is that the manuscript would capture the interest of a broader audience if the authors would provide a more balanced comparison with similar technologies such as Galaxy and Nextflow. For example the authors mention that their system only work for specific a CWL implementation (cwl-runner) and require the usage of Docker compliant system along with the deployment of other third party tools (eg. Telegraph, Elasticsearch, etc.) which installation could be challenging the average workflow users. Nextflow implements a very similar feature as the one describe in this manuscript to collect, visualise and export the execution metrics. However it can be used irrespective the execution platform supported by the tool (ie. local execution, clusters and clouds), in a single node or multi-node deployment and does not require the installation of any third party software components, either with or without containerised execution (disclaimer, the writer is the creator of the Nextflow tool).
Very minor note, the usage of a human friendly format and data units for time and memory (e.g. seconds or Mega/Giga bytes) values would make the charts more readable.
- Paolo

Methods

Are the methods appropriate to the aims of the study, are they well described, and are necessary controls included? Choose an item.

Conclusions

Are the conclusions adequately supported by the data shown? Choose an item.

Reporting Standards

Does the manuscript adhere to the journal's guidelines on [minimum standards of reporting?]() Choose an item.

Choose an item.

Statistics

Are you able to assess all statistics in the manuscript, including the appropriateness of statistical tests used? Choose an item.

Quality of Written English

Please indicate the quality of language in the manuscript: Choose an item.

Declaration of Competing Interests

Please complete a declaration of competing interests, considering the following questions:

- Have you in the past five years received reimbursements, fees, funding, or salary from an organisation that may in any way gain or lose financially from the publication of this manuscript, either now or in the future?
- Do you hold any stocks or shares in an organisation that may in any way gain or lose financially from the publication of this manuscript, either now or in the future?
- Do you hold or are you currently applying for any patents relating to the content of the manuscript?
- Have you received reimbursements, fees, funding, or salary from an organization that holds or has applied for patents relating to the content of the manuscript?
- Do you have any other financial competing interests?
- Do you have any non-financial competing interests in relation to this paper?

If you can answer no to all of the above, write 'I declare that I have no competing interests' below. If your reply is yes to any, please give details below.

I declare that I have no competing interests

I agree to the open peer review policy of the journal. I understand that my name will be included on my report to the authors and, if the manuscript is accepted for publication, my named report including any attachments I upload will be posted on the website along with the authors' responses. I agree for my report to be made available under an Open Access Creative Commons CC-BY license (http://creativecommons.org/licenses/by/4.0/). I understand that any comments which I do not wish to be included in my named report can be included as confidential comments to the editors, which will not be published.

I agree to the open peer review policy of the journal

To further support our reviewers, we have joined with Publons, where you can gain additional credit to further highlight your hard work (see: https://publons.com/journal/530/gigascience). On publication of this paper, your review will be automatically added to Publons, you can then choose whether or not to claim your Publons credit. I understand this statement.

Yes Choose an item.
