# Supplementary material for: Accumulating computational resource usage of genomic data analysis workflow to optimize cloud computing instance selection
Source: Gigascience. 2019 Apr 24;8(4):giz052. doi: 10.1093/gigascience/giz052 (PMC6479428; doi:10.1093/gigascience/giz052)
Supplement: Reviewer_3_Report_Original_Submission -- Stian Soiland-Reyes — 12/18/2018 Reviewed [file giz052_reviewer_3_report_original_submission.pdf]

Reviewer Report

Title: Accumulating computational resource usage of genomic data analysis workflow to optimize cloud computing instance selection

Version: Original Submission **Date:** 12/18/2018

Reviewer name: Stian Soiland-Reyes

Reviewer Comments to Author:

This manuscript proposes cwl-metrics, a way to collect metrics from Common Workflow Language executions of bioinformatics tools using Docker containers.
The manuscript is submitted as a Technical Note, but the research is of such a high quality that this could even be a Research article had it also provided a broader Background and a Discussion with comparison of workflow metrics systems beyond CWL.
The authors have been diligent in reproducibility and been good practitioners of Open Science, recording rich details of their evaluations and providing installation scripts for not just the software but also the evaluation setup. Some small issues remain to make this truly reproducible.
The English language of the manuscript is however not of a good quality for publication, to a degree where this can be confusing. Knowing the authors work from the CWL community I have provided feedback within the annotated manuscript (attached PDF) using ISO5776 text proof notation.
See the detailed review PDF for further comments according to GigaScience review guidelines, in particular the reproducibility section.
My detailed review is also web-accessible at the (secret) URL
https://gist.github.com/stain/30e49363238d5a35e26f9fb1a31ebf8e
Minor Revisions required:
* English Language needs to be revised
* Missing DOIs for citations
* Missing DOIs for code/workflows, not (just) GitHub links
* License on CWL workflows/tools
* Fix Reproducibility issue in Notebook (missing files)
* License and source/upstream attribution for quay.io docker images
Other suggestions in this review are recommended, but not required.

Methods

Are the methods appropriate to the aims of the study, are they well described, and are necessary controls included? Choose an item.

Conclusions

Are the conclusions adequately supported by the data shown? Choose an item.

Reporting Standards

Does the manuscript adhere to the journal's guidelines on [minimum standards of reporting?] Choose an item.

Choose an item.

Statistics

Are you able to assess all statistics in the manuscript, including the appropriateness of statistical tests used? Choose an item.

Quality of Written English

Please indicate the quality of language in the manuscript: Choose an item.

Declaration of Competing Interests

Please complete a declaration of competing interests, considering the following questions:

- Have you in the past five years received reimbursements, fees, funding, or salary from an organisation that may in any way gain or lose financially from the publication of this manuscript, either now or in the future?
- Do you hold any stocks or shares in an organisation that may in any way gain or lose financially from the publication of this manuscript, either now or in the future?
- Do you hold or are you currently applying for any patents relating to the content of the manuscript?
- Have you received reimbursements, fees, funding, or salary from an organization that holds or has applied for patents relating to the content of the manuscript?
- Do you have any other financial competing interests?
- Do you have any non-financial competing interests in relation to this paper?

If you can answer no to all of the above, write 'I declare that I have no competing interests' below. If your reply is yes to any, please give details below.

I am a member of the leadership team for Common Workflow Language at the Software Freedom Conservancy non-profit, but have no financial interest.

I agree to the open peer review policy of the journal. I understand that my name will be included on my report to the authors and, if the manuscript is accepted for publication, my named report including any attachments I upload will be posted on the website along with the authors' responses. I agree for my report to be made available under an Open Access Creative Commons CC-BY license (http://creativecommons.org/licenses/by/4.0/). I understand that any comments which I do not wish to be included in my named report can be included as confidential comments to the editors, which will not be published.

I agree to the open peer review policy of the journal

To further support our reviewers, we have joined with Publons, where you can gain additional credit to further highlight your hard work (see: https://publons.com/journal/530/gigascience). On publication of this paper, your review will be automatically added to Publons, you can then choose whether or not to claim your Publons credit. I understand this statement.

Yes Choose an item.
